# Sulfate radical anion-induced benzylic oxidation of *N*-(arylsulfonyl)benzylamines to *N*-arylsulfonylimines

**DOI:** 10.3762/bjoc.19.57

**Published:** 2023-06-05

**Authors:** Joydev K Laha, Pankaj Gupta, Amitava Hazra

**Affiliations:** 1 Department of Pharmaceutical Technology (Process Chemistry), National Institute of Pharmaceutical Education and Research, S. A. S. Nagar, Punjab 160062, India

**Keywords:** arylsulfonylimine, benzylic oxidation, benzyl sulfonamide, K_2_S_2_O_8_, sulfate radical anion

## Abstract

A mild, operationally convenient, and practical method for the synthesis of synthetically useful *N*-arylsulfonylimines from *N*-(arylsulfonyl)benzylamines using K_2_S_2_O_8_ in the presence of pyridine as a base is reported herein. In addition, a “one-pot” tandem synthesis of pharmaceutically relevant *N*-heterocycles by the reaction of *N*-arylsulfonylimines, generated in situ with *ortho*-substituted anilines is also reported. The key features of the protocol include the use of a green oxidant, a short reaction time (30 min), chromatography-free isolation, scalability, and economical, delivering *N*-arylsulfonylimines in excellent yields of up to 96%. While the oxidation of *N*-aryl(benzyl)amines to *N*-arylimines using K_2_S_2_O_8_ is reported to be problematic, the oxidation of *N*-(arylsulfonyl)benzylamines to *N*-arylsulfonylimines using K_2_S_2_O_8_ has been achieved for the first time. The dual role of the sulfate radical anion (SO_4_^·−^), including hydrogen atom abstraction (HAT) and single electron transfer (SET), is proposed to be involved in the plausible reaction mechanism.

## Introduction

**Scheme 1:**
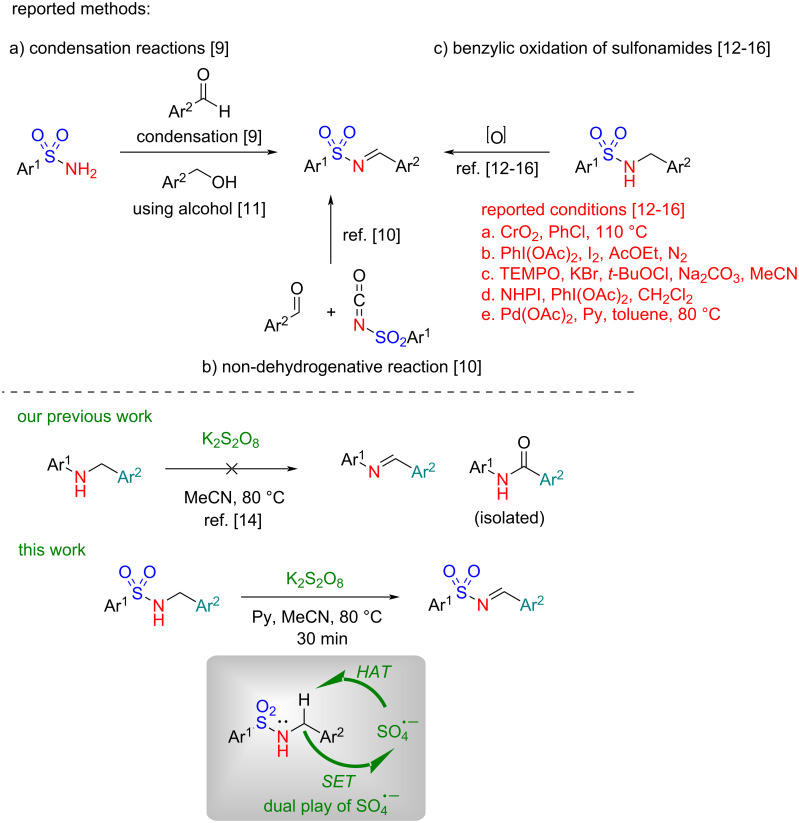
Various synthetic approaches to *N*-arylsulfonylimines.

Among various imine compounds [1], *N*-arylsulfonylimines are perhaps the most prominent due to their unique stability, defined reactivity, and versatility in organic synthesis [2]. Leveraging their electron-deficient nature, *N*-arylsulfonylimines are widely used in organic transformations including nucleophilic addition, cycloaddition, imino-aldol reaction, ene reactions, aza-Friedel–Crafts reactions, and C–H functionalizations ([3] and references therein), leading to the synthesis of diverse nitrogen heterocycles of pharmaceutical relevance [4]. The traditional synthetic method for the preparation of *N*-arylsulfonylimines, similar to the preparation of *N*-arylimines, is based on the condensation of aromatic aldehydes and sulfonamides (Scheme 1a) [3,5–8]. Because of the poor nucleophilicity of sulfonamides, the condensation reactions generally require harsh reaction conditions involving the use of strong acids, elevated temperature, and metal catalysts. Other methods include a non-dehydrative reaction of aldehydes with isocyanate analogs ([3] and references therein) (Scheme 1b) and an oxidative reaction of primary benzylic alcohols with sulfonamides or chloramine-T ([3] and references therein), and although they are elegant, they use substrates that are not readily accessible or toxic in nature. To overcome these limitations, oxidation of *N*-(arylsulfonyl)benzylamines to *N*-arylsulfonylimines, as opposed to the traditional methods, under mild and neutral reaction conditions has been reported, although limited to a few methods. However, these methods of oxidation involving the use of CrO_2_ [9], PhI(OAc)_2_/I_2_ [10], TEMPO [11], NHPI [12], and metal catalysts [13], suffer from serious limitations including the use of metal catalysts, high temperature, risk of explosive hazards, production of large waste, and often low yield (Scheme 1c). Thus, an environmentally benign method that could deliver *N*-arylsulfonylimines under mild reaction conditions is highly desirable.

Previously, we reported a tandem oxidative intramolecular cyclization of *N*-aryl(benzyl)amines, having an internal nucleophile substituted at the *ortho*-position in the aniline ring, to nitrogen heterocycles using potassium persulfate (K_2_S_2_O_8_) as the exclusive reagent [14]. The mechanistic study revealed that an initial oxidation to an iminium ion could be the key intermediate in the intramolecular cyclization step. In sharp contrast, when *N*-aryl(benzyl)amines that do not have an *ortho*-substituted nucleophile in aniline ring were used as the substrates in this reaction, *N*-arylimines were not isolated. Rather, an amide, in some cases, was isolated via oxidation of the benzylic methylene to a carbonyl group [14]. In the quest of a new method for the synthesis of *N*-arylsulfonylimines, we questioned ourselves whether *N*-(arylsulfonyl)benzylamines would behave similarly as *N*-aryl(benzyl)amines under K_2_S_2_O_8_-mediated oxidative conditions and could provide a platform for the synthesis of *N*-arylsulfonylimines.

To this endeavor, we have developed a method for the synthesis of *N*-arylsulfonylimines from *N*-(arylsulfonyl)benzylamines using K_2_S_2_O_8_ in the presence of pyridine as a base. The key findings include a) requirement of a mild base for the formation *N*-arylsulfonylimines, and b) stability of *N*-arylsulfonylimines, unlike *N*-arylimines, under the oxidative conditions. Further, to demonstrate the scope and applicability of this approach, a gram-scale synthesis and a “one-pot” tandem synthesis of pharmaceutically relevant *N*-heterocycles by the reaction of in situ-generated *N*-arylsulfonylimines with various *ortho*-substituted anilines were also developed. The mechanism of the oxidation is believed to occur via hydrogen atom abstraction (HAT) followed by single electron transfer (SET) enabled by the sulfate radical anion (SO_4_^·−^).

## Results and Discussion

Initially, we investigated the reaction of *N*-benzenesulfonyl(benzyl)amine (**1a**) as a model substrate with K_2_S_2_O_8_ in MeCN at 80 °C for 12 h, conditions that were used earlier in our previous study [14]. Unfortunately, no product formation was observed under these conditions, while substrate **1a** remained unreacted (Table 1, entry 1). When the solvent was changed to H_2_O, a trace quantity of product formation was observed (Table 1, entry 2). To our surprise, when 2 equiv of pyridine were used as an additive along with the oxidant K_2_S_2_O_8_ in MeCN, the desired product *N*-benzenesulfonylimine **2a** was obtained in 90% yield (Table 1, entry 3). Subsequently, we carried out further optimization studies by changing the additive, solvent, temperature, and reaction time to obtain the best possible yield of the product **2a** (Table 1). Interestingly, when duration of the reaction was reduced to 1 h, product **2a** was obtained in 96% yield with complete conversion of substrate **1a** (Table 1, entry 4). Further shortening the reaction time to 30 min resulted in the formation of **2a** also in 96% yield (Table 1, entry 5). Lowering the temperature to 60 °C had a deleterious effect (Table 1, entry 6). Likewise, reducing the stoichiometry of pyridine to 1 equiv proved detrimental (Table 1, entry 7). Replacing pyridine with other organic and inorganic bases such as Et_3_N, DBU, DABCO or K_2_CO_3_ also gave product **2a**, however, in varying yields (Table 1, entries 8–11). While replacing the solvent MeCN with DCE delivered **2a** in 89% yield, and a dramatic reduction in the yield of **2a** was observed when H_2_O was used as the solvent (Table 1, entries 12 and 13). Therefore, the conditions listed in entry 5 of Table 1 were chosen as the best conditions for further evaluating the substrate scope. Unlike the oxidation of *N*-aryl(benzyl)amines to *N*-arylimines using K_2_S_2_O_8_ in the presence or absence of a base is unsuccessful, the oxidation of *N*-arylsulfonyl(benzyl)amines **1a** to imines **2a** was achieved under the optimized conditions. Distinctly, the use of a base is the key to success in this oxidation. Perhaps more importantly, the stability of *N*-benzenesulfonylimine **2a**, unlike *N*-arylimines, under the oxidative conditions is noteworthy.

**Table 1:** Optimization of reaction conditions.^a^

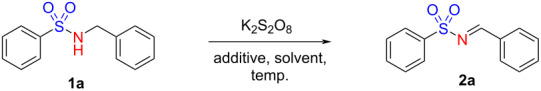					

Entry	Additive (equiv)	Solvent	Temp. (°C)	Time (h)	Yield (%)^b^

1	–	MeCN	80	12	n.d.
2	–	H_2_O	80	12	trace
3	pyridine (2)	MeCN	80	12	90
4	pyridine (2)	MeCN	80	1	96
5	pyridine (2)	MeCN	80	0.5	96
6	pyridine (2)	MeCN	60	1	40
7	pyridine (1)	MeCN	80	1	80
8	Et_3_N (2)	MeCN	80	1	60
9	DBU (2)	MeCN	80	1	92
10	DABCO (2)	MeCN	80	1	90
11	K_2_CO_3_ (2)	MeCN	80	1	75
12	pyridine (2)	DCE	80	1	89
13	pyridine (2)	H_2_O	80	1	20

^a^Reaction conditions: **1a** (0.25 mmol), K_2_S_2_O_8_ (0.5 mmol), additive (0.5 mmol) in solvent (1 mL) at 80 °C for the specified period of time. n.d. = not detected. ^b^Isolated yield.

**Scheme 2:**
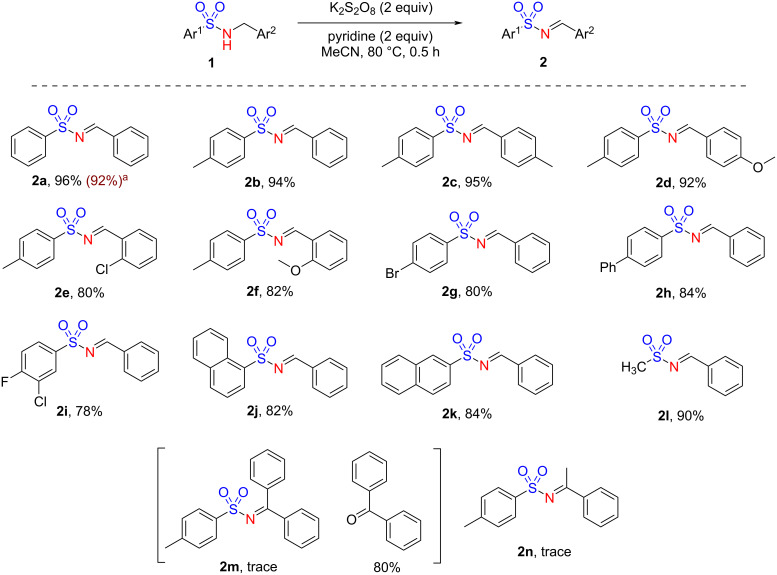
Substrate scope for the synthesis of *N*-arylsulfonylimines. Reaction conditions: **1a** (0.25 mmol), K_2_S_2_O_8_ (0.5 mmol), pyridine (0.5 mmol) in MeCN (1 mL) at 80 °C for 0.5 h. Yields refer to isolated compounds. ^a^Gram-scale synthesis (**1a**, 5 mmol).

With the optimized reaction conditions in hand (Table 1, entry 5), we further investigated the substrate scope for the above transformation (Scheme 2). A limited variety of *N*-(arylsulfonyl)benzylamines **1a**–**m** carrying substitutions on the aromatic rings was examined. Firstly, *N*-(arylsulfonyl)benzylamines having substitution(s) on one or both rings delivered the *N*-arylsulfonylimines **2a**–**h** in 80–96% yield. The presence of a disubstitution in **1i** gave product **2i** in 78% yield. Replacing phenyl with naphthyl in *N*-(arylsulfonyl)benzylamines **1j** and **1k** resulted in the formation of *N*-arylsulfonylimines **2j** and **2k** also in very good yield (82–84%). Interestingly, when the arylsulfonyl group was replaced by methylsulfonyl, as in substrate **1l**, the desired *N*-sulfonylimine **2l** was obtained in 90% yield under the optimized reaction conditions. However, an attempted synthesis of *N*-arylsulfonylketimines was unsuccessful. Thus, *N*-(arylsulfonyl)benzylamine **1m** having a phenyl substituent at the benzylic position gave benzophenone in 80% yield with a trace of *N*-benzenesulfonylketimine **2m** under the optimized reaction conditions. Likewise, *N*-(arylsulfonyl)benzylamine **1n** having a methyl group present at the benzylic position gave product **2n** only in a trace quantity. To demonstrate further the scalability of the developed protocol, we carried out a gram-scale synthesis of **2a** from **1a** under the optimized reaction conditions. A complete conversion of substrate **1a** was observed within 2 h under the optimized reaction conditions giving the product with an isolated yield of 92%.

**Scheme 3:**
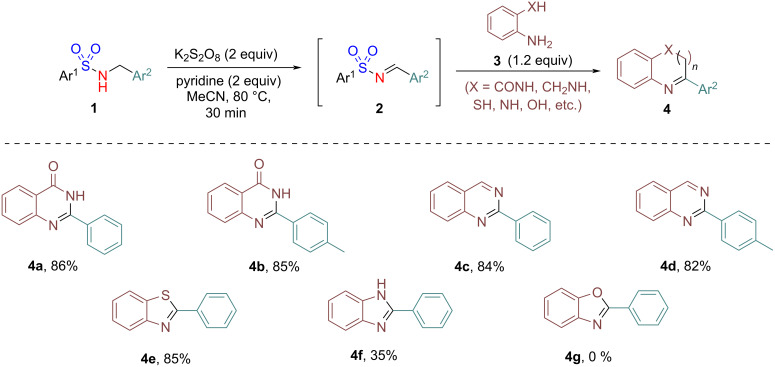
Tandem “one-pot” synthesis of *N*-heterocycles. Reaction conditions: **1a** (0.25 mmol), K_2_S_2_O_8_ (0.5 mmol), and pyridine (0.5 mmol) in MeCN (1mL) at 80 °C for 0.5 h followed by the addition of 1 equiv of K_2_S_2_O_8_ and the corresponding *ortho*-substituted anilines **3** (1.2 equiv) and stirring at 80 °C for 2 h. Yields correspond to isolated products.

Furthermore, to demonstrate the synthetic utility of the developed protocol, a tandem “one-pot” synthesis of *N*-heterocycles was successfully executed (Scheme 3). Thus, exposition of substrates **1** under the optimized reaction conditions followed by the addition of *ortho*-substituted anilines **3** and K_2_S_2_O_8_ (1 equiv) and heating the reaction mixture at 80 °C for 2 h furnished the desired *N*-heterocycles **4**. Thus, treatment of substrate **1a** under the standard conditions, followed by reaction of the intermediate *N*-benzenesulfonylimine **2a** with 2-aminobenzamide in one-pot gave 2-phenylquinazolin-4(3*H*)-one (**4a**) in 86% yield. Similarly, the reaction of the intermediate product **2c** and 2-aminobenzamide gave 2-(*p*-tolyl)quinazolin-4(3*H*)-one (**4b**) in 85% yield.

Furthermore, when various other *ortho*-substituted aniline derivatives such as 2-aminobenzylamine, 2-aminothiophenol, and *o*-phenylenediamine are reacted with imine **2a** in a similar manner, the corresponding *N*-heterocycles **4c**–**f** were obtained in good to moderate yield. However, the reaction with 2-aminophenol did not give the corresponding cyclized product **4g**. This could be possibly due to the poor nucleophilicity of the *ortho*-OH group in 2-aminophenol thereby restricting the intramolecular nucleophilic addition and as a result the corresponding cyclized product is not formed. The synthesis of these nitrogen heterocycles signifies the innate ability of in situ-generated *N*-arylsulfonylimines in a variety of reactions with various *ortho*-substituted anilines without the need for pre-isolation or purification.

**Scheme 4:**
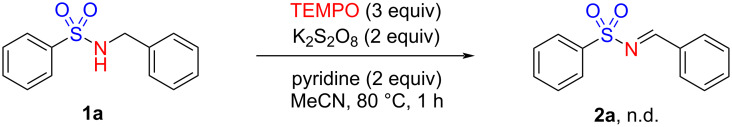
Control experiment with TEMPO.

Next, in order to determine whether the reaction proceeds via a radical pathway, we performed a control experiment. When substrate **1a** was treated with the radical scavenger TEMPO under the optimized reaction conditions, the formation of product **2a** was completely suppressed (Scheme 4). This confirms that the reaction proceeds via a radical pathway.

**Scheme 5:**
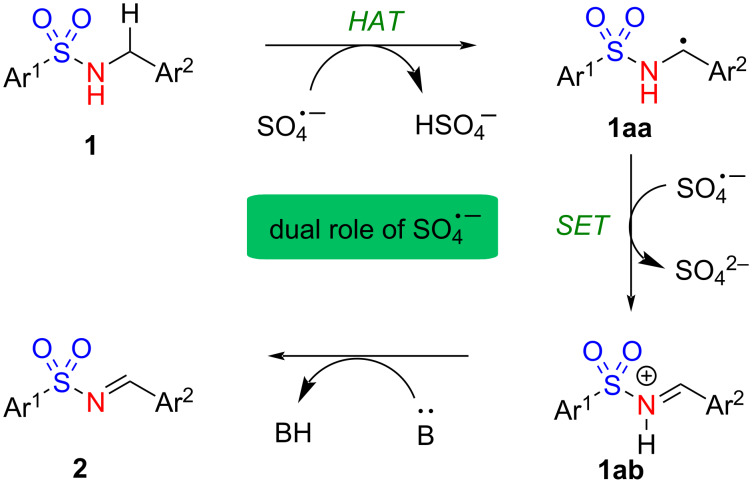
Plausible mechanism for the K_2_S_2_O_8_-induced oxidation of *N*-(arylsulfonyl)benzylamines.

Based on the literature [15–16], our previous experience [14,17–18], and current understanding, a plausible mechanism for the benzylic oxidation is depicted in Scheme 5. Initially, a sulfate radical anion (SO_4_^·−^) is generated by homolytic cleavage of the peroxy linkage under heating conditions [17]. The hydrogen atom is abstracted from the benzylic position of **1** by SO_4_^·−^, generating benzylic radical **1aa** [14–16]. A single electron transfer (SET) could subsequently occur from **1aa** to form the reactive species **1ab**. Finally, the base abstracts the activated NH proton to produce imine **2**. The dual role of SO_4_^·−^ involving HAT and SET is proposed in this plausible mechanism, which requires further investigation.

**Scheme 6:**
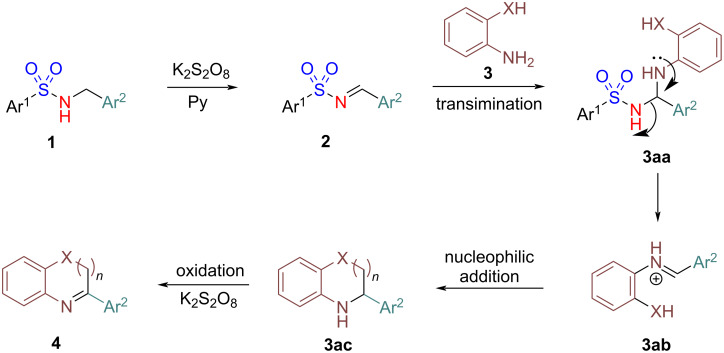
Plausible mechanism for one-pot synthesis of *N*-heterocycles.

Similarly, a plausible mechanism for the one-pot synthesis of *N*-heterocycles is shown in Scheme 6. Initially, the *N*-arylsulfonylimine **2**, generated in situ from the corresponding *N*-(arylsulfonyl)benzylamine **1**, undergoes transimination with the *ortho*-substituted aniline **3** to form imine **3ab** via **3aa**. Subsequent intramolecular nucleophilic addition in imine **3ab** produces intermediate **3ac**, which upon oxidation delivers the desired *N*-heterocycle **4**.

## Conclusion

In conclusion, we have developed a complementary approach to the currently available methods for the oxidation of *N*-(arylsulfonyl)benzylamines to *N*-arylsulfonylimines using K_2_S_2_O_8_ and pyridine as a base. While *N*-arylimines are difficult to prepare by the oxidation of *N*-aryl(benzyl)amines using K_2_S_2_O_8_, *N*-arylsulfonylimines are successfully prepared and are quite stable under the oxidative conditions. In addition, we demonstrated a “one-pot” tandem synthesis of pharmaceutically relevant *N*-heterocycles through the reaction of in situ-generated *N*-arylsulfonylimines with *ortho*-substituted anilines. The key features including the use of a green oxidant, a short reaction time, chromatography-free isolation, and scalability mark a distinction from the contemporary methods. Although we propose a dual role for SO_4_^·−^ involving both hydrogen atom abstraction (HAT) and single electron transfer (SET), further investigation of the mechanism would enrich our understanding of persulfate-mediated oxidative reactions.

## Supporting Information

File 1 General procedures, product characterization, and copies of ^1^H NMR and ^13^C NMR spectra of all compounds.
