# Supplementary material for: Sulfate radical anion-induced benzylic oxidation of N-(arylsulfonyl)benzylamines to N-arylsulfonylimines
Source: Beilstein J Org Chem. 2023 Jun 5;19:771–7. doi: 10.3762/bjoc.19.57 (PMC10280053; doi:10.3762/bjoc.19.57)

BEILSTEIN JOURNAL OF ORGANIC CHEMISTRY

Supporting Information

for

Sulfate radical anion-induced benzylic oxidation of *N*-(arylsulfonyl)benzylamines to *N*-arylsulfonylimines

Joydev K. Laha, Pankaj Gupta and Amitava Hazra

Beilstein J. Org. Chem. **2023,** *19,* 771–777. doi:10.3762/bjoc.19.57

General procedures, product characterization, and copies of ^{1}H NMR and ^{13}C NMR spectra of all compounds

License and Terms: This is a supporting information file under the terms of the Creative Commons Attribution License (https://creativecommons.org/licenses/by/4.0). Please note that the reuse, redistribution and reproduction in particular requires that the author(s) and source are credited and that individual graphics may be subject to special legal provisions.
The license is subject to the *Beilstein Journal of Organic Chemistry* terms and conditions: (https://www.beilstein-journals.org/bjoc/terms)

Table of contents

1. **EXPERIMENTAL SECTION** S1
2. **CHARACTERIZATION DATA** S4
3. **REFERENCES** S12
4. **^{1}H and ^{13}C NMR SPECTRA** S13

1. EXPERIMENTAL SECTION

General considerations

Unless noted otherwise, all reagents and solvents were purchased from commercial sources and used as received. All reactions were performed in a screw-cap sealed tubes, while the scale-up reaction was carried out in a dried round-bottomed flask (RBF). TLC analysis was performed using Merck pre-coated silica gel 60 F254 on aluminium sheets. TLC plates were visualized with UV light. The ^{1}H and ^{13}C NMR spectra were obtained in $CDCl_3$ and DMSO-d_6 as solvent using a 600 MHz, 500 MHz, 151 MHz and 125 MHz spectrometer, respectively, with tetramethylsilane (TMS) as an internal standard. Coupling constants (*J* values) are reported in Hz and chemical shifts are reported in δ values in parts per million (ppm). Multiplicity patterns were designated as s, singlet; bs, broad singlet; d, doublet; dd, doublet of doublet; dt, triplet of doublet; t, triplet; m, multiplet. ^{13}C NMR spectra were recorded with complete proton decoupling. Column chromatography was performed using silica gel (60–120 mesh).

Preparation of *N*-(arylsulfonyl)benzylamines 1a–n:

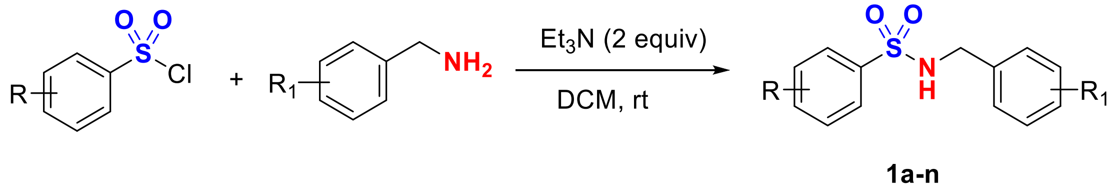

Following a modified literature protocol [1], the *N*-(arylsulfonyl)benzylamine substrates **1a–n** were synthesized by sulfonylation of the corresponding benzylamines. Initially, the substituted benzylamine derivatives (1.2 mmol) were dissolved in dry CH_2Cl_2 (5 mL). Triethylamine (2 mmol) was added and the mixture stirred at room temperature for 2 minutes. Then, substituted arylsulfonyl chlorides (1 mmol) were added dropwise to the above solution at room temperature and the resulting reaction mixture was stirred until all the substrates were consumed. Upon completion of the reaction, the mixture was diluted with H_2O (20 mL) and extracted with CH_2Cl_2 (3 × 20 mL). The combined organic layer was dried over anhydrous Na_2SO_4 and concentrated under reduced pressure. The resulting viscous concentrate was cooled and triturated with a spatula till the solid precipitated. The crude solid products obtained were washed with *n*-pentane and utilized for subsequent reactions without any further purification.

General procedure for the synthesis of *N*-arylsulfonylimines 2a–n (GP-1):

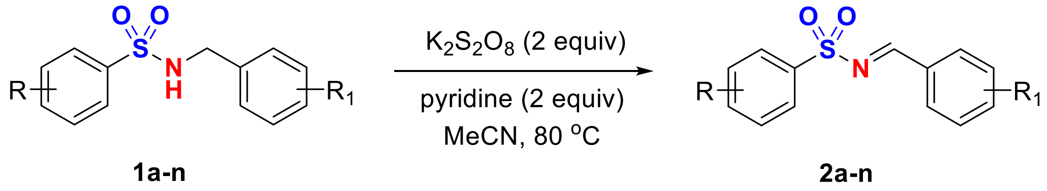

An oven-dried screw cap vial equipped with a magnetic stirring bar was charged with *N*-(arylsulfonyl)benzylamines (0.25 mmol), $K_2S_2O_8$ (2 equiv), pyridine (2 equiv) and MeCN (1 mL). The reaction mixture was heated at 80 °C and stirred for 0.5 h. Upon completion, the reaction mixture was cooled to room temperature and evaporated to dryness under reduced pressure. It was then diluted with brine (20 mL) and the aqueous phase was extracted with ethyl acetate (20 mL × 3). The combined organic phase was dried over Na_2SO_4 and concentrated under reduced pressure). The residue was purified by simple column filtration (20% EtOAc/Hex) to afford compounds **2a–n** as solid products.

General procedure for one-pot synthesis of *N*-heterocycles 4a–g (GP-2):

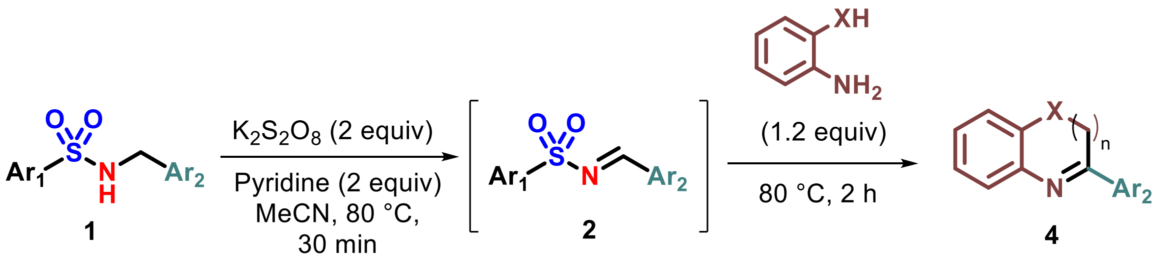

An oven-dried screw cap vial equipped with a magnetic stirring bar was charged with *N*-(arylsulfonyl)benzylamines (0.25 mmol), $K_2S_2O_8$ (2 equiv), pyridine (2 equiv) and MeCN (1 mL). The reaction mixture was heated at 80 °C and stirred for 0.5 h. Following this, $K_2S_2O_8$ (1 equiv) and the corresponding *ortho*-substituted aniline **3** (1.2 equiv) were added to the above reaction mixture and stirred at 80 °C for 2 h. Upon completion, the reaction mixture was cooled to room temperature and evaporated to dryness under reduced pressure. It was then diluted with brine (20 mL) and the aqueous phase was extracted with ethyl acetate (20 mL × 3). The combined organic phase was dried over Na_2SO_4 and concentrated under reduced pressure. The residue was purified by column chromatography (20% EtOAc/Hex) to afford compounds **4a–g** as solid products.

General procedure for gram-scale synthesis of compound 2a:

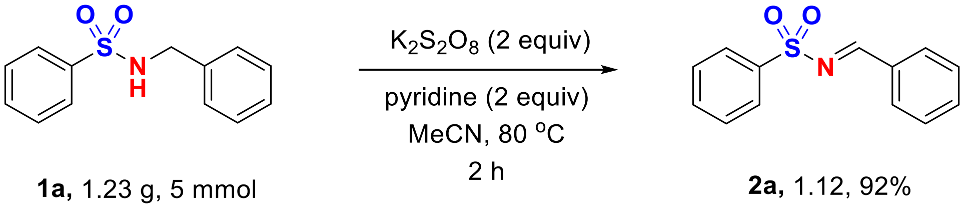

An oven-dried round-bottomed flask (50 mL) equipped with a magnetic stirring bar was charged with *N*-benzylbenzenesulfonamide (**1a**, 1.23 g, 5 mmol), $K_2S_2O_8$ (2.7 g, 10 mmol), pyridine (790 mg, 10 mmol) and MeCN (15 mL). The round-bottomed flask containing the reaction mixture was attached with a condenser and allowed to stir at 80 °C for 2 h. Upon completion, the reaction mixture was allowed to cool to room temperature and evaporated to dryness under reduced pressure. It is then diluted with brine (50 mL) and the aqueous phase was extracted with ethyl acetate (50 mL × 3). The combined organic phase was dried over Na_2SO_4, concentrated under reduced pressure, and purified by simple crystallization.

2. CHARACTERIZATION DATA

***N*-Benzylbenzenesulfonamide (1a).**[2]

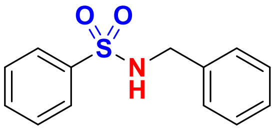

It was obtained as off white solid (239 mg, 97%). ^{1}H NMR (600 MHz, $CDCl_3$) δ 7.86 (dd, J = 8.4, 1.1 Hz, 2H), 7.59 – 7.55 (m, 1H), 7.49 (t, J = 7.7 Hz, 2H), 7.27 – 7.25 (m, 1H), 7.24 – 7.23 (m, 2H), 7.17 (dd, J = 7.7, 1.6 Hz, 2H), 4.87 (s, 1H), 4.13 (d, J = 5.9 Hz, 2H). ^{13}C NMR (151 MHz, $CDCl_3$) δ 140.0, 136.2, 132.7, 129.2, 128.8, 127.9, 127.9, 127.1, 47.3.

***N*-Benzyl-4-methylbenzenesulfonamide (1b).**[2]

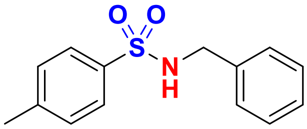

It was obtained as off white solid (250 mg, 96%). ^{1}H NMR (600 MHz, $CDCl_3$) δ 7.75 (d, J = 8.2 Hz, 2H), 7.29 (d, J = 8.1 Hz, 2H), 7.27 – 7.22 (m, 4H), 7.18 (d, J = 8.0 Hz, 2H), 4.76 (s, 1H), 4.11 (d, J = 6.2 Hz, 2H), 2.42 (s, 3H). ^{13}C NMR (151 MHz, $CDCl_3$) δ 143.6, 136.9, 136.3, 129.8, 129.7, 128.8, 128.0, 127.2, 47.3, 21.6.

***4-Methyl-N-(4-methylbenzyl)benzenesulfonamide (1c).**[2]*

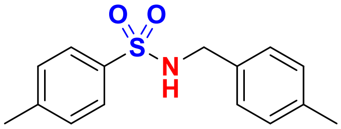

It was obtained as off white solid (261 mg, 95%). ^{1}H NMR (600 MHz, $CDCl_3$) δ 7.75 (d, J = 8.2 Hz, 2H), 7.29 (d, J = 8.1 Hz, 2H), 7.06 (s, 4H), 4.67 (s, 1H), 4.06 (d, J = 6.1 Hz, 2H), 2.43 (s, 3H), 2.29 (s, 3H). ^{13}C NMR (151 MHz, $CDCl_3$) δ 143.5, 137.7, 136.9, 133.3, 127.2, 47.1, 21.6, 21.1.

***N-(4-Methoxybenzyl)-4-methylbenzenesulfonamide (1d).**[2]*

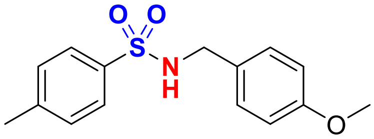

It was obtained as off white solid (283 mg, 97%).^{1}H NMR (600 MHz, $CDCl_3$) δ 7.73 (d, J = 8.1 Hz, 2H), 7.29 (d, J = 7.9 Hz, 2H), 7.09 (d, J = 8.5 Hz, 2H), 6.78 (d, J = 8.6 Hz, 2H), 4.72 (s, 1H), 4.03 (d, J = 5.9 Hz, 2H), 3.75 (s, 3H), 2.42 (s, 3H). ^{13}C NMR (151 MHz, $CDCl_3$) δ 159.3, 143.5, 137, 129.8, 129.7, 129.4, 129.2, 128.3, 127.2, 114.1, 55.3, 46.8, 21.6.

N-(2-Chlorobenzyl)-4-methylbenzenesulfonamide (1e).[2]

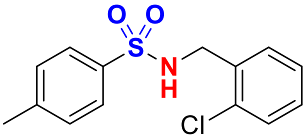

It was obtained as off white solid (281 mg, 95%).^{1}H NMR (600 MHz, $CDCl_3$) δ 7.70 (d, J = 8.3 Hz, 2H), 7.29 (dd, J = 7.1, 2.0 Hz, 1H), 7.26 (d, J = 1.7 Hz, 1H), 7.24 (d, J = 8.2 Hz, 2H), 7.16 (pd, J = 7.4, 1.7 Hz, 2H), 4.98 (s, 1H), 4.23 (d, J = 6.5 Hz, 2H), 2.39 (s, 3H). ^{13}C NMR (151 MHz, $CDCl_3$) δ 143.5, 137.0, 133.9, 133.4, 130.3, 129.7, 129.5, 129.3, 127.1, 45.2, 21.5.

N-(2-Methoxybenzyl)-4-methylbenzenesulfonamide (1f).[2]

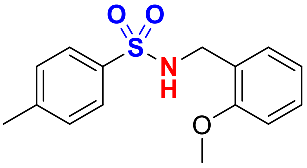

It was obtained as off white solid (279 mg, 96%).^{1}H NMR (600 MHz, $CDCl_3$) δ 7.65 (d, J = 8.1 Hz, 2H), 7.19 (d, J = 7.9 Hz, 3H), 7.05 (d, J = 7.1 Hz, 1H), 6.79 (t, J = 7.4 Hz, 1H), 6.72 (d, J = 8.2 Hz, 1H), 5.13 (s, 1H), 4.12 (s, 2H), 3.72 (s, 3H), 2.37 (s, 3H). ^{13}C NMR (151 MHz, $CDCl_3$) δ 157.2, 143.1, 137.3, 129.8, 129.4, 129.3, 127.1, 124.3, 120.6, 110.1, 110.1, 55.2, 44.0, 21.5.

N-Benzyl-4-bromobenzenesulfonamide (1g).[2]

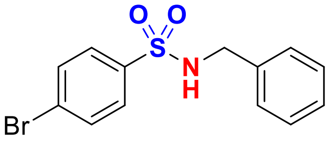

It was obtained as off white solid (301 mg, 93%).^{1}H NMR (600 MHz, $CDCl_3$) δ 7.69 (d, J = 8.6 Hz, 2H), 7.61 (d, J = 8.6 Hz, 2H), 7.26 (d, J = 6.6 Hz, 2H), 7.17 (d, J = 7.6 Hz, 2H), 4.90 (s, 1H), 4.13 (s, 2H). ^{13}C NMR (151 MHz, $CDCl_3$) δ 139.1, 135.9, 132.4, 128.8, 128.7, 128.1, 127.9, 127.7, 47.3.

N-Benzyl-[1,1'-biphenyl]-4-sulfonamide (1h).[3]

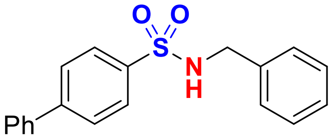

It was obtained as off white solid (298 mg, 92%).^{1}H NMR (600 MHz, $CDCl_3$) δ 7.92 (d, J = 8.5 Hz, 2H), 7.70 (d, J = 8.4 Hz, 2H), 7.60 (dd, J = 8.2, 1.1 Hz, 2H), 7.48 (t, J = 7.5 Hz, 2H), 7.44 – 7.40 (m, 1H), 7.28 – 7.25 (m, 2H), 7.24 (s, 1H), 7.20 (d, J = 6.2 Hz, 2H), 4.79 (t, J = 6.1 Hz, 1H), 4.18 (d, J = 6.2 Hz, 2H). ^{13}C NMR (151 MHz, $CDCl_3$) δ 145.7, 139.4, 138.5, 136.2, 129.1, 128.9, 127.8, 127.7, 127.4, 47.4.

***N*-Benzyl-3-chloro-4-fluorobenzenesulfonamide (1i).**[3]

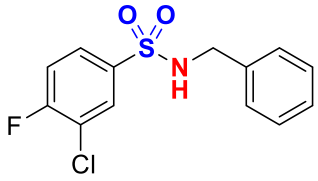

It was obtained as off white solid (269 mg, 89%). ^{1}H NMR (600 MHz, $CDCl_3$) δ 7.86 (dd, J = 6.7, 2.3 Hz, 1H), 7.70 (ddd, J = 8.6, 4.3, 2.3 Hz, 1H), 7.28 – 7.24 (m, 3H), 7.21 (t, J = 8.5 Hz, 1H), 7.17 (dd, J = 7.5, 1.9 Hz, 2H), 4.99 (s, 1H), 4.17 (d, J = 5.6 Hz, 2H). ^{13}C NMR (151 MHz, $CDCl_3$) δ 161.4, 159.7, 137.3, 135.7, 130.2, 128.8, 128.2, 128.0, 127.9, 127.6, 122.4, 122.3, 117.5, 117.3, 47.4.

***N*-Benzylnaphthalene-1-sulfonamide (1j).**[4]

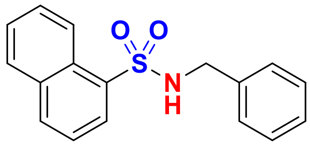

It was obtained as off white solid (277 mg, 93%). ^{1}H NMR (600 MHz, $CDCl_3$) δ 8.64 (d, J = 8.6 Hz, 1H), 8.26 (d, J = 7.3 Hz, 1H), 8.06 (d, J = 8.2 Hz, 1H), 7.94 (d, J = 8.1 Hz, 1H), 7.65 (t, J = 7.7 Hz, 1H), 7.60 (t, J = 7.5 Hz, 1H), 7.51 (t, J = 7.8 Hz, 1H), 7.16 – 7.14 (m, 3H), 7.04 (dd, J = 6.5, 2.6 Hz, 2H), 4.99 (s, 1H), 4.07 (d, J = 6.1 Hz, 2H). ^{13}C NMR (151 MHz, $CDCl_3$) δ 136.1, 134.5, 134.3, 130.1, 129.9, 129.2, 128.5, 128.2, 127.9, 127.7, 127.0, 124.4, 124.3, 124.2, 47.4.

***N*-Benzylnaphthalene-2-sulfonamide (1k).**[4]

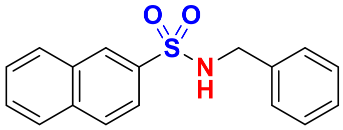

It was obtained as off white solid (278 mg, 93%). ^{1}H NMR (600 MHz, $CDCl_3$) δ 8.43 (s, 1H), 7.95 (d, J = 8.7 Hz, 2H), 7.91 (d, J = 8.1 Hz, 1H), 7.83 (dd, J = 8.6, 1.9 Hz, 1H), 7.65 (ddd, J = 8.2, 7.0, 1.3 Hz, 1H), 7.61 (t, J = 8.1 Hz, 1H), 7.24 – 7.19 (m, 3H), 7.18 (d, J = 8.0 Hz, 2H), 4.91 (s, 1H), 4.16 (d, J = 6.1 Hz, 2H). ^{13}C NMR (151 MHz, $CDCl_3$) δ 136.7, 136.2, 134.9, 132.2, 129.6, 129.3, 128.9, 128.7, 128.0, 127.6, 122.3, 47.4.

***N*-Benzylmethanesulfonamide (1l).**[5]

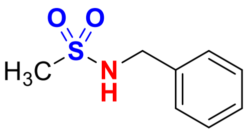

It was obtained as off white solid (170 mg, 92%). ^{1}H NMR (600 MHz, $CDCl_3$) δ 7.37 – 7.28 (m, 5H), 5.01 (s, 1H), 4.28 (s, 2H), 2.82 (d, J = 3.3 Hz, 3H). ^{13}C NMR (151 MHz, $CDCl_3$) δ 136.8, 128.9, 128.1, 127.9, 47.2, 41.0.

***N*-Benzhydryl-4-methylbenzenesulfonamide (1m).**[2]

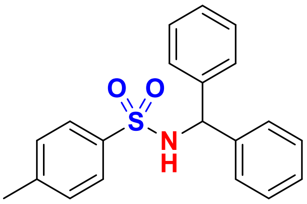

It was obtained as off white solid (298 mg, 88%).^{1}H NMR (600 MHz, $CDCl_3$) δ 7.55 (d, J = 8.3 Hz, 2H), 7.21 – 7.17 (m, 6H), 7.12 (d, J = 8.4 Hz, 2H), 7.10 – 7.08 (m, 4H), 5.55 (d, J = 7.2 Hz, 1H), 5.23 (t, J = 15.4 Hz, 1H), 2.36 (s, 3H). ^{13}C NMR (151 MHz, $CDCl_3$) δ 143.2, 140.5, 137.3, 129.4, 128.6, 127.6, 127.4, 127.2, 61.4, 21.5.

***(S)*-4-Methyl-*N*-(1-phenylethyl)benzenesulfonamide (1n).**[2]

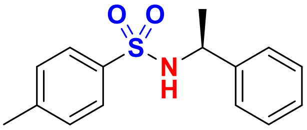

It was obtained as off white solid (257 mg, 93%).^{1}H NMR (600 MHz, $CDCl_3$) δ 7.60 (d, J = 8.3 Hz, 2H), 7.20 – 7.15 (m, 5H), 7.08 (dd, J = 7.5, 1.8 Hz, 2H), 4.99 (s, 1H), 4.44 (p, J = 6.9 Hz, 1H), 2.37 (s, 3H), 1.41 (d, J = 6.9 Hz, 3H). ^{13}C NMR (151 MHz, $CDCl_3$) δ 143.2, 142.1, 137.6, 129.5, 128.6, 127.5, 127.1, 126.1, 53.7, 23.6, 21.5.

***(E)*-*N*-Benzylidenebenzenesulfonamide (2a).**[5]

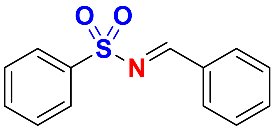

It was obtained as white solid (59 mg, 96% using **GP-1**).^{1}H NMR (500 MHz, $CDCl_3$) δ 9.06 (s, 1H), 8.01 (d, J = 8.4 Hz, 2H), 7.93 (d, J = 7.2 Hz, 2H), 7.62 (dt, J = 7.4, 5.4 Hz, 2H), 7.55 (t, J = 7.7 Hz, 2H), 7.49 (t, J = 7.6 Hz, 2H). ^{13}C NMR (126 MHz, $CDCl_3$) δ 170.7, 138.2, 135.1, 133.6, 132.3, 131.5, 129.2, 128.1.

***(E)*-*N*-Benzylidene-4-methylbenzenesulfonamide (2b).**[5]

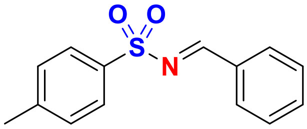

It was obtained as white solid (61 mg, 94% using **GP-1**).^{1}H NMR (600 MHz, $CDCl_3$) δ 9.02 (s, 1H), 7.92 (dd, J = 8.2, 1.2 Hz, 2H), 7.88 (d, J = 8.4 Hz, 2H), 7.61 (tt, J = 7.9, 1.3 Hz, 1H), 7.48 (t, J = 7.8 Hz, 2H), 7.34 (dd, J = 8.5, 0.6 Hz, 2H), 2.43 (s, 3H). ^{13}C NMR (151 MHz, $CDCl_3$) δ 170.2, 144.7, 135.1, 135.0, 132.4, 131.4, 129.9, 129.2, 128.1, 21.7.

(E)-4-Methyl-N-(4-methylbenzylidene)benzenesulfonamide (2c).[5]

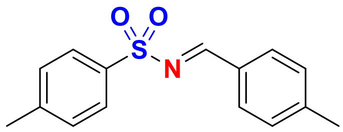

It was obtained as white solid (64 mg, 95% using **GP-1**).^{1}H NMR (600 MHz, $CDCl_3$) δ 8.97 (s, 1H), 7.87 (d, J = 8.4 Hz, 2H), 7.80 (d, J = 8.1 Hz, 2H), 7.33 (dd, J = 8.5, 0.6 Hz, 2H), 7.28 (d, J = 8.1 Hz, 2H), 2.42 (s, 3H), 2.42 (s, 3H). ^{13}C NMR (151 MHz, $CDCl_3$) δ 170.0, 146.5, 144.5, 135.4, 131.5, 130.0, 129.9, 129.8, 128.1, 22.1, 21.7.

(E)-N-(4-Methoxybenzylidene)-4-methylbenzenesulfonamide (2d).[5]

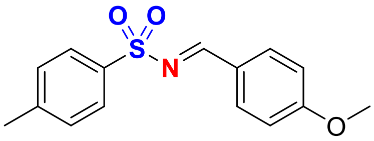

It was obtained as white solid (67 mg, 92% using **GP-1**).^{1}H NMR (600 MHz, $CDCl_3$) δ 8.93 (s, 1H), 7.87 (d, J = 6.2 Hz, 2H), 7.86 (d, J = 5.6 Hz, 2H), 7.32 (dd, J = 8.6, 0.6 Hz, 2H), 6.95 (d, J = 8.9 Hz, 2H), 3.87 (s, 3H), 2.42 (s, 3H). ^{13}C NMR (151 MHz, $CDCl_3$) δ 169.3, 165.3, 144.3, 135.7, 133.8, 129.8, 127.9, 125.2, 114.7, 55.7, 21.7.

(E)-N-(2-Chlorobenzylidene)-4-methylbenzenesulfonamide (2e).[5]

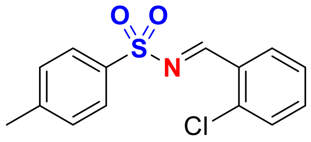

It was obtained as white solid (59 mg, 80% using **GP-1**).^{1}H NMR (600 MHz, $CDCl_3$) δ 9.49 (s, 1H), 8.14 (dd, J = 7.9, 1.7 Hz, 1H), 7.89 (d, J = 8.3 Hz, 2H), 7.51 (ddd, J = 8.0, 7.3, 1.7 Hz, 1H), 7.45 (dd, J = 8.1, 1.1 Hz, 1H), 7.35 (d, J = 8.0 Hz, 2H), 7.33 (t, J = 7.2 Hz, 1H), 2.43 (s, 3H). ^{13}C NMR (151 MHz, $CDCl_3$) δ 166.8, 144.9, 139.0, 135.7, 134.7, 130.5, 130.2, 129.9, 129.8, 128.3, 127.44, 21.7.

(E)-N-(2-Methoxybenzylidene)-4-methylbenzenesulfonamide (2f).[6]

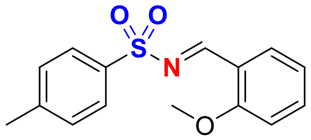

It was obtained as white solid (60 mg, 82% using **GP-1**).^{1}H NMR (600 MHz, $CDCl_3$) δ 9.53 (s, 1H), 8.04 (dd, J = 7.9, 1.7 Hz, 1H), 7.87 (d, J = 8.3 Hz, 2H), 7.54 (ddd, J = 8.9, 7.4, 1.8 Hz, 1H), 7.31 (d, J = 8.0 Hz, 2H), 6.96 (t, J = 7.6 Hz, 1H), 6.94 (d, J = 8.5 Hz, 1H), 3.90 (s, 3H), 2.41 (s, 3H). ^{13}C NMR (151 MHz, $CDCl_3$) δ 166.4, 161.7, 144.3, 137.0, 135.7, 129.7, 129.4, 128.0, 120.9, 111.5, 55.8, 21.7.

***(E)*-N-Benzylidene-4-bromobenzenesulfonamide (2g).**[5]

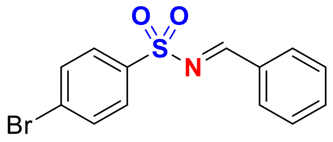

It was obtained as white solid (65 mg, 80% using **GP-1**).^{1}H NMR (600 MHz, $CDCl_3$) δ 9.05 (s, 1H), 7.92 (d, J = 7.2 Hz, 2H), 7.86 (d, J = 8.6 Hz, 2H), 7.68 (d, J = 8.6 Hz, 2H), 7.63 (t, J = 7.5 Hz, 1H), 7.50 (t, J = 7.7 Hz, 2H). ^{13}C NMR (151 MHz, $CDCl_3$) δ 171.1, 137.3, 135.4, 132.5, 132.2, 131.5, 129.6, 129.3, 128.9.

***(E)*-N-Benzylidene-[1,1'-biphenyl]-4-sulfonamide (2h).**[5]

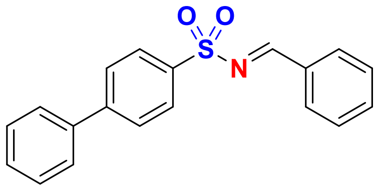

It was obtained as white solid (68 mg, 84% using **GP-1**).^{1}H NMR (600 MHz, $CDCl_3$) δ 9.08 (s, 1H), 8.06 (d, J = 8.5 Hz, 2H), 7.95 (d, J = 7.1 Hz, 2H), 7.74 (d, J = 8.5 Hz, 2H), 7.62 (t, J = 7.4 Hz, 1H), 7.59 (d, J = 7.3 Hz, 2H), 7.51 – 7.45 (m, 4H), 7.41 (t, J = 7.4 Hz, 1H). ^{13}C NMR (151 MHz, $CDCl_3$) δ 170.6, 146.6, 139.3, 136.7, 135.1, 132.4, 131.5, 129.2, 129.1, 128.7, 128.6, 127.9, 127.4.

***(E)*-N-Benzylidene-3-chloro-4-fluorobenzenesulfonamide (2i).**[6]

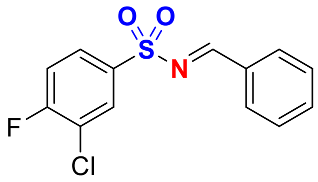

It was obtained as white solid (58 mg, 78% using **GP-1**).^{1}H NMR (600 MHz, $CDCl_3$) δ 9.06 (s, 1H), 8.08 (dd, J = 6.7, 2.3 Hz, 1H), 7.94 (dd, J = 8.3, 1.2 Hz, 2H), 7.92 – 7.90 (m, 1H), 7.65 (t, J = 7.5 Hz, 1H), 7.51 (t, J = 7.8 Hz, 2H), 7.30 (t, J = 8.5 Hz, 1H). ^{13}C NMR (151 MHz, $CDCl_3$) δ 171.4, 162.1, 135.5, 132.1, 131.6, 131.1, 129.3, 128.7, 128.6, 117.6, 117.5.

***(E)*-N-Benzylidenenaphthalene-1-sulfonamide (2j).**[7]

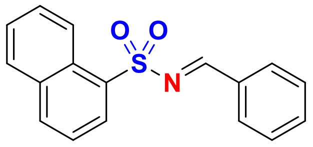

It was obtained as white solid (61 mg, 82% using **GP-1**).^{1}H NMR (600 MHz, $CDCl_3$) δ 9.15 (s, 1H), 8.86 (d, J = 8.2 Hz, 1H), 8.39 (dd, J = 7.4, 1.2 Hz, 1H), 8.11 (d, J = 8.2 Hz, 1H), 7.93 (d, J = 8.1 Hz, 1H), 7.90 (dd, J = 8.2, 1.2 Hz, 2H), 7.71 (ddd, J = 8.5, 6.9, 1.3 Hz, 1H), 7.62 – 7.56 (m, 3H), 7.45 (t, J = 7.8 Hz, 2H). ^{13}C NMR (151 MHz, $CDCl_3$) δ 170.7, 135.3, 135.1, 134.2, 133.7, 132.4, 131.4, 129.6, 129.2, 129.1, 128.9, 128.4, 127.0, 125.5, 124.3.

(E)-N-Benzylidenenaphthalene-2-sulfonamide (2k).[5]

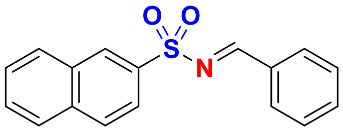

It was obtained as white solid (62 mg, 84%using **GP-1**).^{1}H NMR (600 MHz, $CDCl_3$) δ 9.10 (s, 1H), 8.60 (d, J = 1.2 Hz, 1H), 7.99 (dd, J = 8.5, 3.0 Hz, 2H), 7.96 – 7.92 (m, 3H), 7.91 (d, J = 8.1 Hz, 1H), 7.65 (ddd, J = 8.2, 6.9, 1.3 Hz, 1H), 7.63 – 7.59 (m, 2H), 7.48 (t, J = 7.8 Hz, 2H). ^{13}C NMR (151 MHz, $CDCl_3$) δ 170.6, 135.3, 135.1, 135.0, 132.4, 132.2, 131.4, 129.7, 129.5, 129.5, 129.3, 129.2, 128.0, 127.7, 123.0.

(E)-N-Benzylidenemethanesulfonamide (2l).[5]

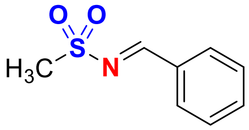

It was obtained as white solid (33 mg, 90% using **GP-1**). ^{1}H NMR (600 MHz, $CDCl_3$) δ 9.02 (s, 1H), 7.95 (d, J = 7.2 Hz, 2H), 7.65 (t, J = 7.5 Hz, 1H), 7.52 (t, J = 7.8 Hz, 2H), 3.13 (s, 3H). ^{13}C NMR (151 MHz, $CDCl_3$) δ 171.8, 135.3, 132.1, 131.4, 129.3, 40.3.

2-Phenylquinazolin-4(3H)-one (4a).[8]

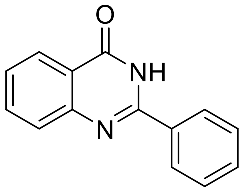

It was obtained as white solid (48 mg, 86% using **GP-2**).^{1}H NMR (400 MHz, $CDCl_3$) δ 11.69 (s, 1H), 8.36 (d, J = 7.9 Hz, 1H), 8.29 (dd, J = 6.3, 2.8 Hz, 2H), 7.85 (dt, J = 16.0, 8.0 Hz, 2H), 7.64 – 7.60 (m, 3H), 7.53 (t, J = 6.8 Hz, 1H). ^{13}C NMR (101 MHz, $CDCl_3$) δ 151.7, 149.5, 134.9, 132.8, 131.6, 129.0, 128.0, 127.4, 126.8, 126.3, 120.8.

2-(p-Tolyl)quinazolin-4(3H)-one (4b).[8]

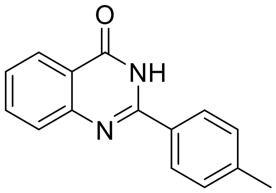

It was obtained as white solid (50 mg, 85% using **GP-2**).^{1}H NMR (600 MHz, $CDCl_3$) δ 11.27 (s, 1H), 8.32 (dd, J = 7.9, 1.0 Hz, 1H), 8.12 (d, J = 8.2 Hz, 2H), 7.85 – 7.74 (m, 2H), 7.49 (t, J = 8.0 Hz, 1H), 7.37 (d, J = 8.0 Hz, 2H), 2.45 (s, 3H). ^{13}C NMR (151 MHz, $CDCl_3$) δ 163.7, 151.8, 149.7, 142.3, 134.9, 130.0, 129.9, 128.0, 127.3, 126.6, 126.4, 120.9, 21.6.

2-Phenylquinazoline (4c).[9]

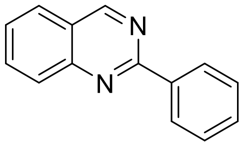

It was obtained as white solid (43 mg, 84% using **GP-2**).^{1}H NMR (500 MHz, $CDCl_3$) δ 9.47 (s, 1H), 8.61 (dd, J = 8.1, 1.5 Hz, 2H), 8.09 (d, J = 8.3 Hz, 1H), 7.91 (ddd, J = 8.3, 7.5, 4.1 Hz, 2H), 7.61 (t, J = 7.9 Hz, 1H), 7.56 – 7.50 (m, 3H). ^{13}C NMR (126 MHz, $CDCl_3$) δ 161.1, 160.6, 150.8, 138.1, 134.2, 130.7, 128.7, 128.6, 127.3, 127.2, 123.7.

2-(p-Tolyl)quinazoline (4d).[9]

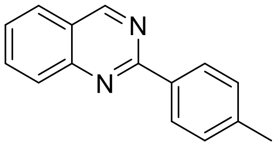

It was obtained as white solid (46 mg, 83% using **GP-2**).^{1}H NMR (400 MHz, $CDCl_3$) δ 9.44 (s, 1H), 8.50 (d, *J* = 8.1 Hz, 2H), 8.06 (d, *J* = 8.4 Hz, 1H), 7.93 – 7.84 (m, 2H), 7.59 (t, *J* = 7.5 Hz, 1H), 7.34 (d, *J* = 8.0 Hz, 2H), 2.44 (s, 3H). ^{13}C NMR (101 MHz, $CDCl_3$) δ 161.1, 160.4, 150.8, 140.8, 135.3, 134.0, 129.4, 128.5, 128.5, 127.1, 127.0, 123.5, 21.5.

2-Phenylbenzo[d]thiazole (4e).[9]

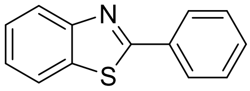

It was obtained as white solid (45 mg, 85% using **GP-2**).^{1}H NMR (600 MHz, $CDCl_3$) δ 8.11 – 8.06 (m, 3H), 7.90 (d, *J* = 8.0 Hz, 1H), 7.52 – 7.46 (m, 4H), 7.38 (t, *J* = 7.3 Hz, 1H). ^{13}C NMR (151 MHz, $CDCl_3$) δ 168.1, 154.2, 135.1, 133.7, 131.0, 129.1, 127.6, 126.4, 125.2, 123.3, 121.7.

2-Phenyl-1H-benzo[d]imidazole (4f).[8]

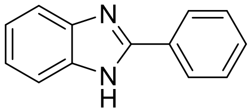

It was obtained as white solid (17 mg, 35% using **GP-2**).^{1}H NMR (400 MHz, DMSO-d_6) δ 12.59 (s, 1H), 8.31 (d, *J* = 6.7 Hz, 2H), 7.85 (d, *J* = 7.9 Hz, 1H), 7.61 – 7.47 (m, 4H), 7.34 (t, *J* = 8.1 Hz, 2H). ^{13}C NMR (101 MHz, DMSO- d_6) δ 155.0, 154.6, 136.0, 132.5, 130.7, 130.6, 129.6, 129.2, 128.3, 123.8, 115.5.

3. REFERENCES

1. Laha, J. K.; Dayal, N.; Jain, R.; Patel, K. *J. Org. Chem.*, **2014**, *79*, 10899–10907.
2. Cui, X.; Shi, F.; Tse, M. K.; Goerdes, D.; Thurow, K.; Beller, M.; Deng, Y. *Adv. Synth. Catal.*, **2009**, *351*, 2949–2958.
3. Tauro, M.; Laghezza, A.; Loiodice, F.; Piemontese, L.; Caradonna, A.; Capelli, D.; Montanari, R.; Pochetti, G.; Di Pizio, A.; Agamennone, M. *J. Enzyme Inhib. Med. Chem.*, **2016**, *31*, 25–37.
4. Reed-Berendt, B. G.; Morrill, L. C. *J. Org. Chem.*, **2019**, *84*, 3715–3724.
5. Wang, J.; Yi, W. J. *Molecules* **2019**, *24*, 3771.
6. Wen, H.; Luo, N.; Zhu, Q.; Luo, R. *J. Org. Chem.*, **2021**, *86*, 3850–3859.
7. Dong, D. J.; Li, H. H.; Tian, S. K. *J. Am. Chem. Soc.*, **2010**, *132*, 5018–5020.
8. Laha, J. K.; Hunjan, M. K. *J. Org. Chem.*, **2022**, *87*, 2315–2323.
9. Laha, J. K.; Panday, S.; Tomar, M.; Patel, K. V. *Org. Biomol. Chem.*, **2021**, *19*, 845–853.

4. ^{1}H and ^{13}C NMR SPECTRA

1a ^{1}H NMR (600 MHz, $CDCl_3$)

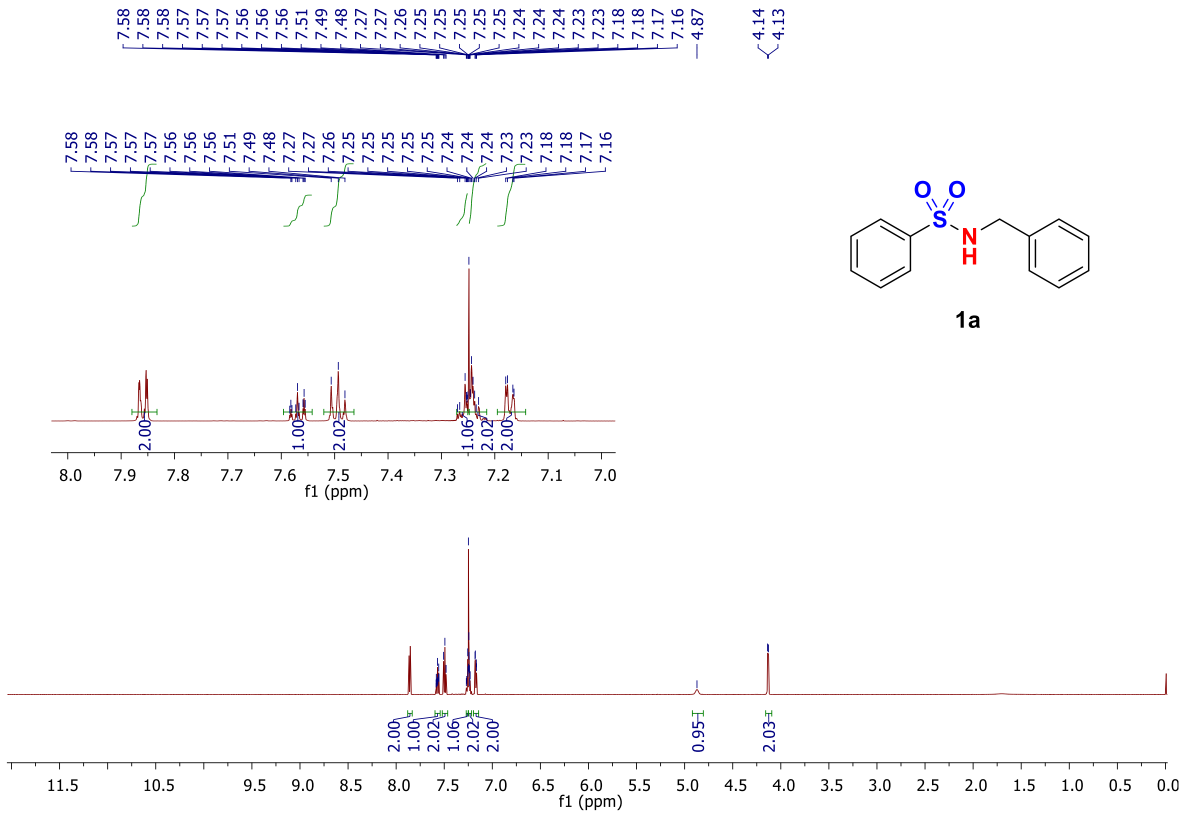

1a

^{13}C{^{1}H} NMR (151 MHz, $CDCl_3$)

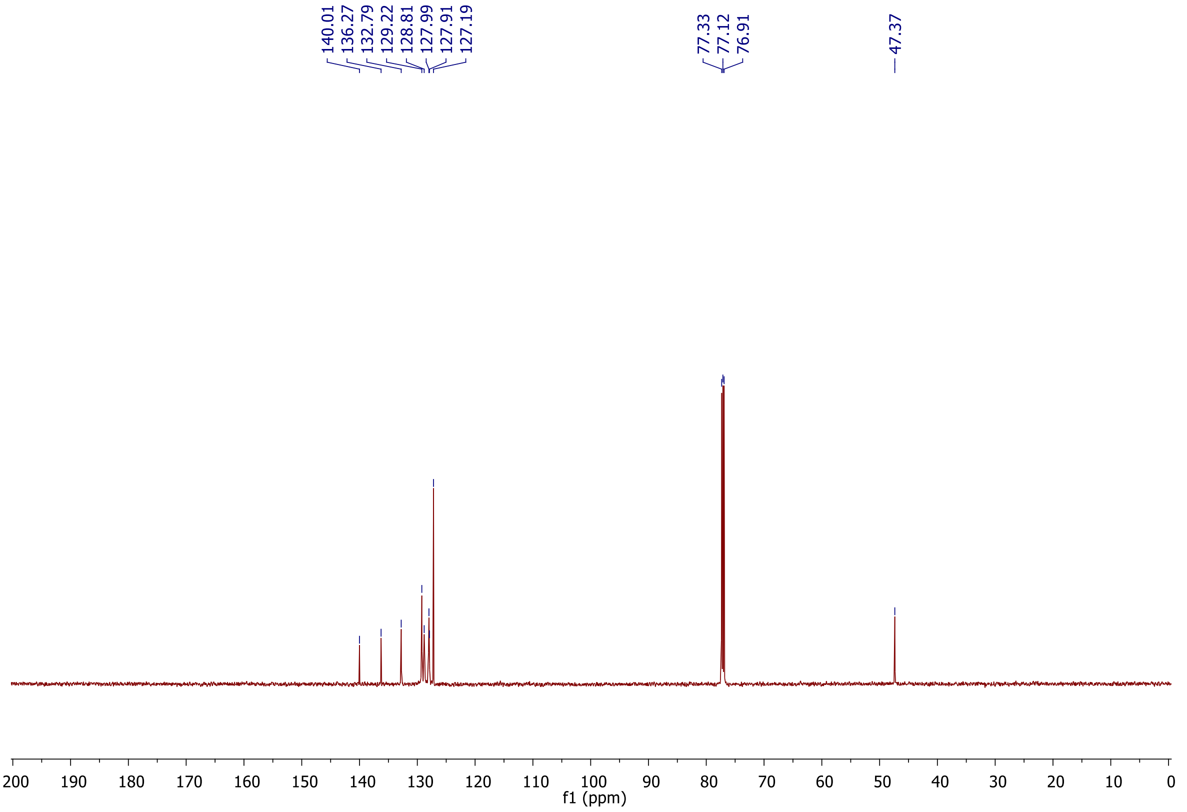

1b ^{1}H NMR (600 MHz, $CDCl_3$)

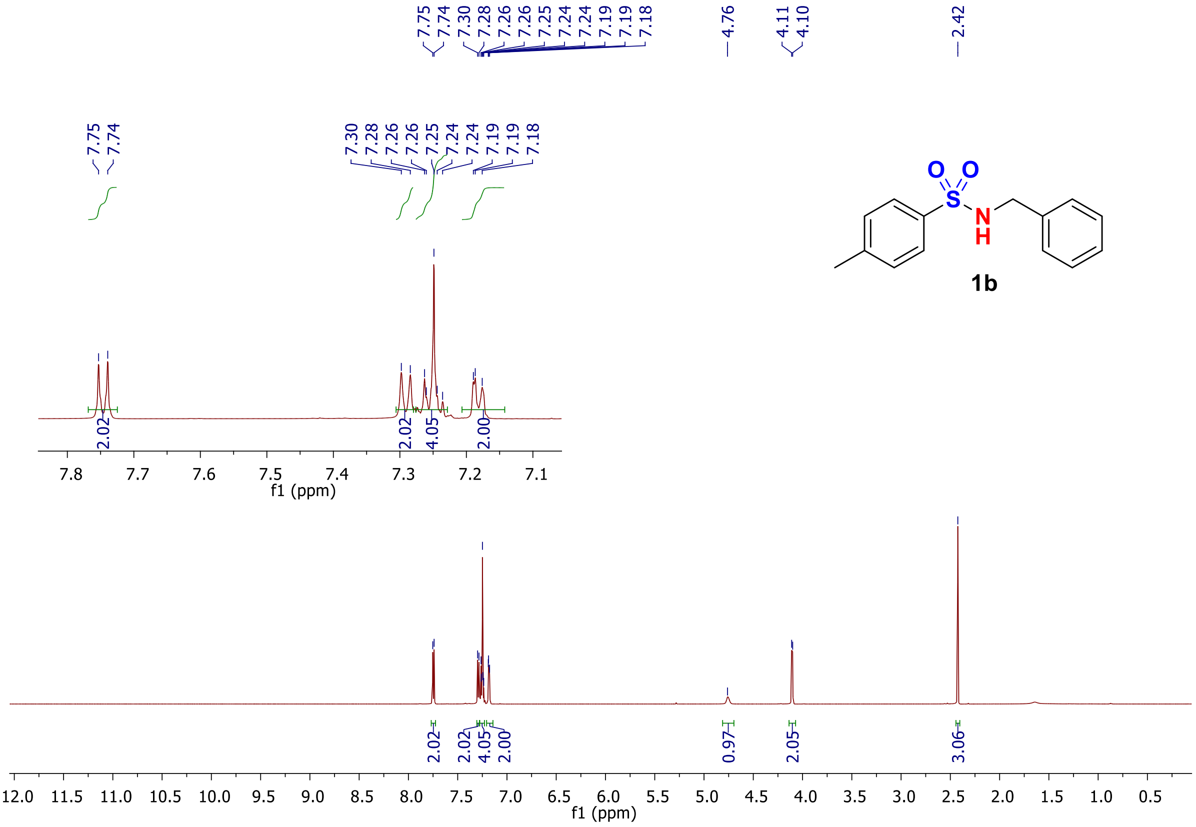

^{13}C{^{1}H} NMR (151 MHz, $CDCl_3$)

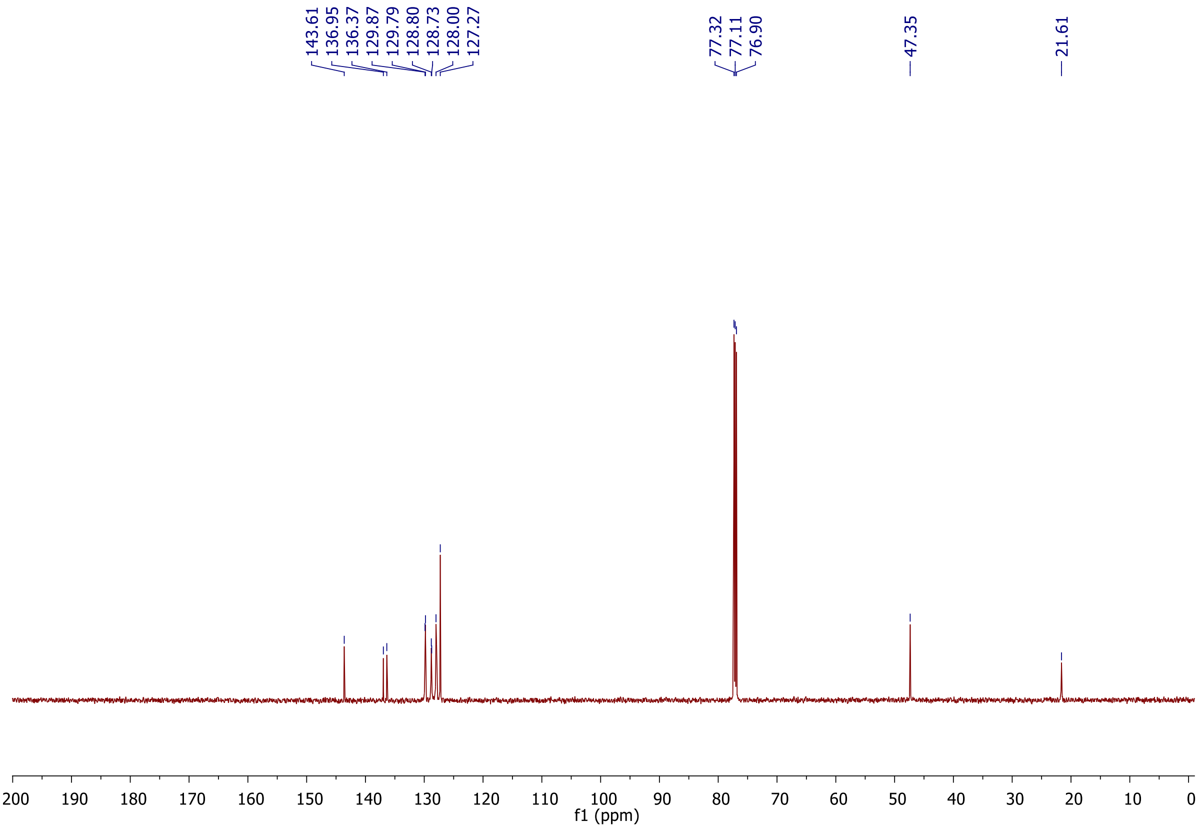

1c ^{1}H NMR (600 MHz, $CDCl_3$)

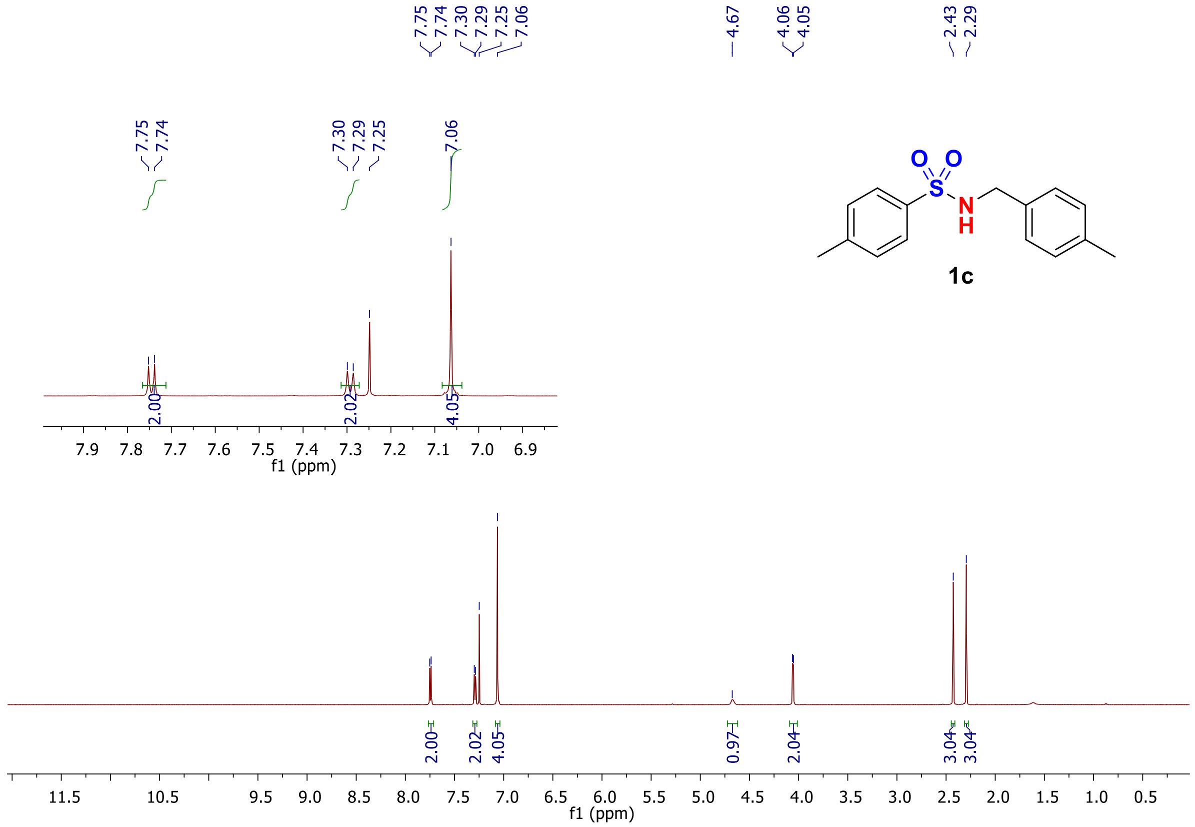

^{13}C{^{1}H} NMR (151 MHz, $CDCl_3$)

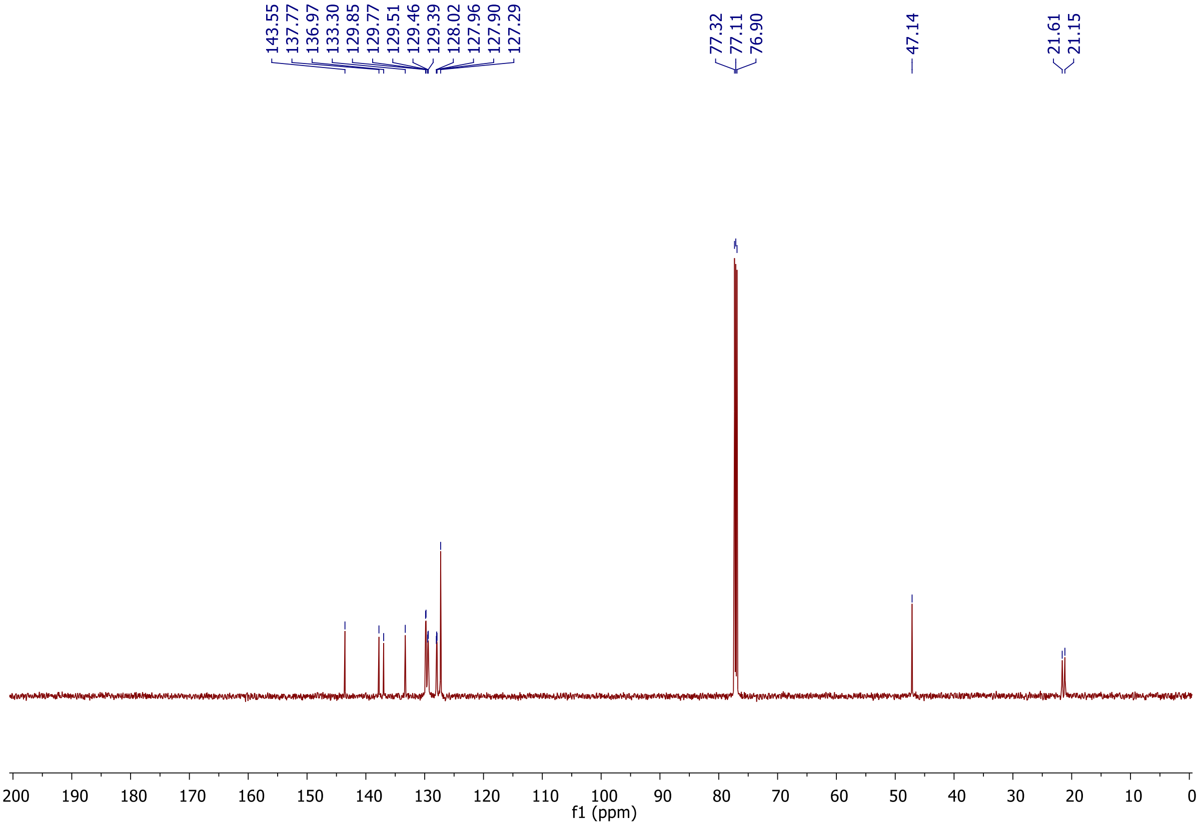

1d ^{1}H NMR (600 MHz, $CDCl_3$)

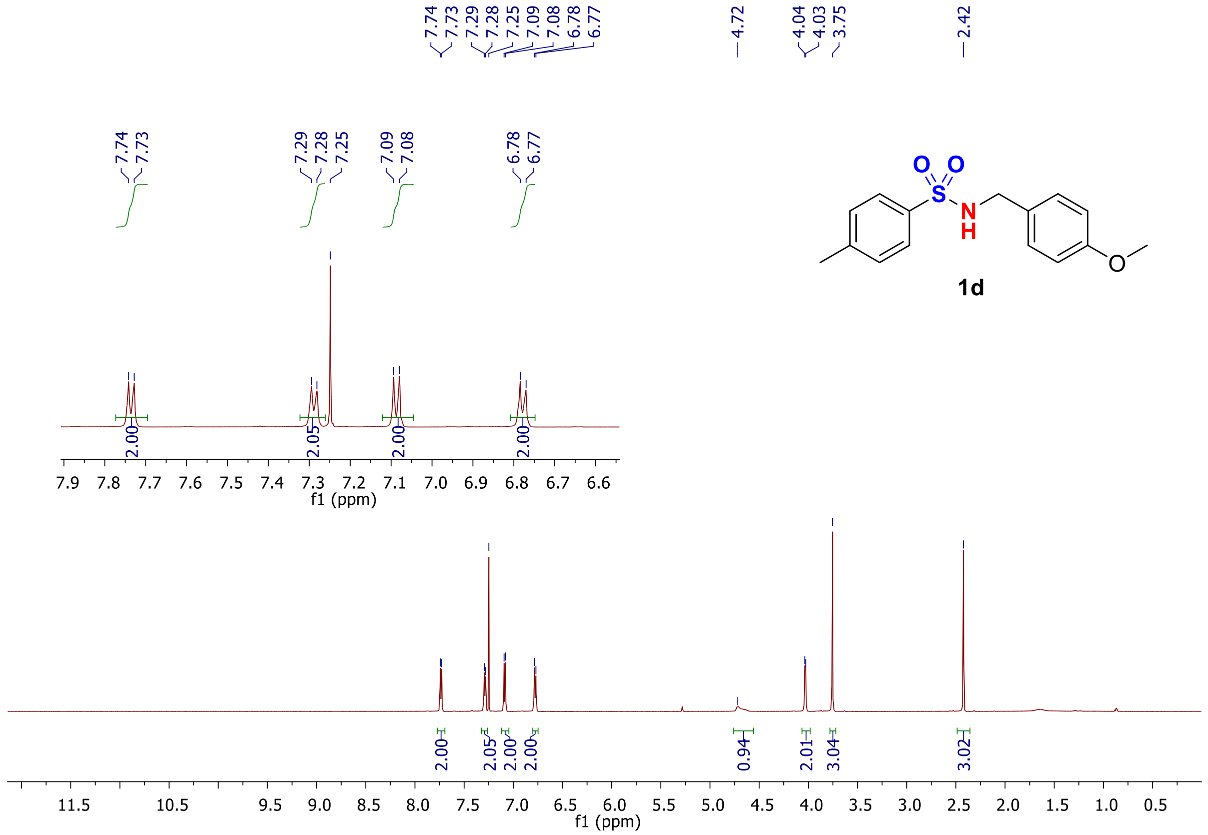

^{13}C{^{1}H} NMR (151 MHz, $CDCl_3$)

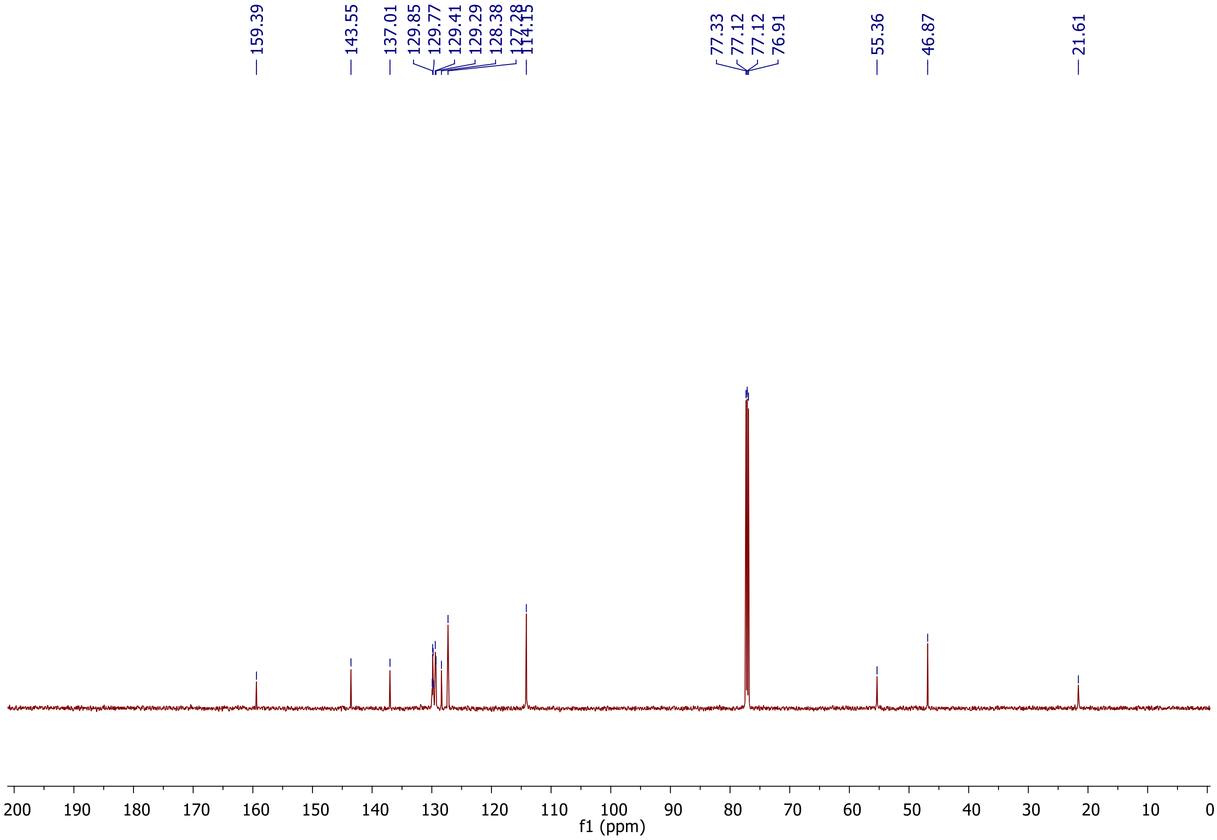

1e ^{1}H NMR (600 MHz, $CDCl_3$)

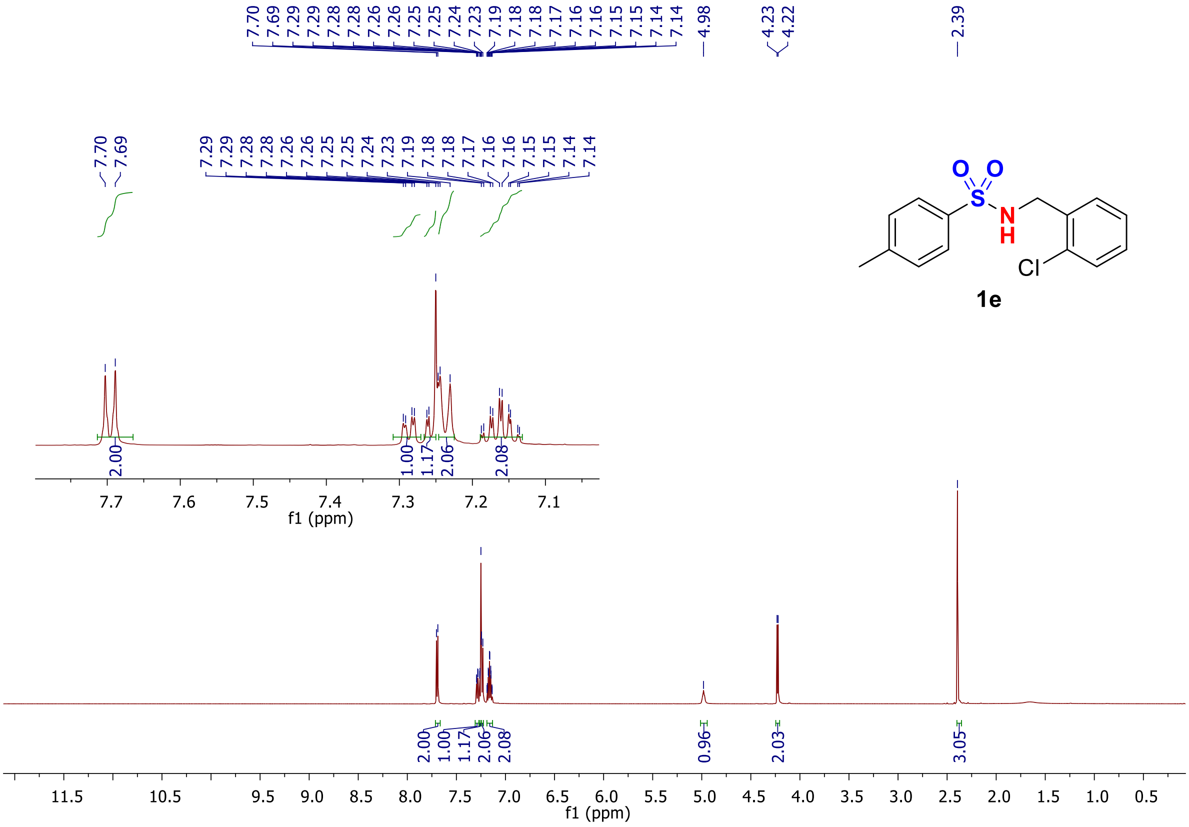

^{13}C{^{1}H} NMR (151 MHz, $CDCl_3$)

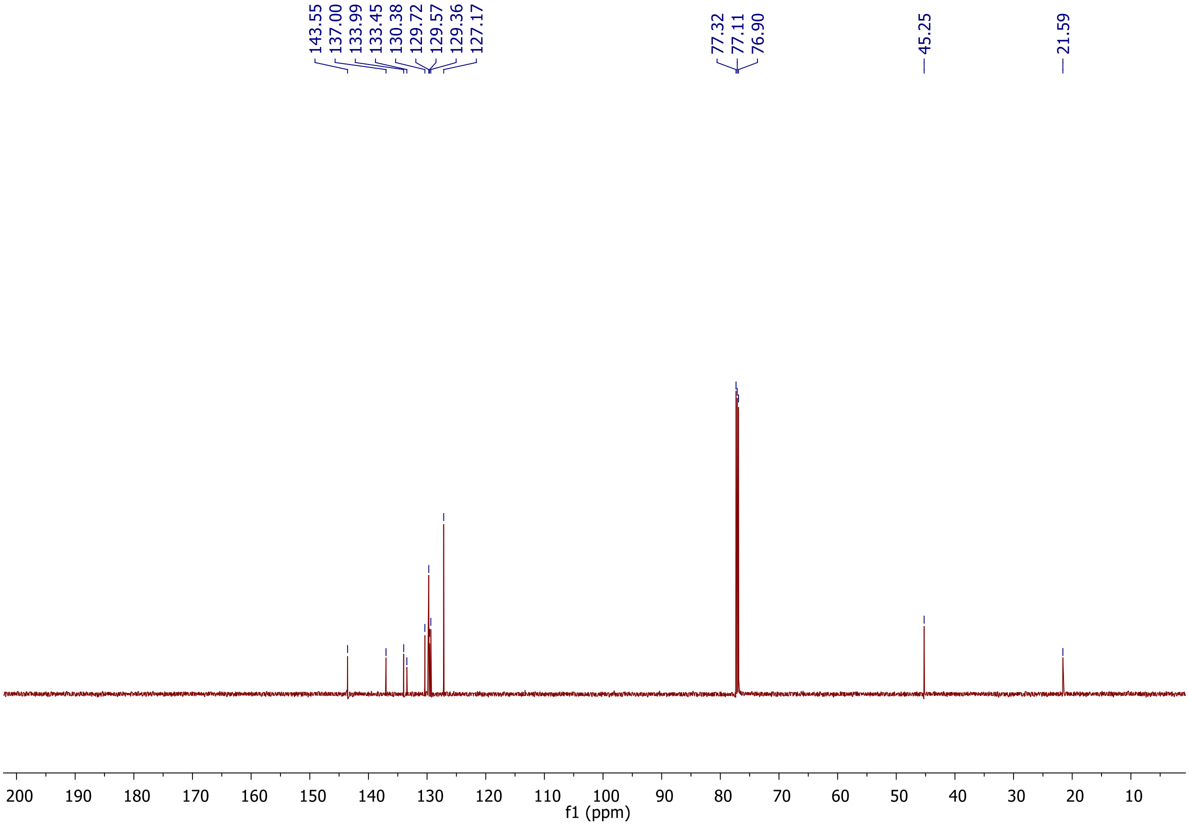

1f ^{1}H NMR (600 MHz, $CDCl_3$)

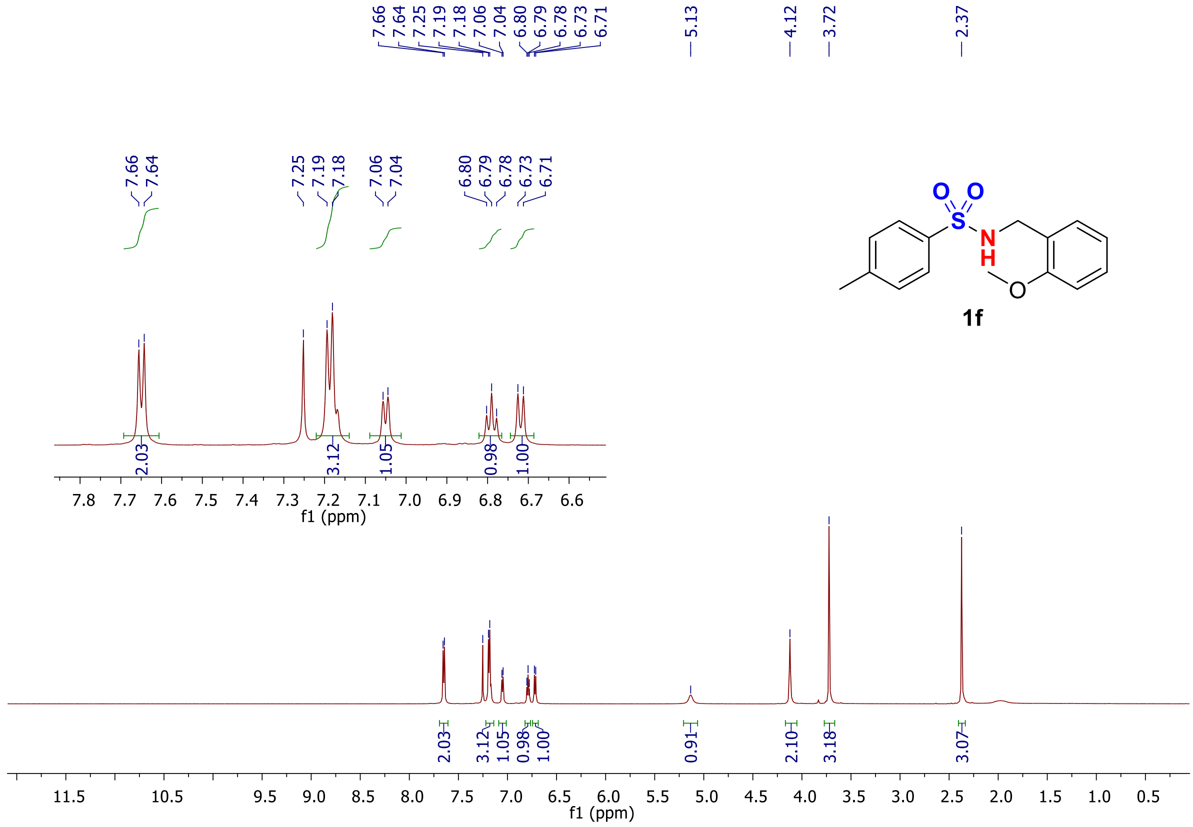

1f

^{13}C{^{1}H} NMR (151 MHz, $CDCl_3$)

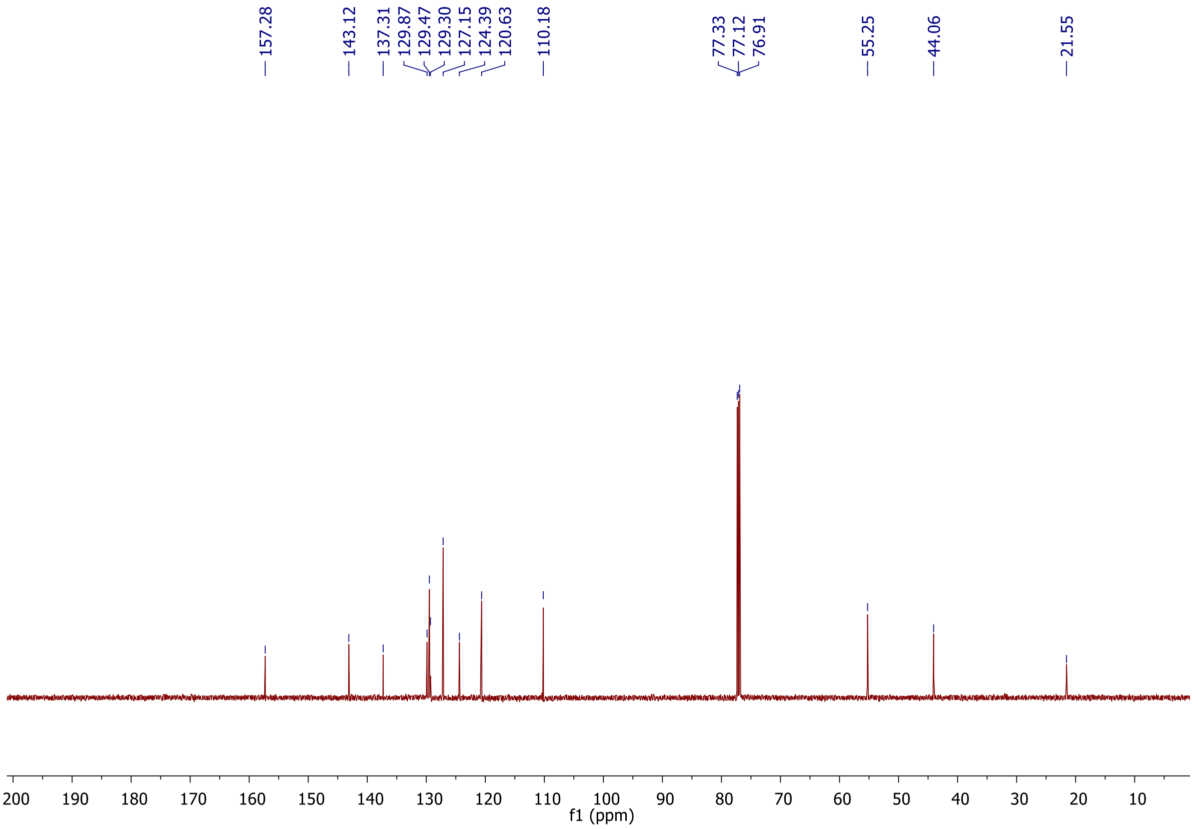

1g ^{1}H NMR (600 MHz, $CDCl_3$)

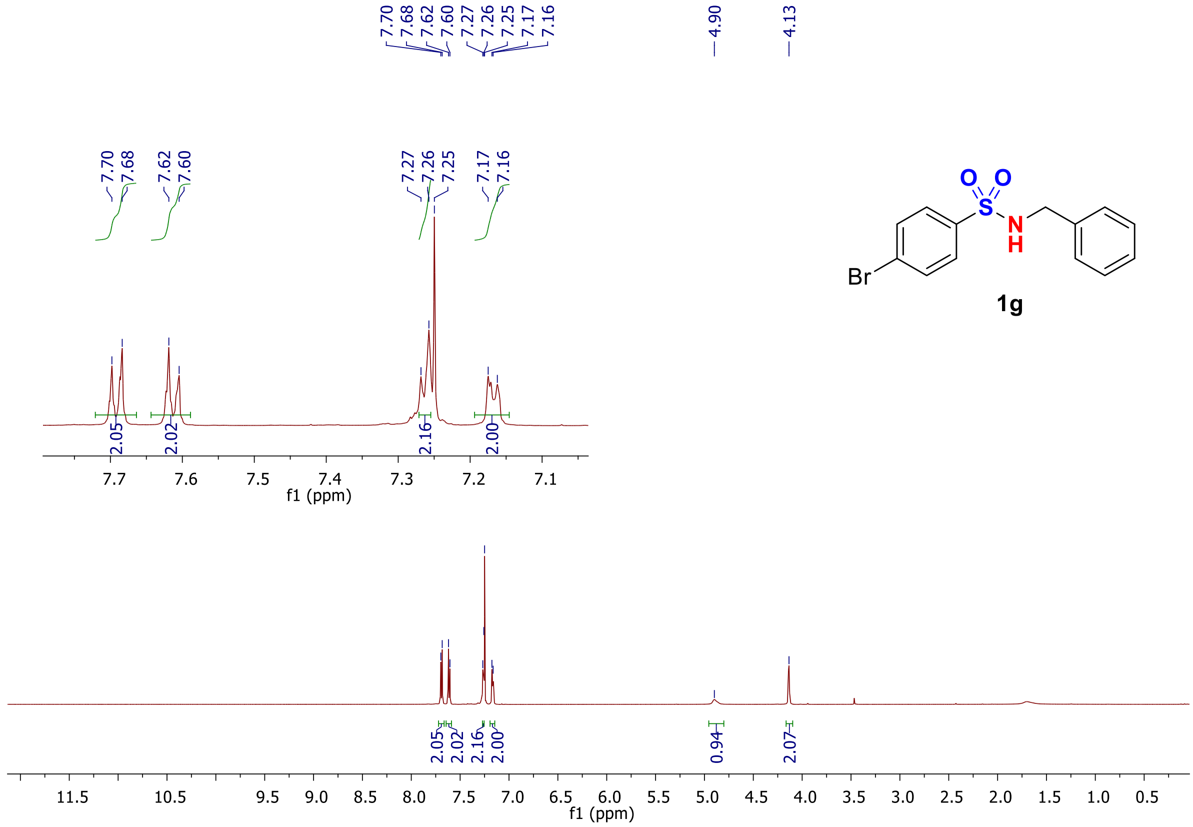

1g

^{13}C{^{1}H} NMR (151 MHz, $CDCl_3$)

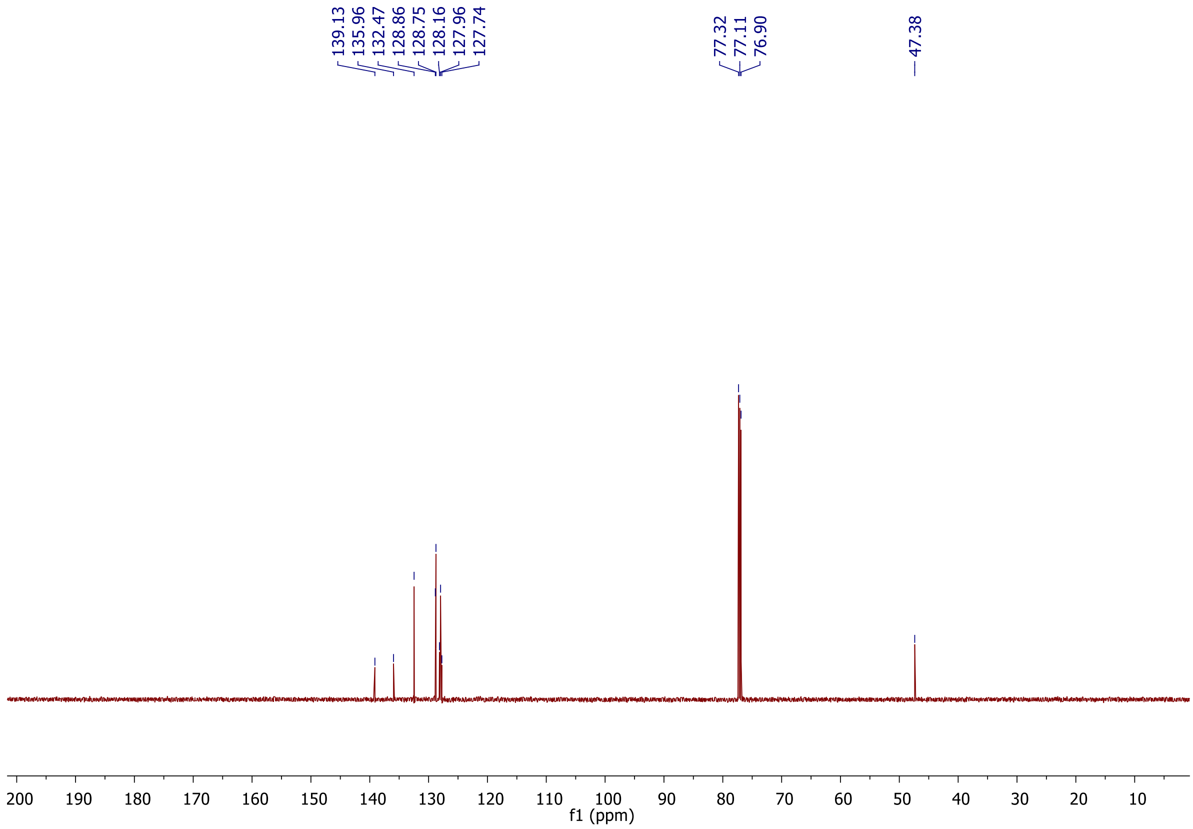

1h ^{1}H NMR (600 MHz, $CDCl_3$)

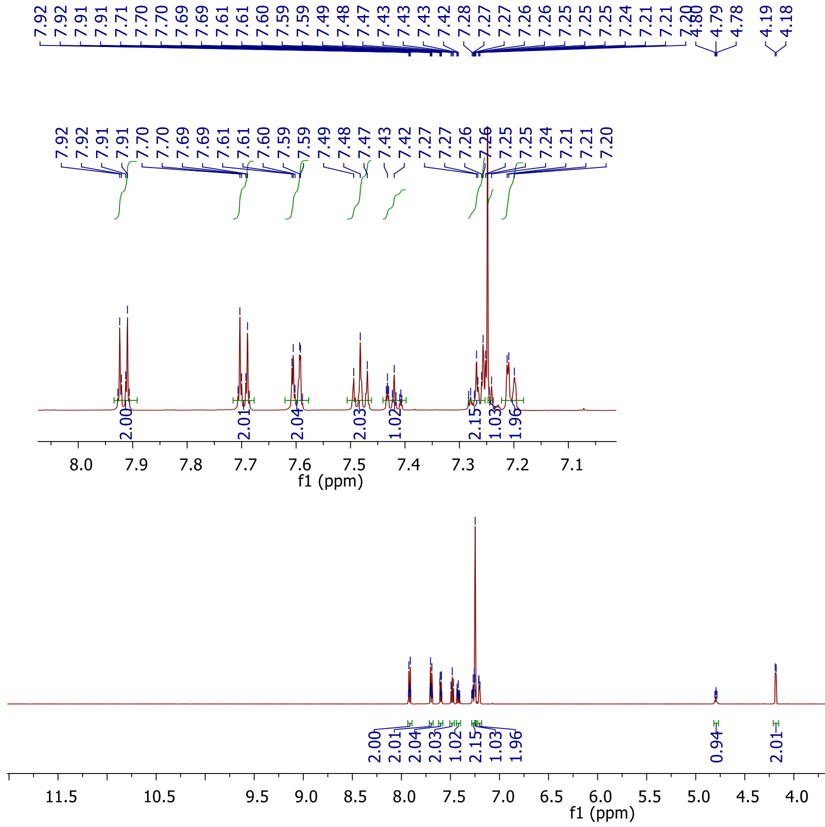

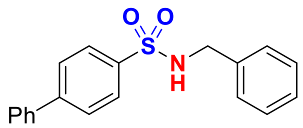

1h

$^{13}C\{^{1}H\}$ NMR (151 MHz, $CDCl_3$)

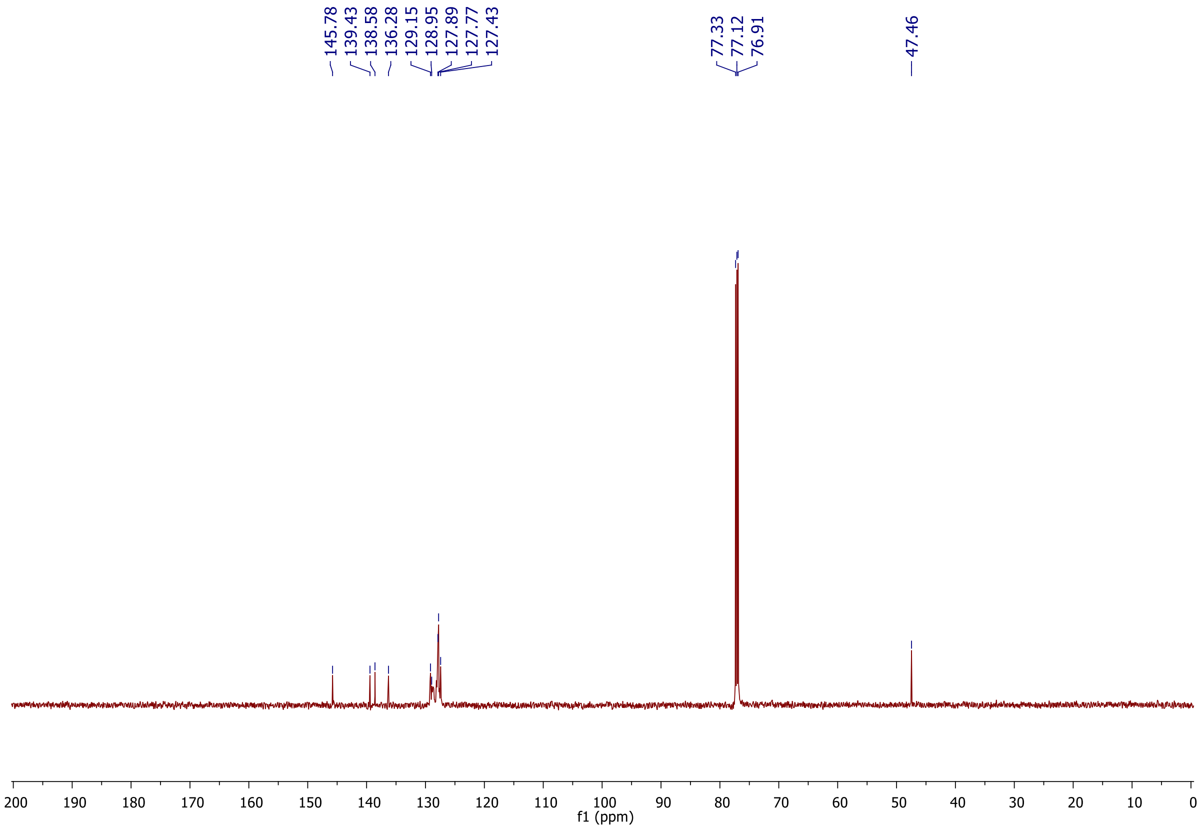

1i ^{1}H NMR (600 MHz, $CDCl_3$)

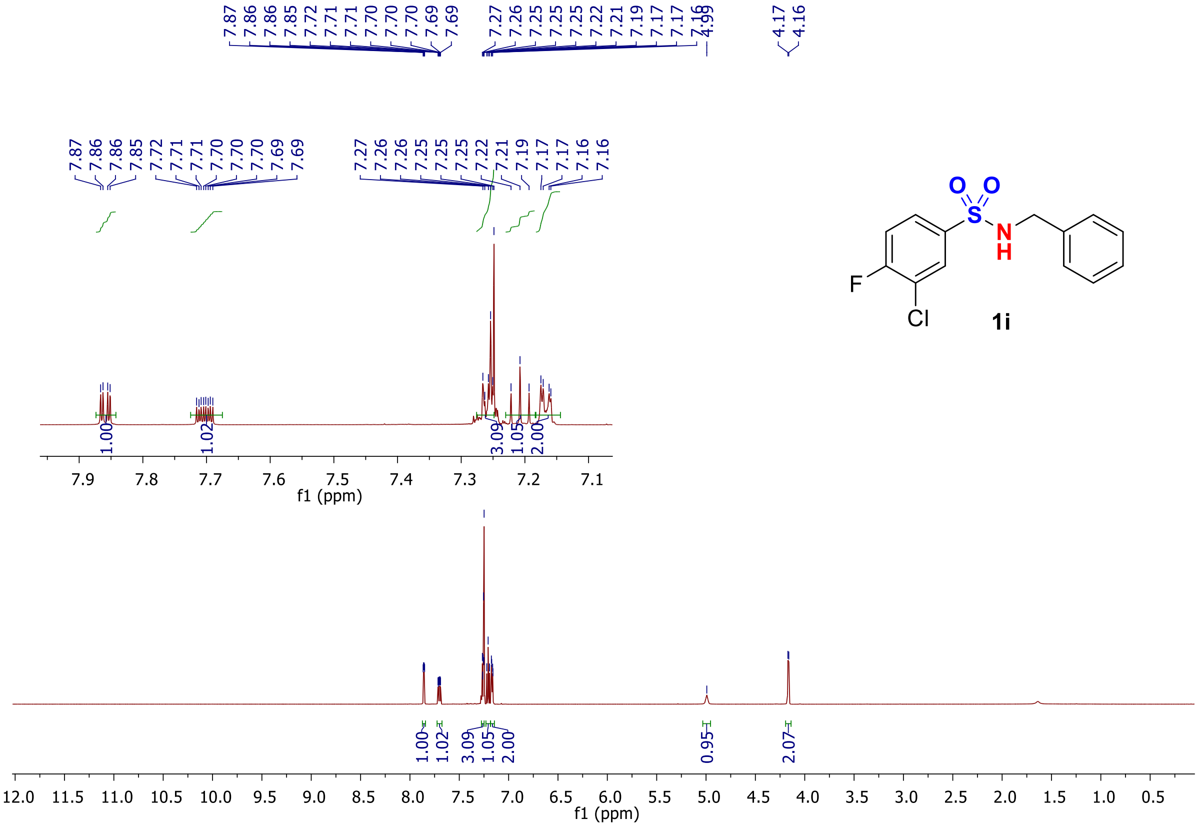

1i

^{13}C{^{1}H} NMR (151 MHz, $CDCl_3$)

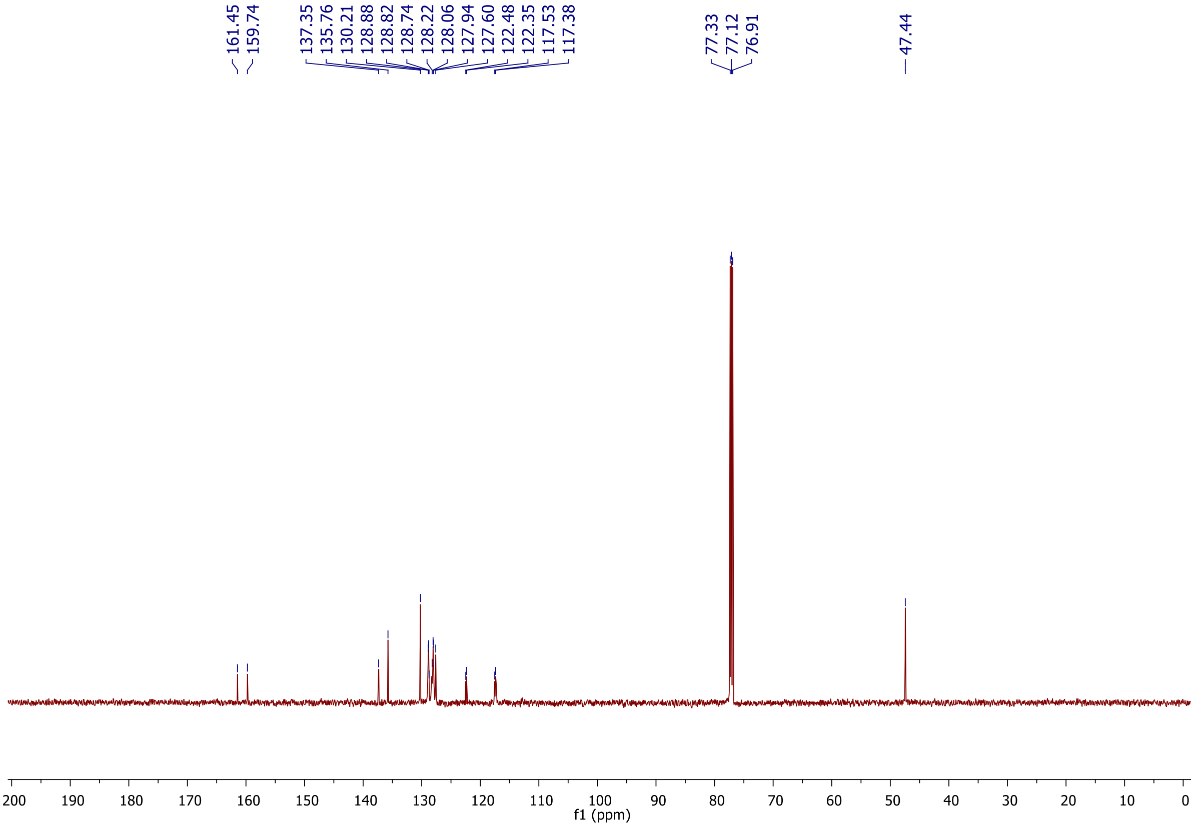

1j ^{1}H NMR (600 MHz, $CDCl_3$)

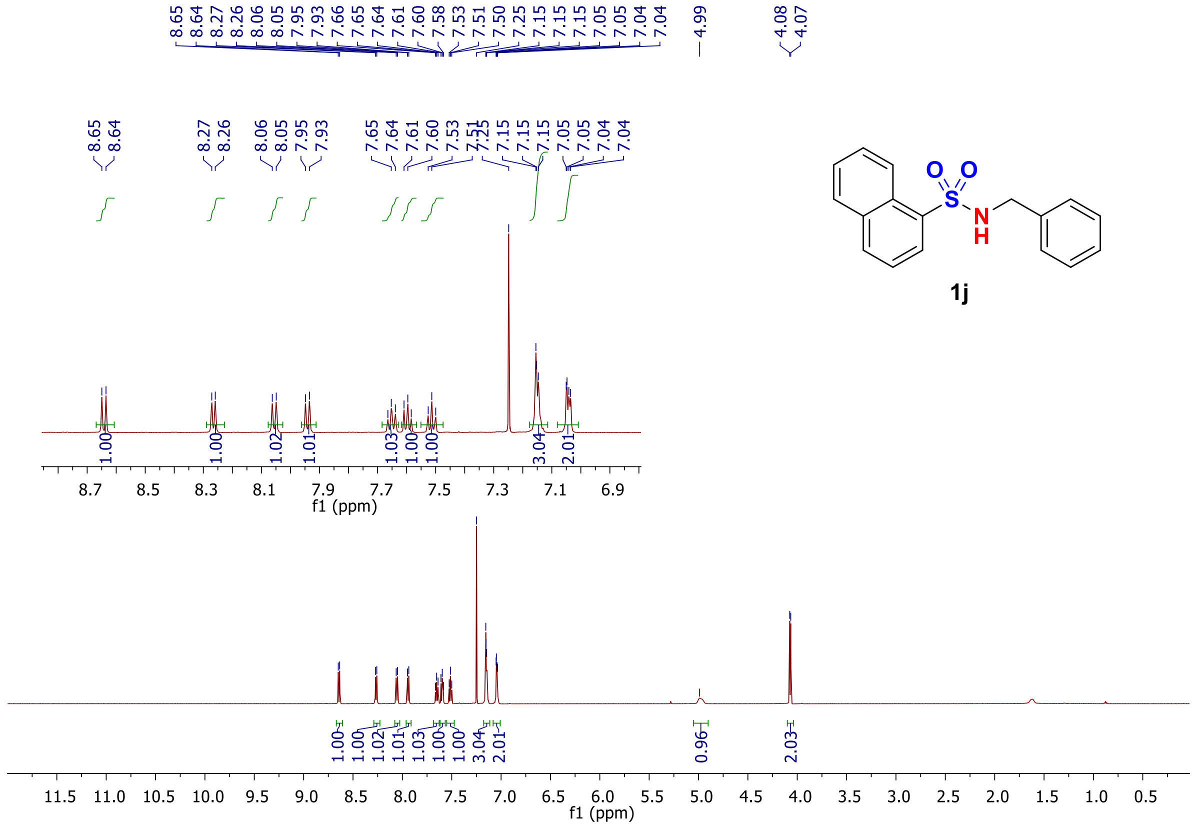

$^{13}C\{^{1}H\}$ NMR (151 MHz, $CDCl_3$)

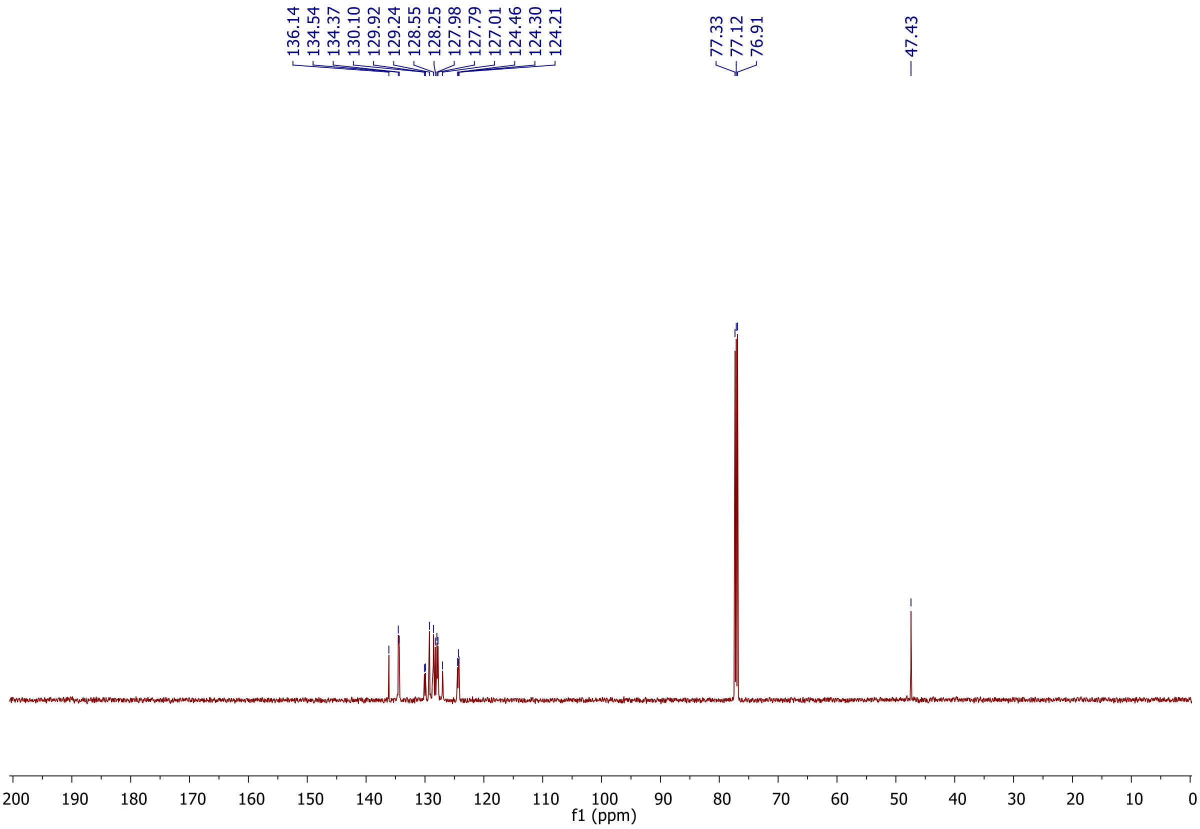

1k ^{1}H NMR (600 MHz, $CDCl_3$)

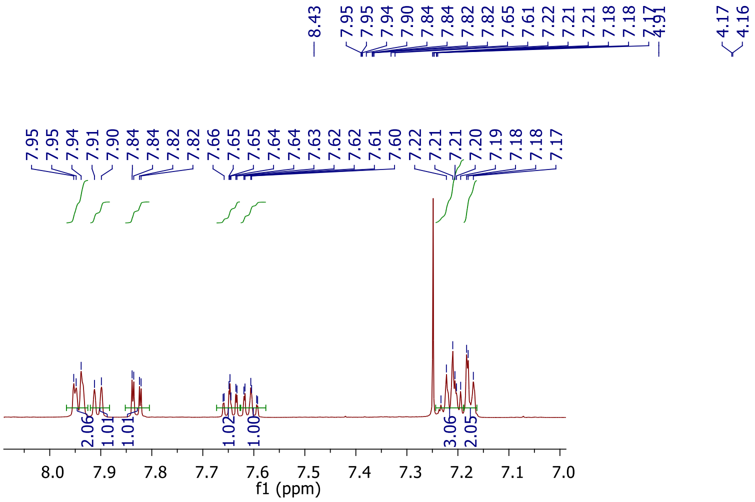

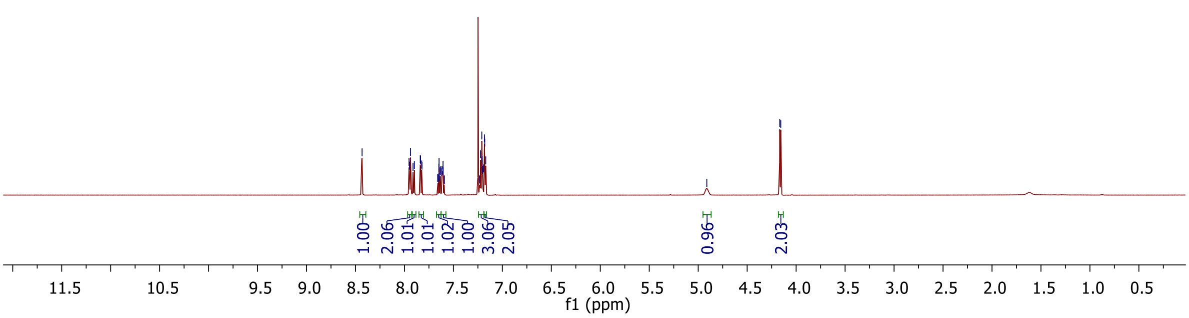

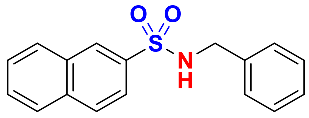

1k

^{13}C{^{1}H} NMR (151 MHz, $CDCl_3$)

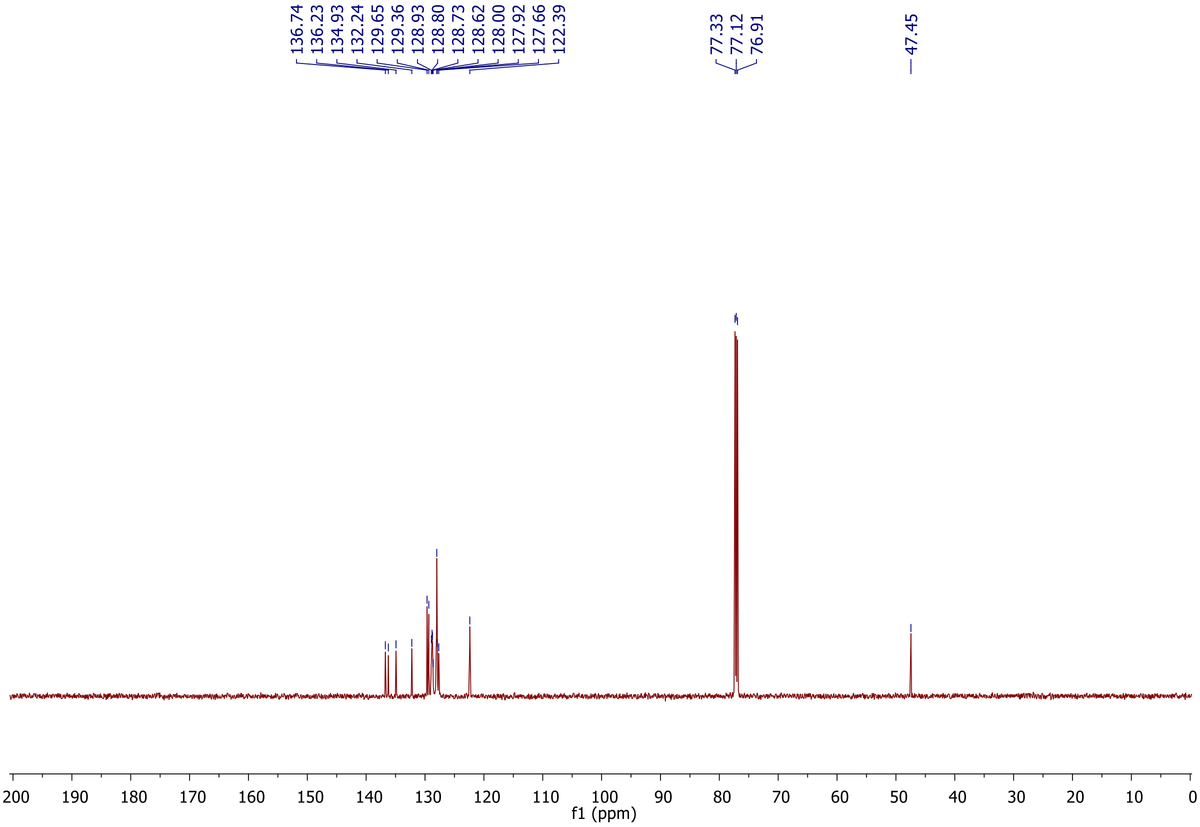

1l ^{1}H NMR (600 MHz, $CDCl_3$)

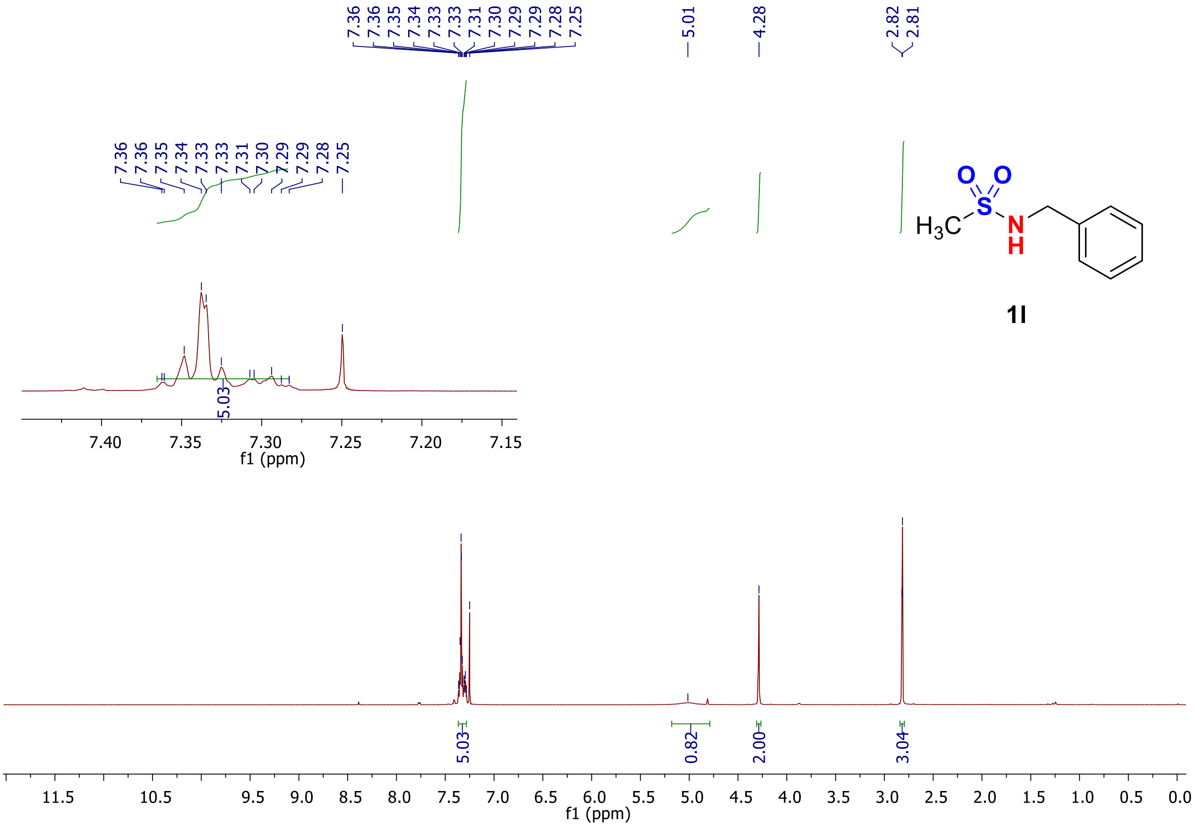

1l

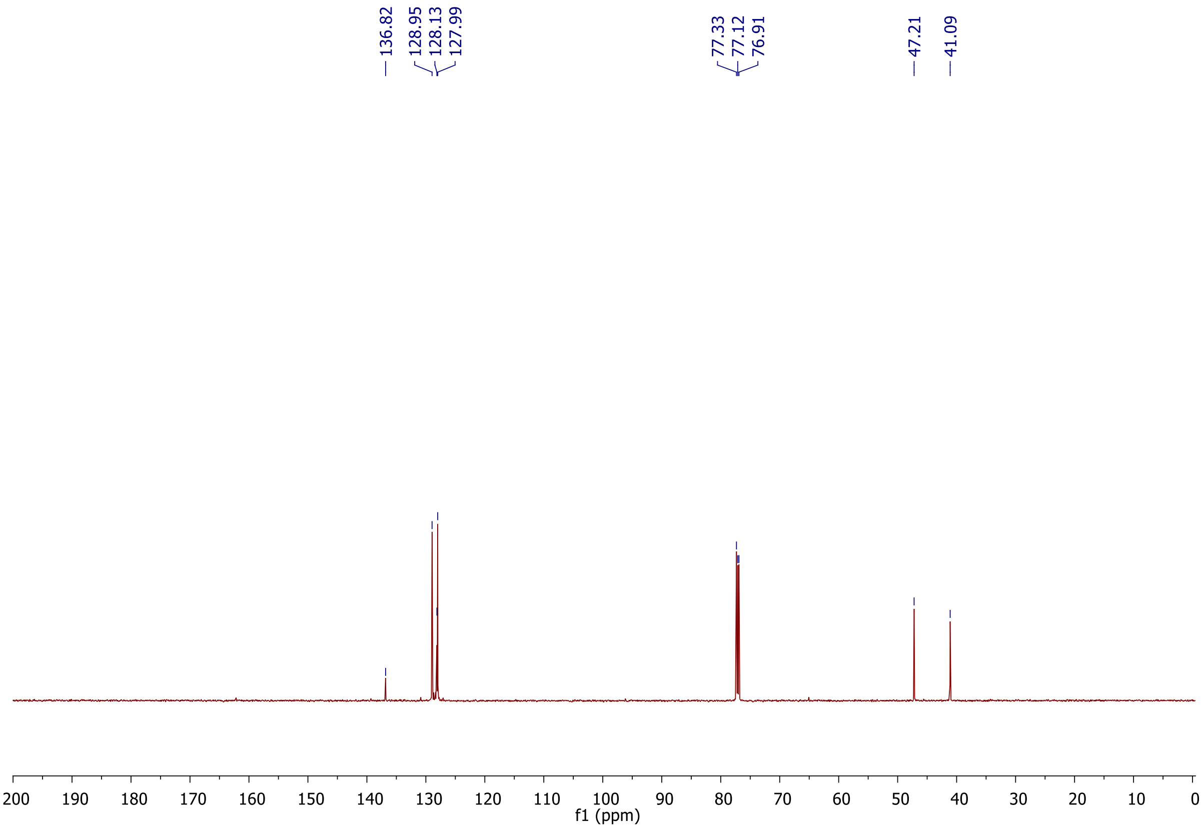

1m ^{1}H NMR (600 MHz, $CDCl_3$)

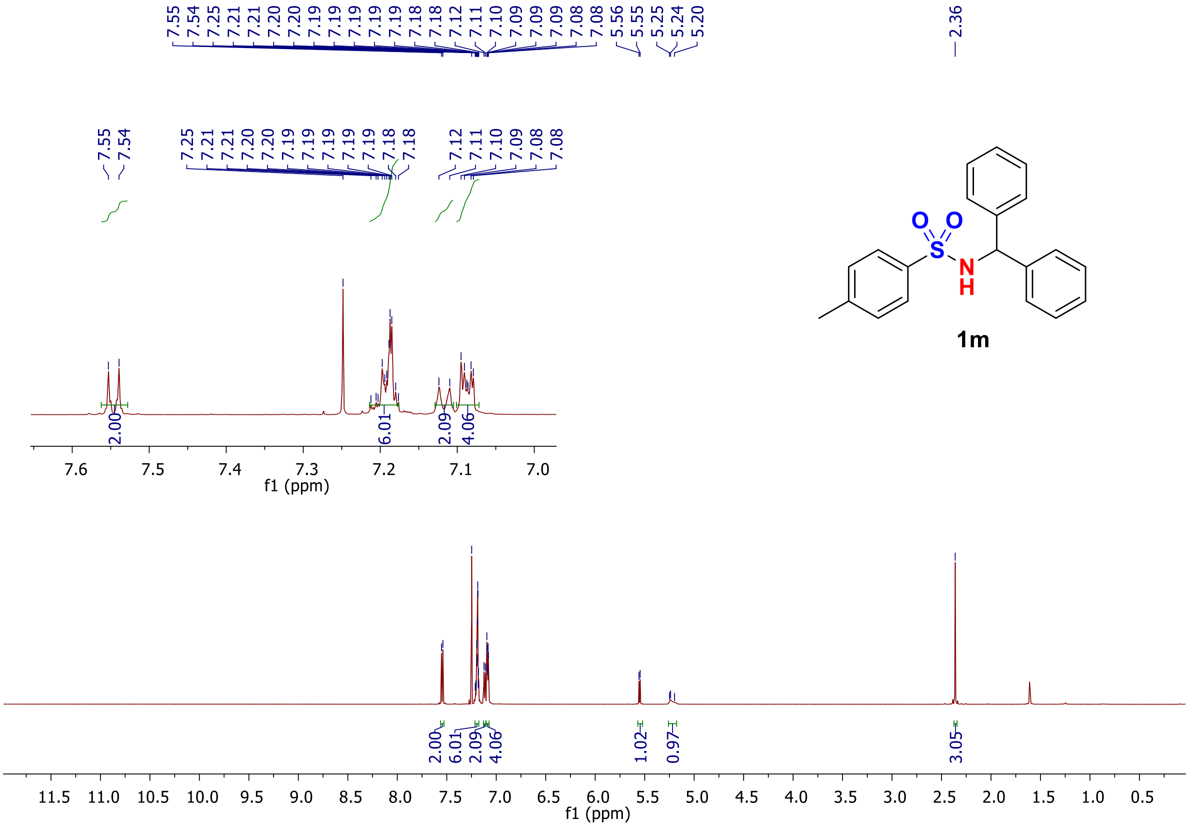

1m

$^{13}C\{^{1}H\}$ NMR (151 MHz, $CDCl_3$)

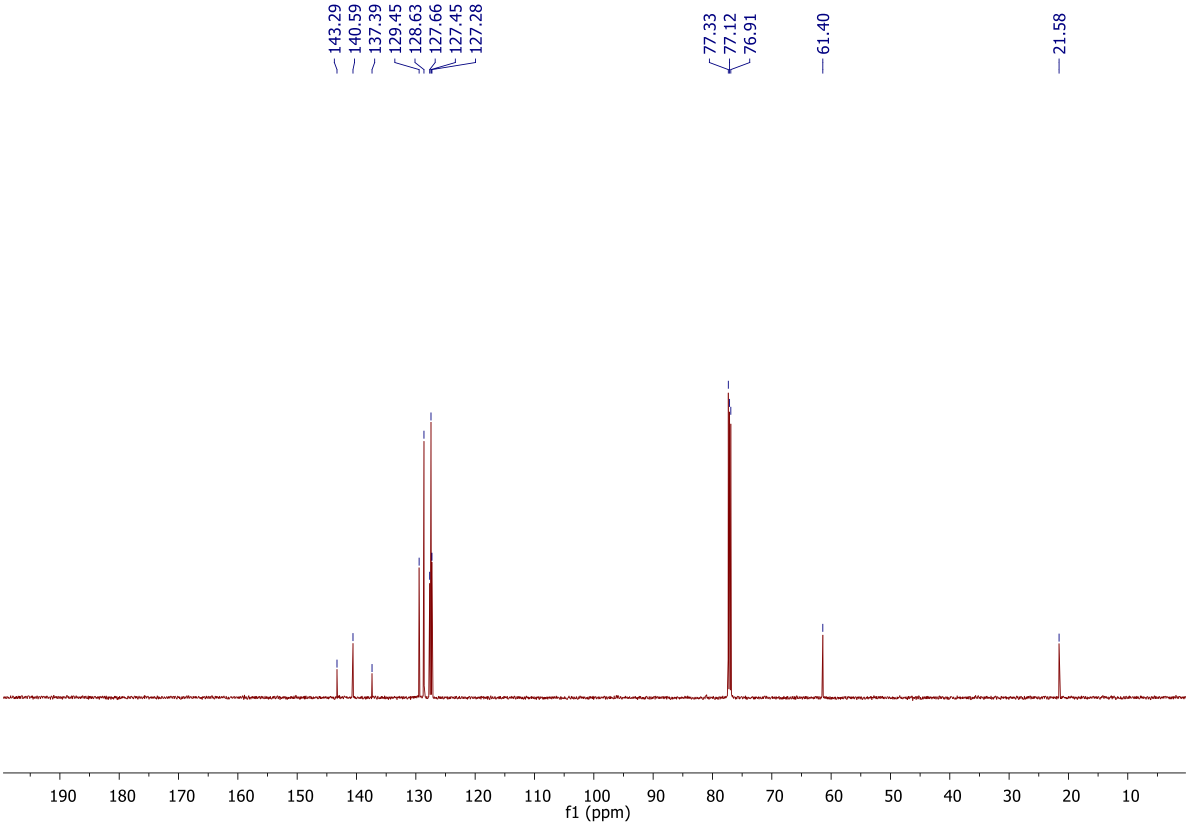

1n ^{1}H NMR (600 MHz, $CDCl_3$)

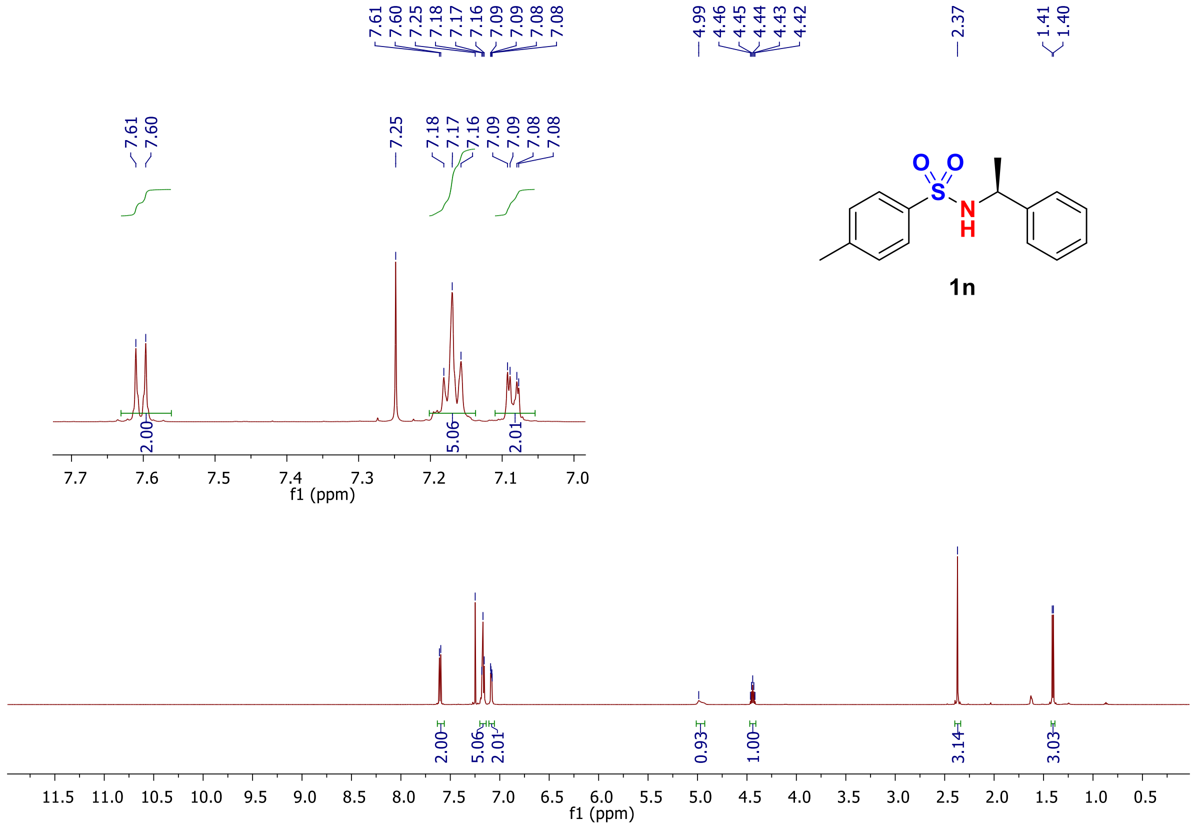

1n

^{13}C{^{1}H} NMR (151 MHz, $CDCl_3$)

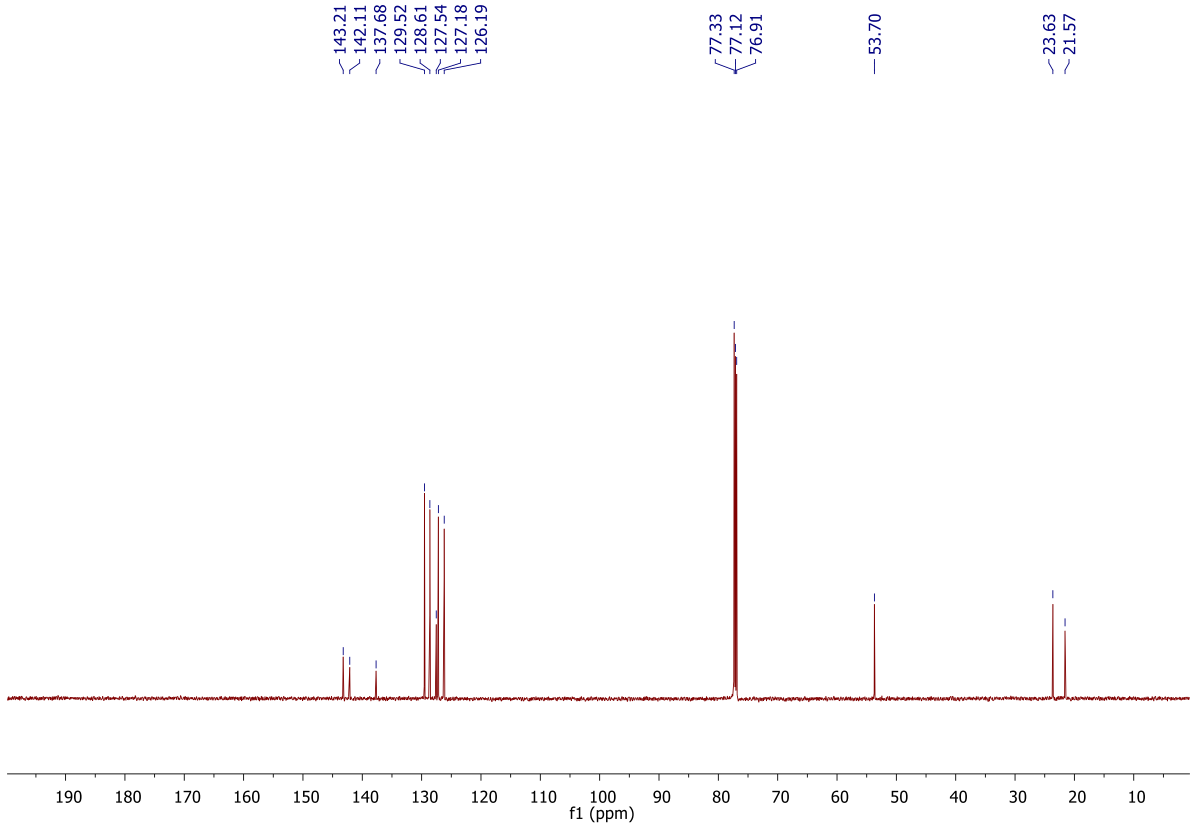

2a ^{1}H NMR (600 MHz, $CDCl_3$)

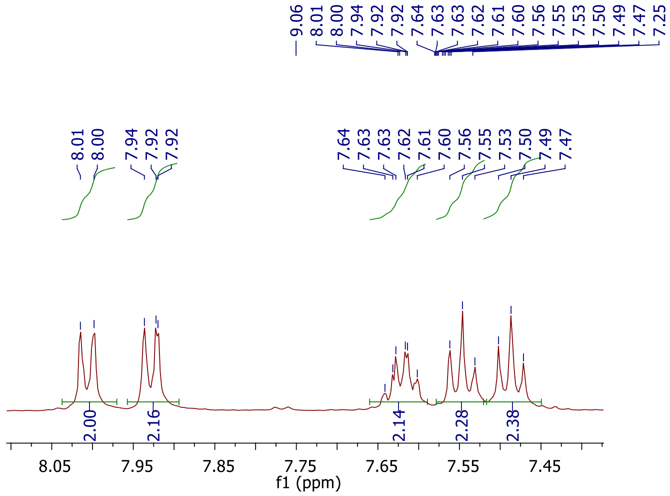

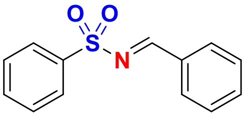

2a

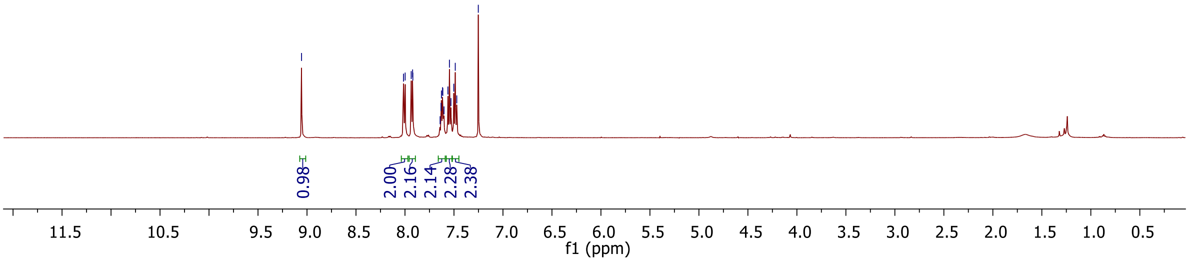

^{13}C{^{1}H} NMR (151 MHz, $CDCl_3$)

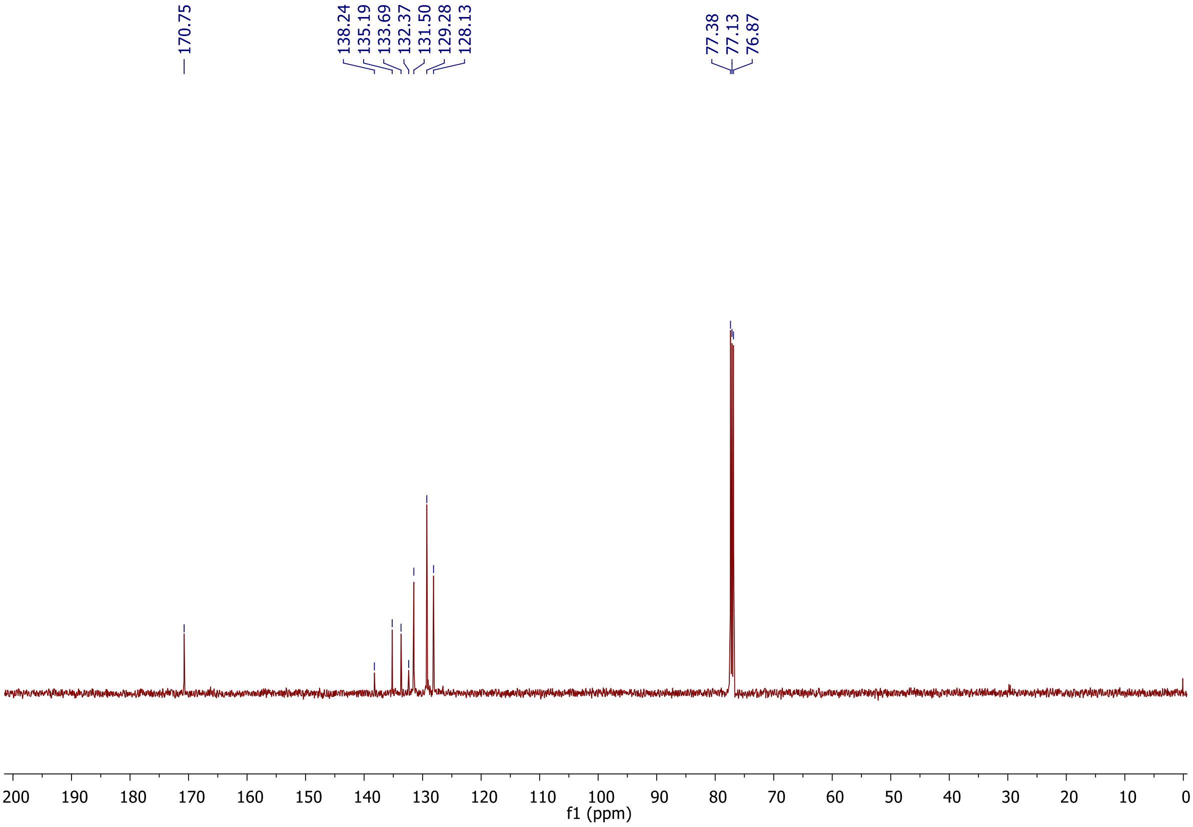

2b ^{1}H NMR (600 MHz, $CDCl_3$)

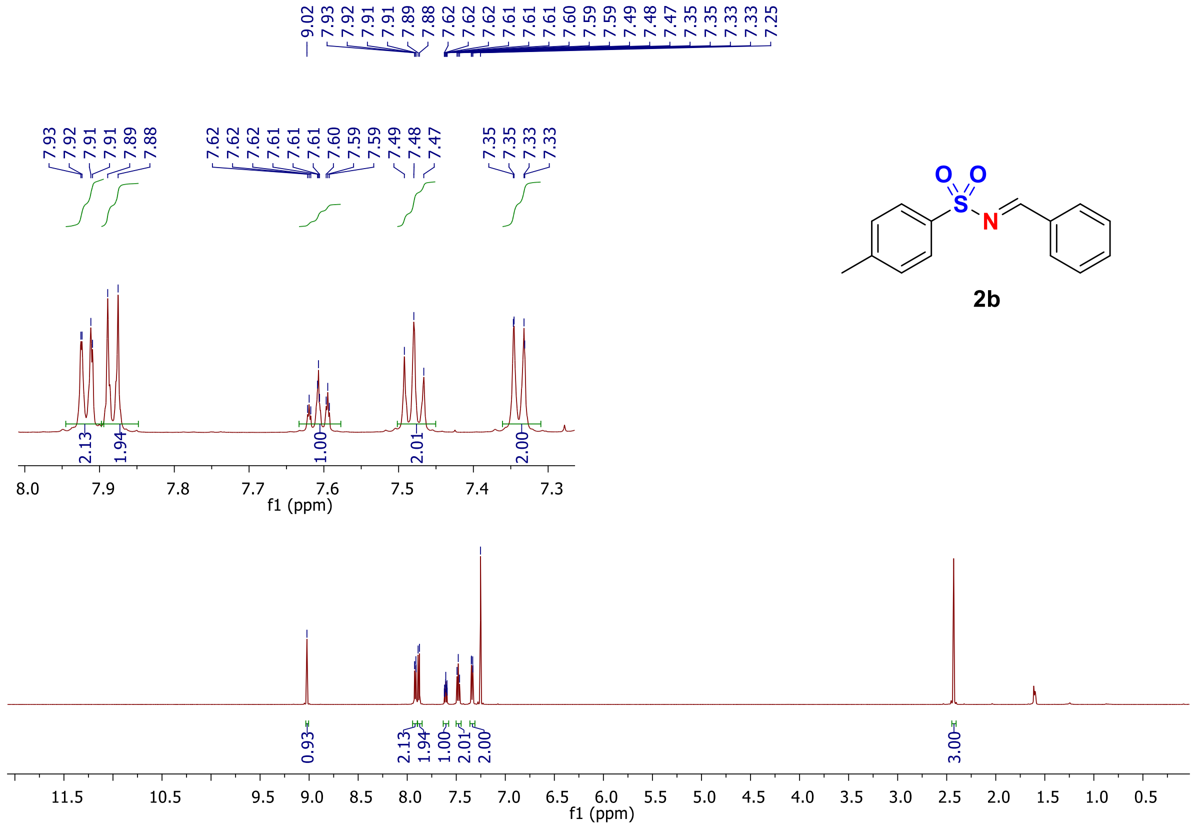

2b

^{13}C{^{1}H} NMR (151 MHz, $CDCl_3$)

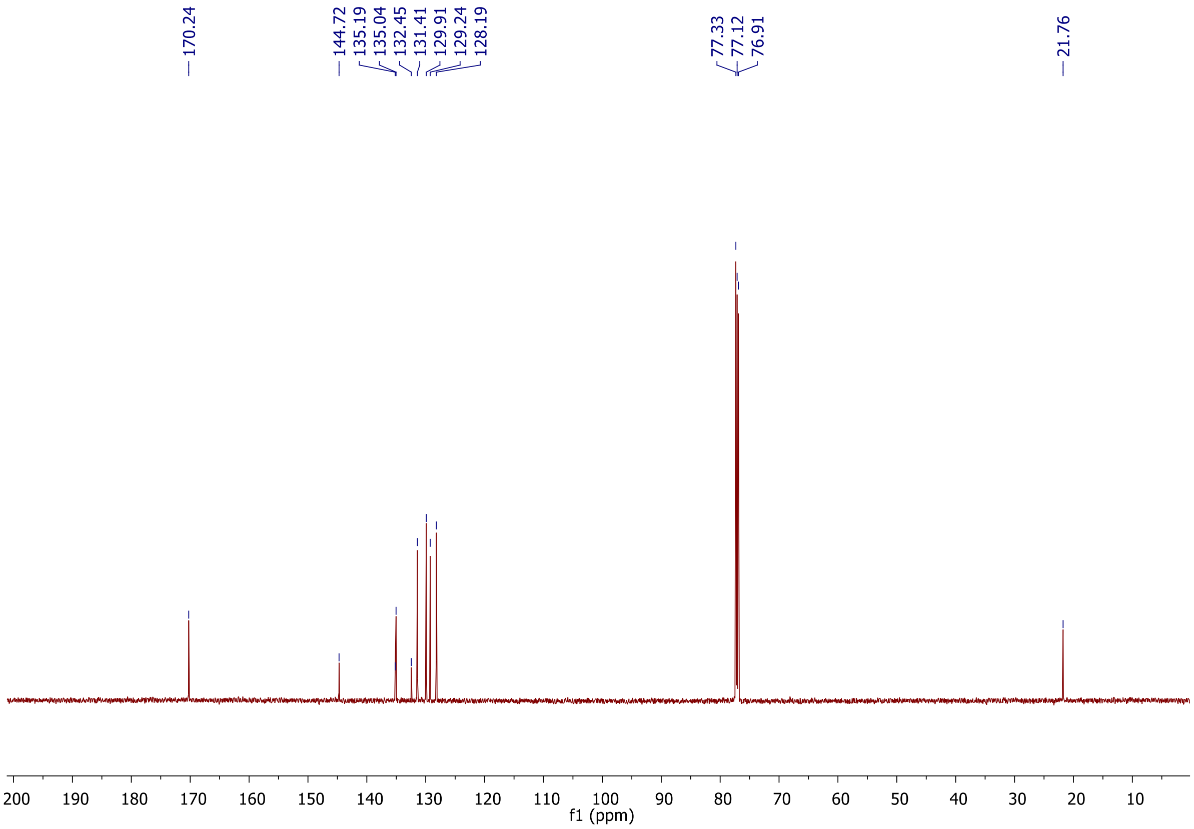

2c ^{1}H NMR (600 MHz, $CDCl_3$)

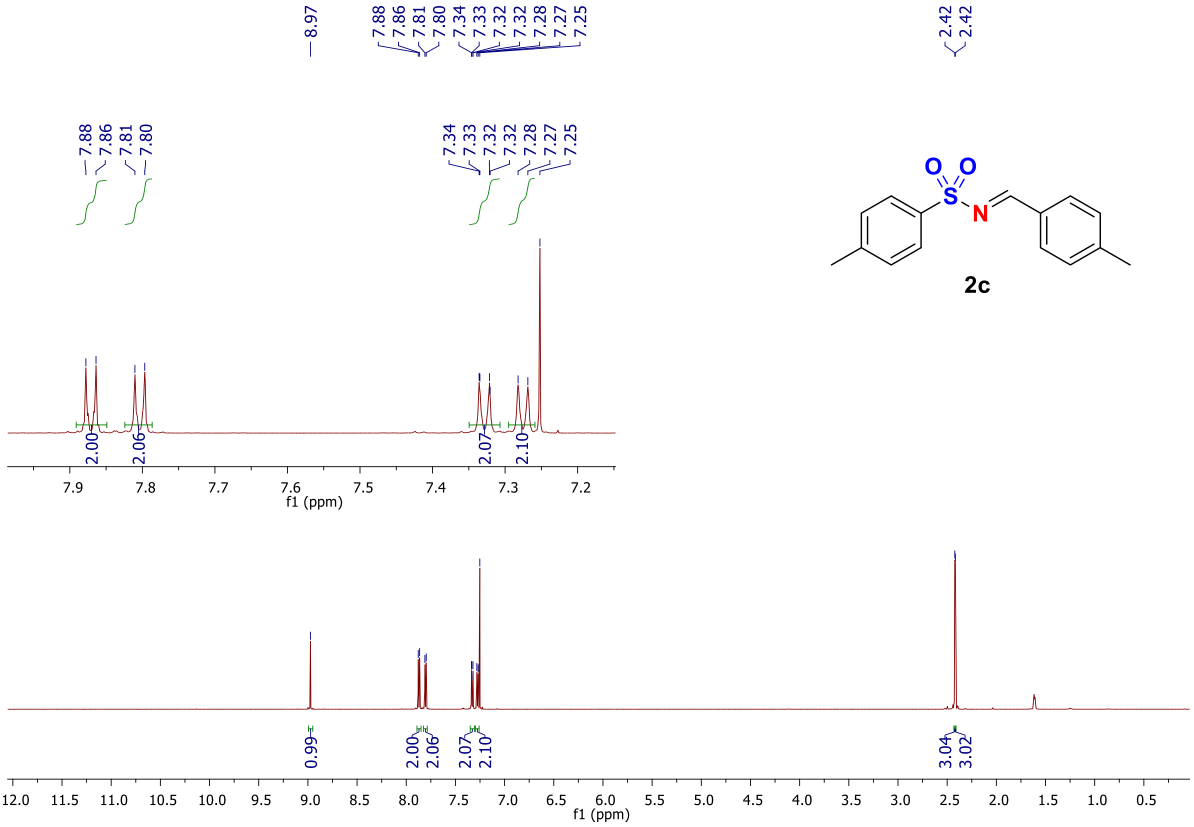

2c

^{13}C{^{1}H} NMR (151 MHz, $CDCl_3$)

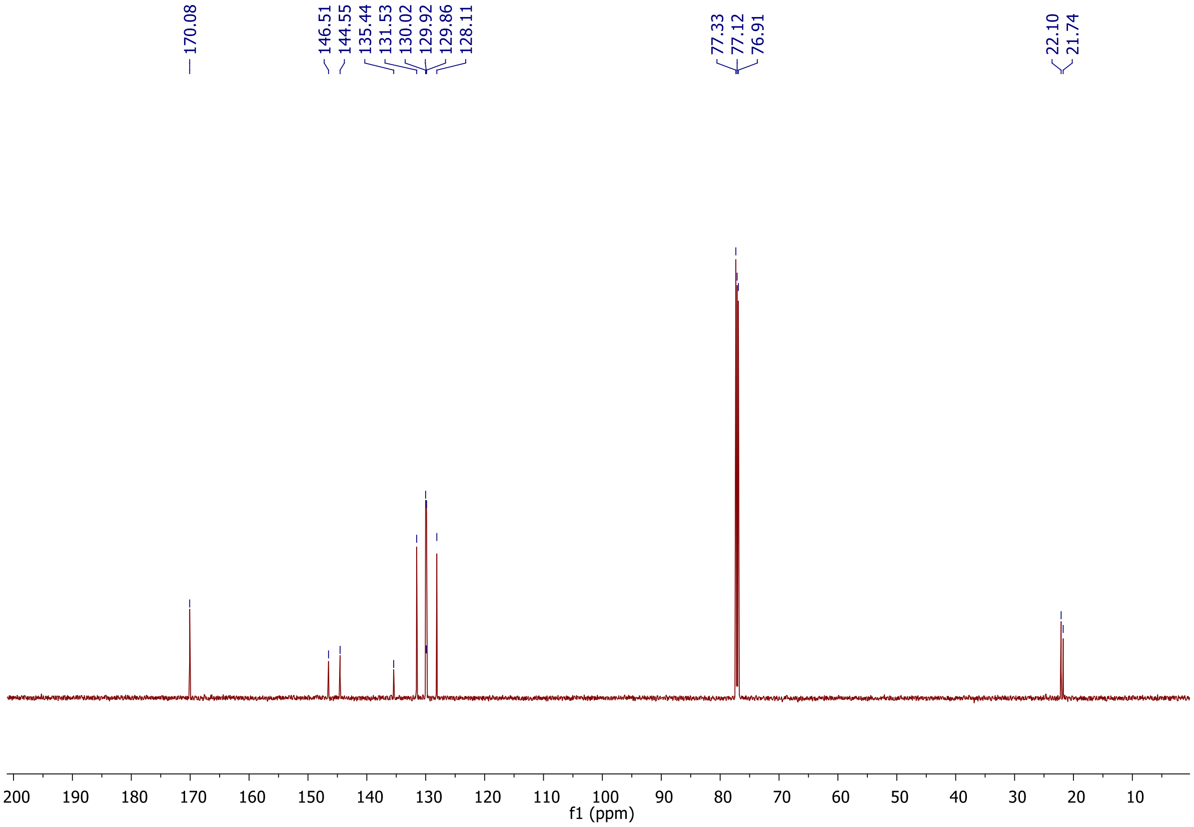

2d ^{1}H NMR (600 MHz, $CDCl_3$)

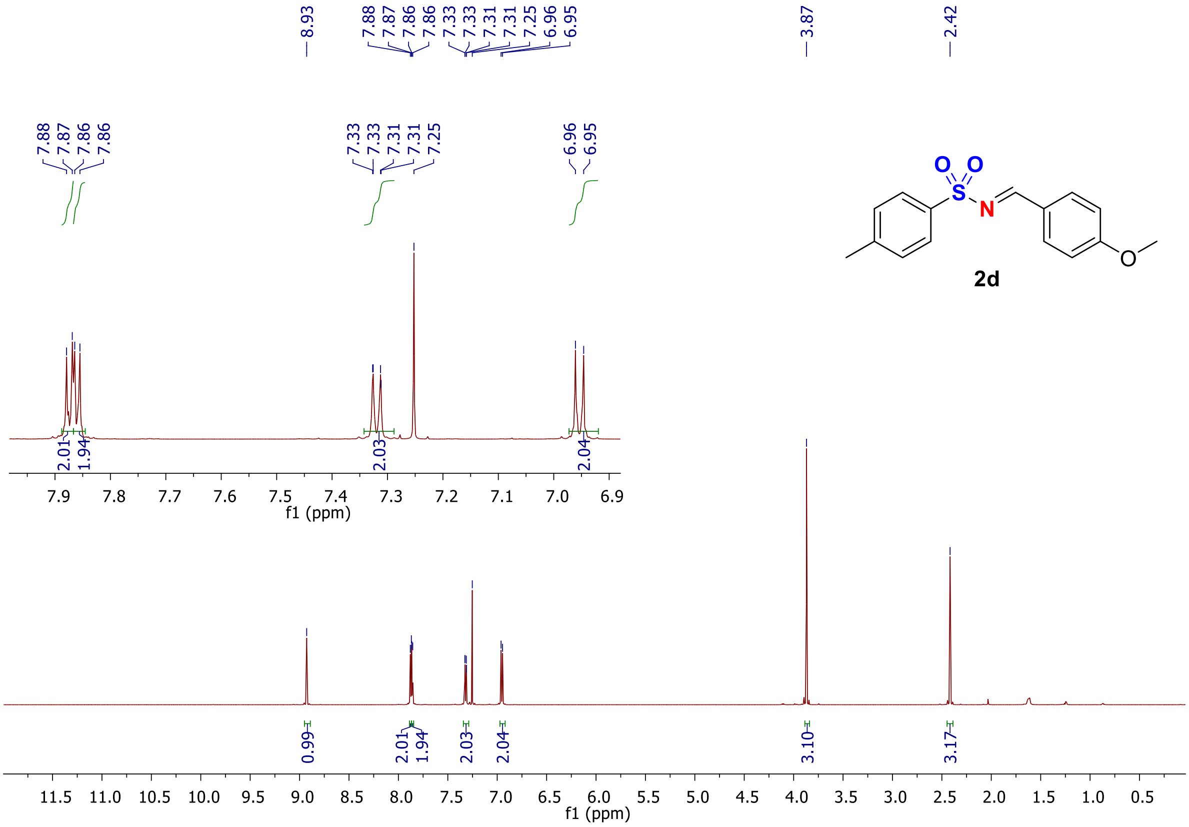

^{13}C{^{1}H} NMR (151 MHz, $CDCl_3$)

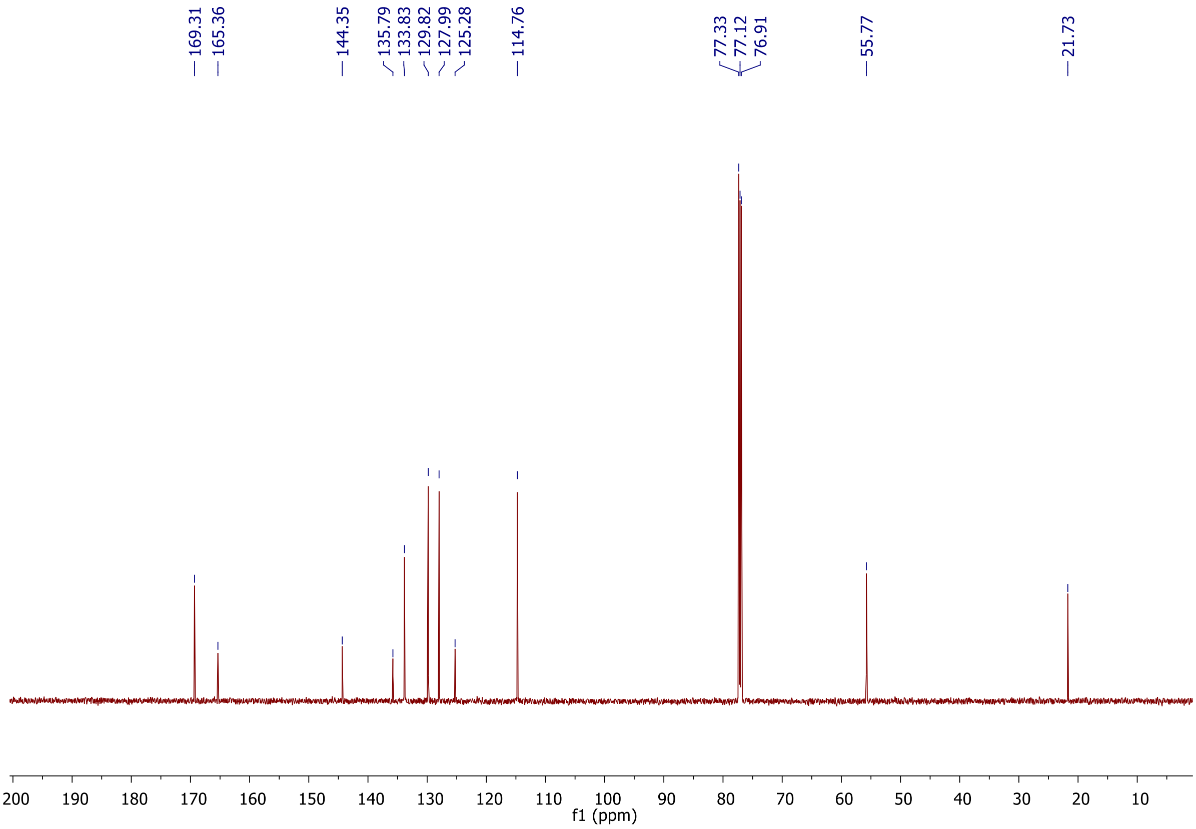

2e ^{1}H NMR (600 MHz, $CDCl_3$)

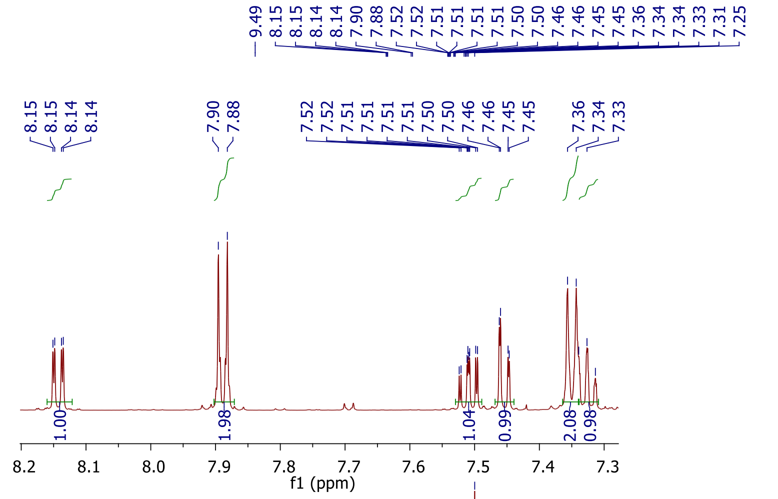

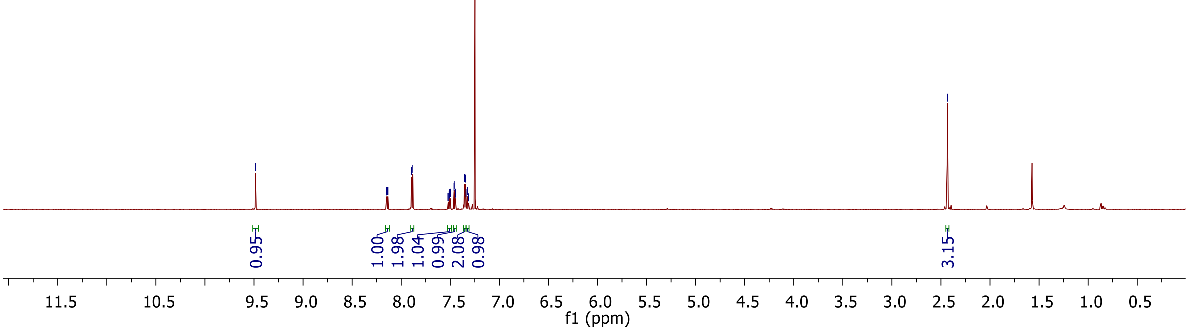

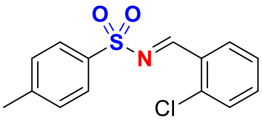
2e

^{13}C{^{1}H} NMR (151 MHz, $CDCl_3$)

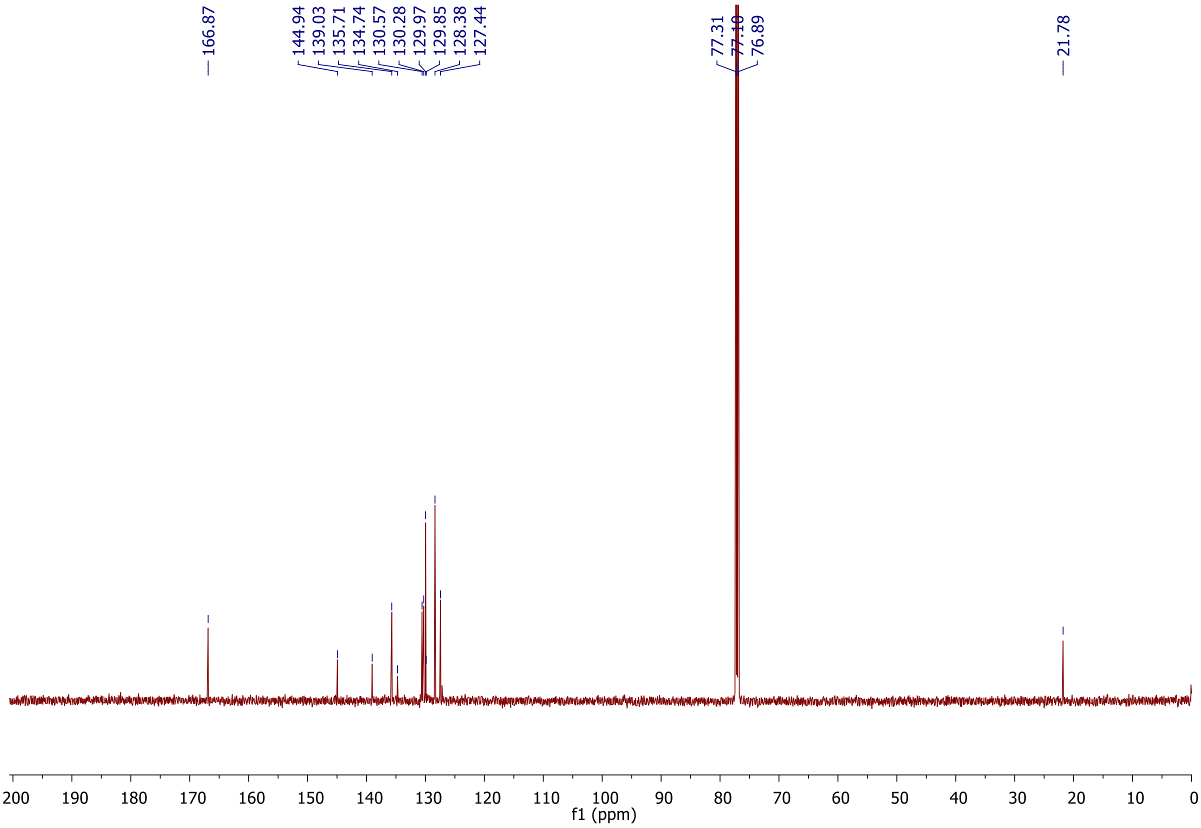

2f ^{1}H NMR (600 MHz, $CDCl_3$)

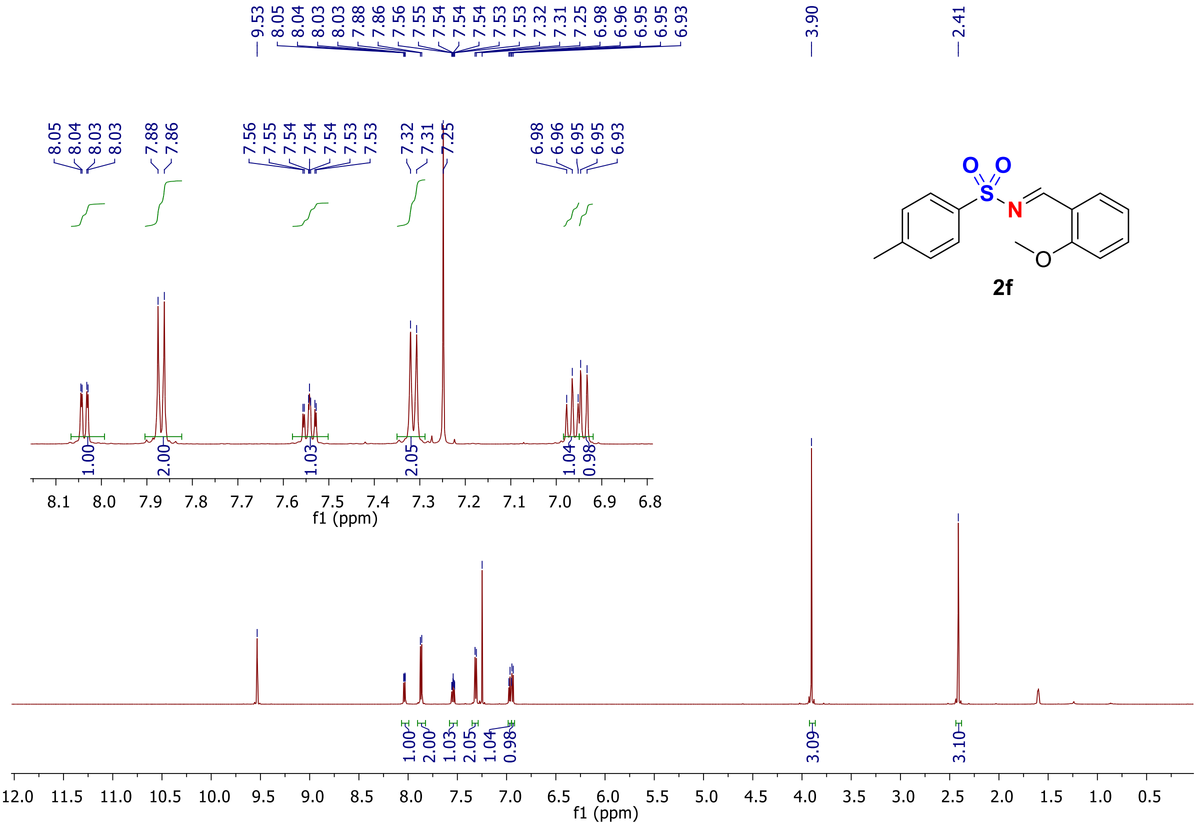

^{13}C{^{1}H} NMR (151 MHz, $CDCl_3$)

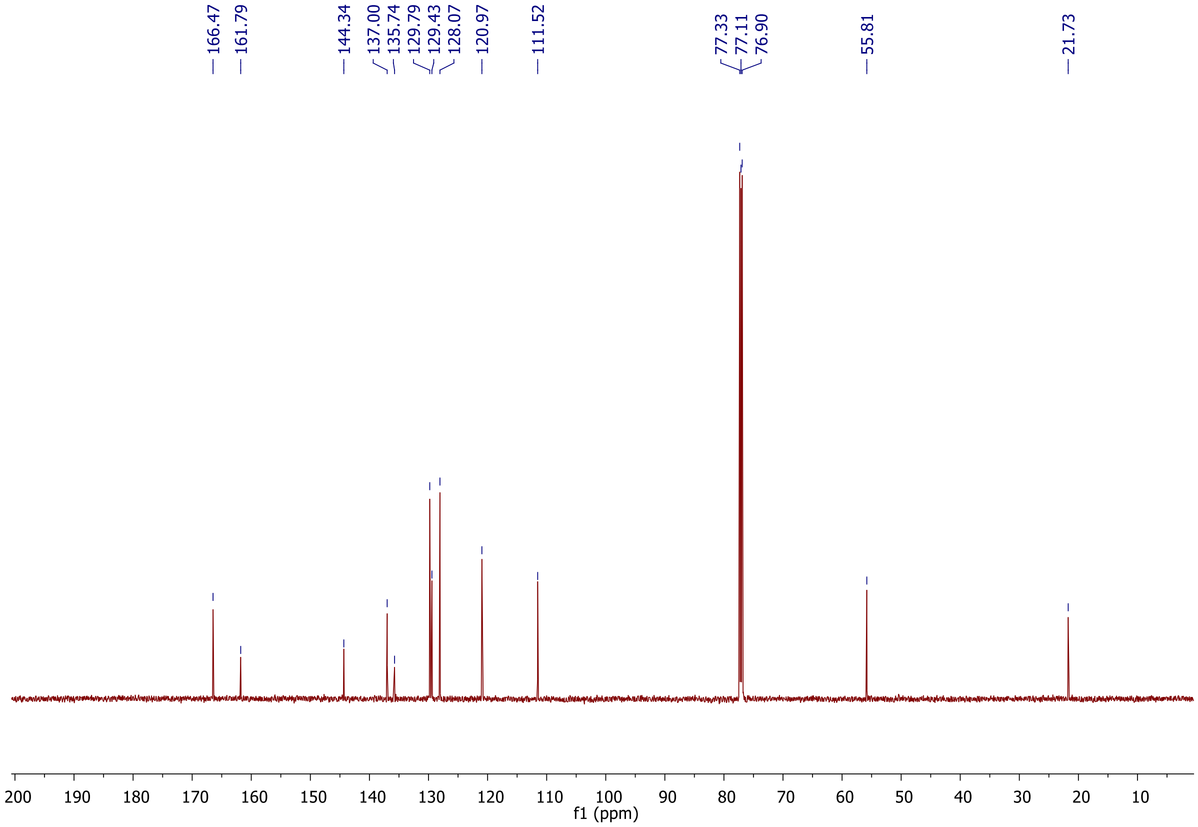

2g ^{1}H NMR (600 MHz, $CDCl_3$)

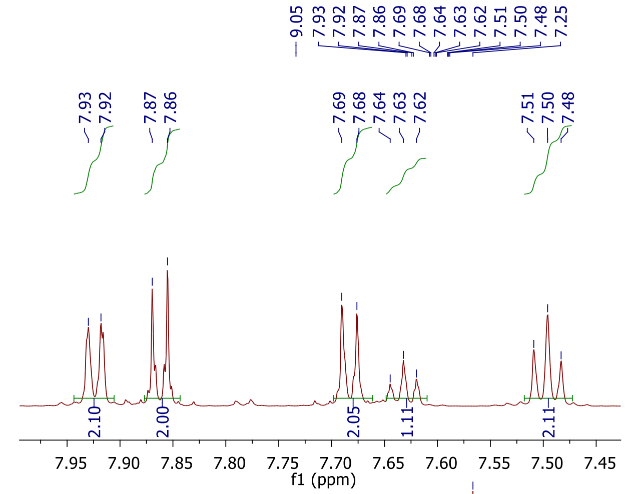

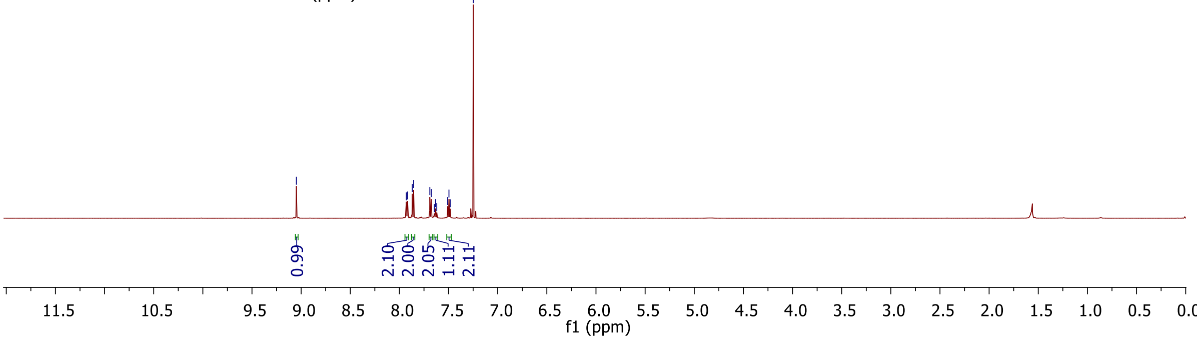

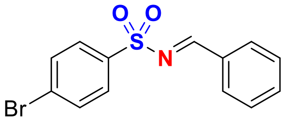

$^{13}C\{^{1}H\}$ NMR (151 MHz, $CDCl_3$)

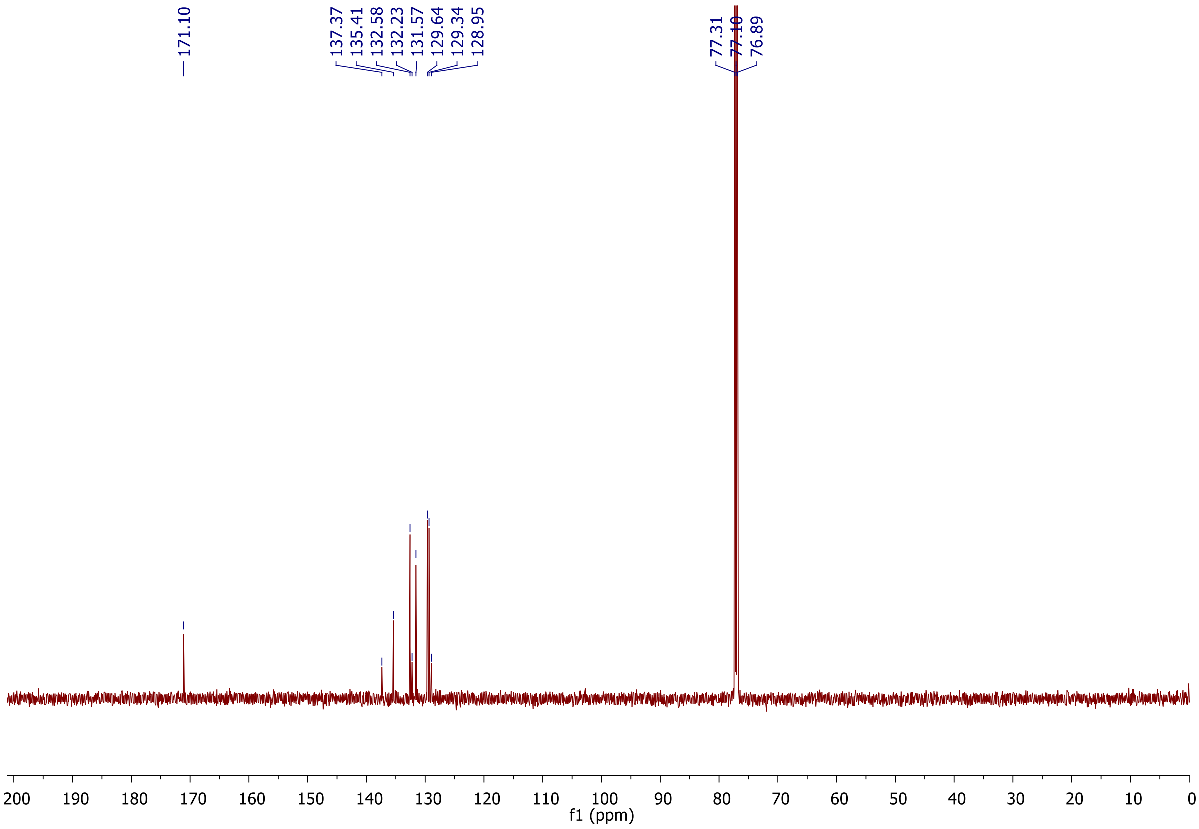

2h ^{1}H NMR (600 MHz, $CDCl_3$)

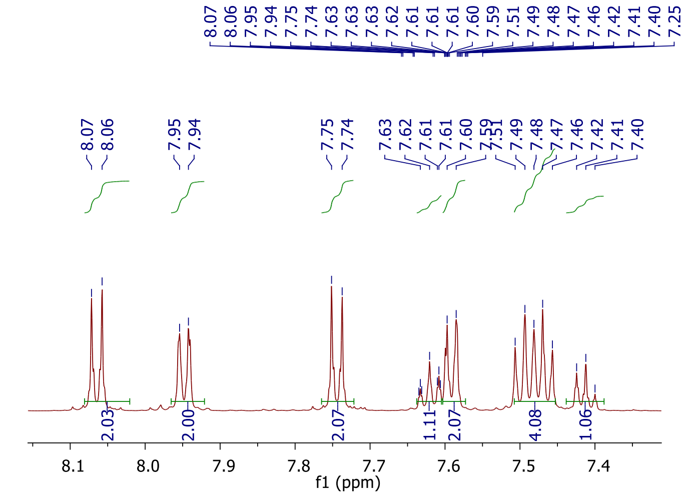

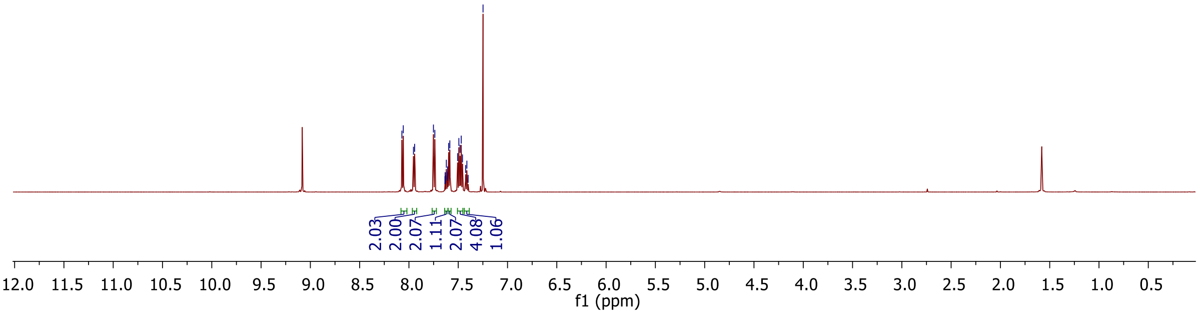

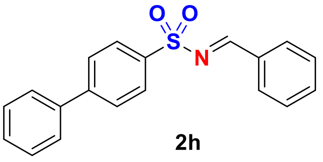

2h

^{13}C{^{1}H} NMR (151 MHz, $CDCl_3$)

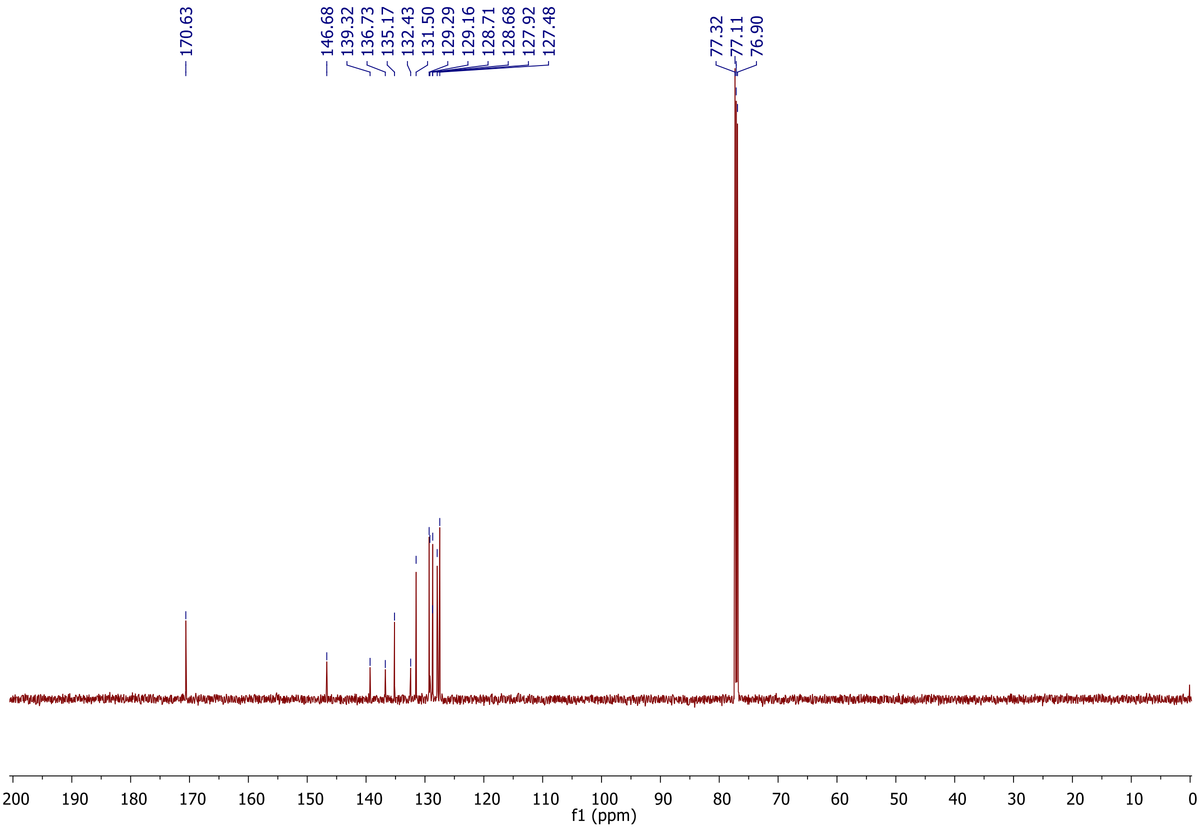

2i ^{1}H NMR (600 MHz, $CDCl_3$)

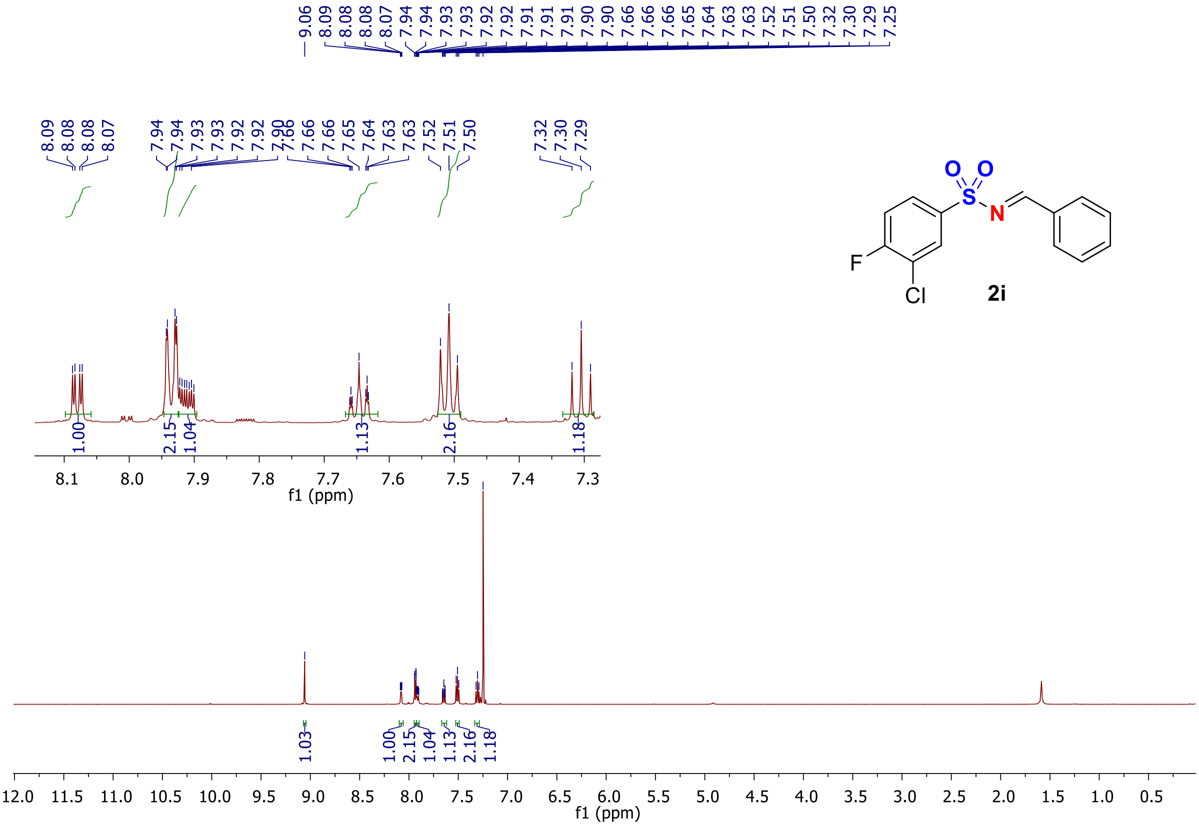

2i

^{13}C{^{1}H} NMR (151 MHz, $CDCl_3$)

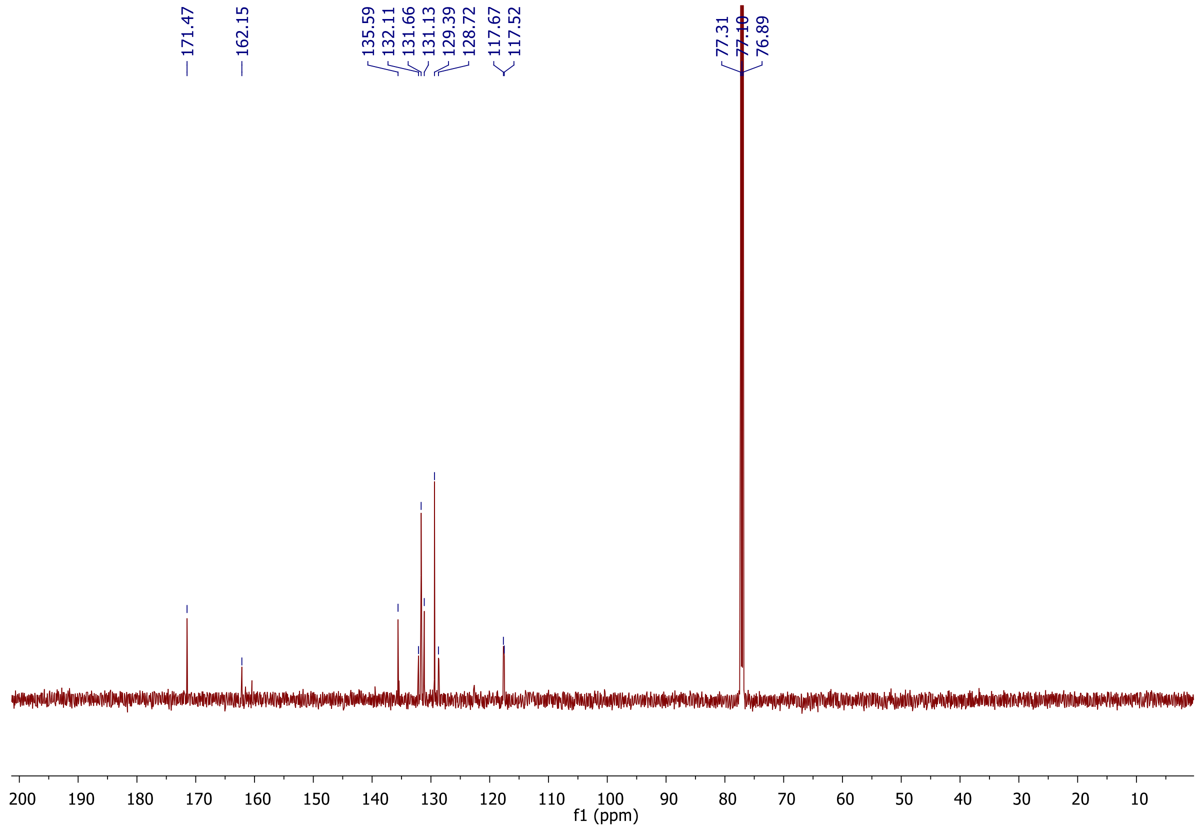

2j ^{1}H NMR (600 MHz, $CDCl_3$)

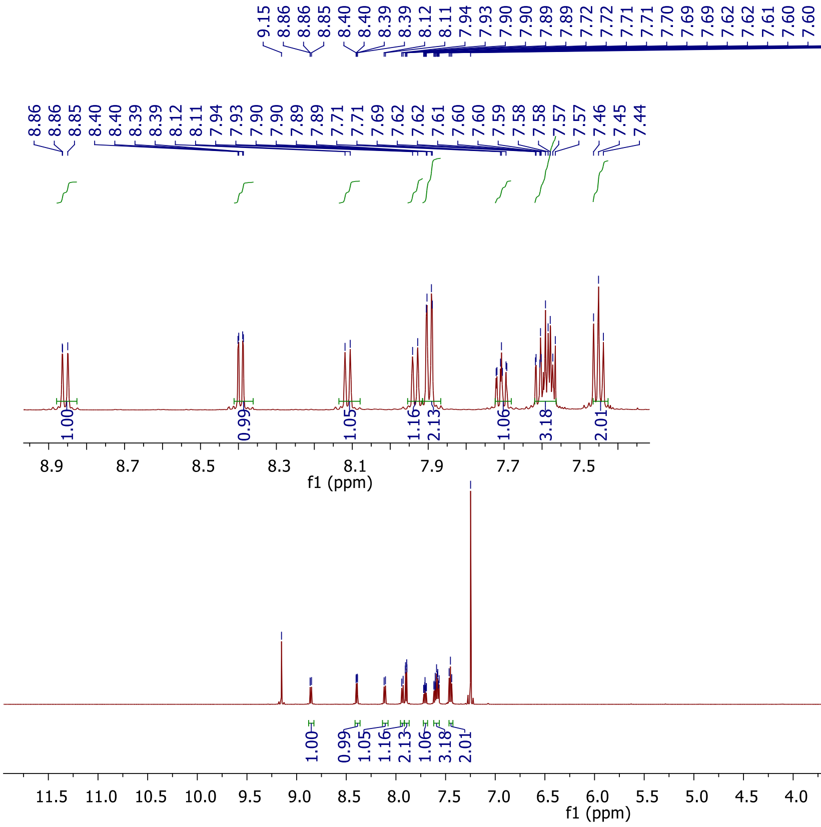

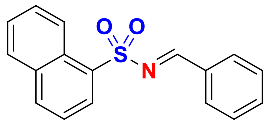

2j

^{13}C{^{1}H} NMR (151 MHz, $CDCl_3$)

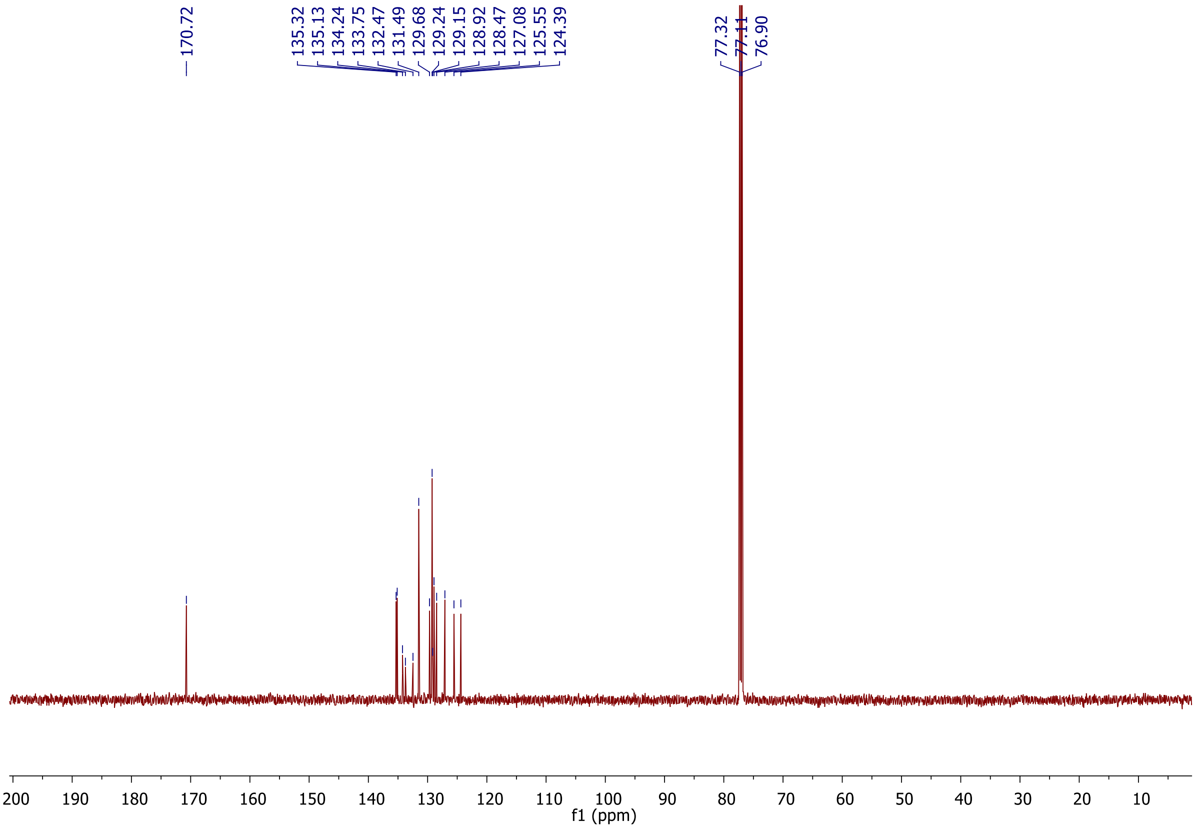

2k ^{1}H NMR (600 MHz, $CDCl_3$)

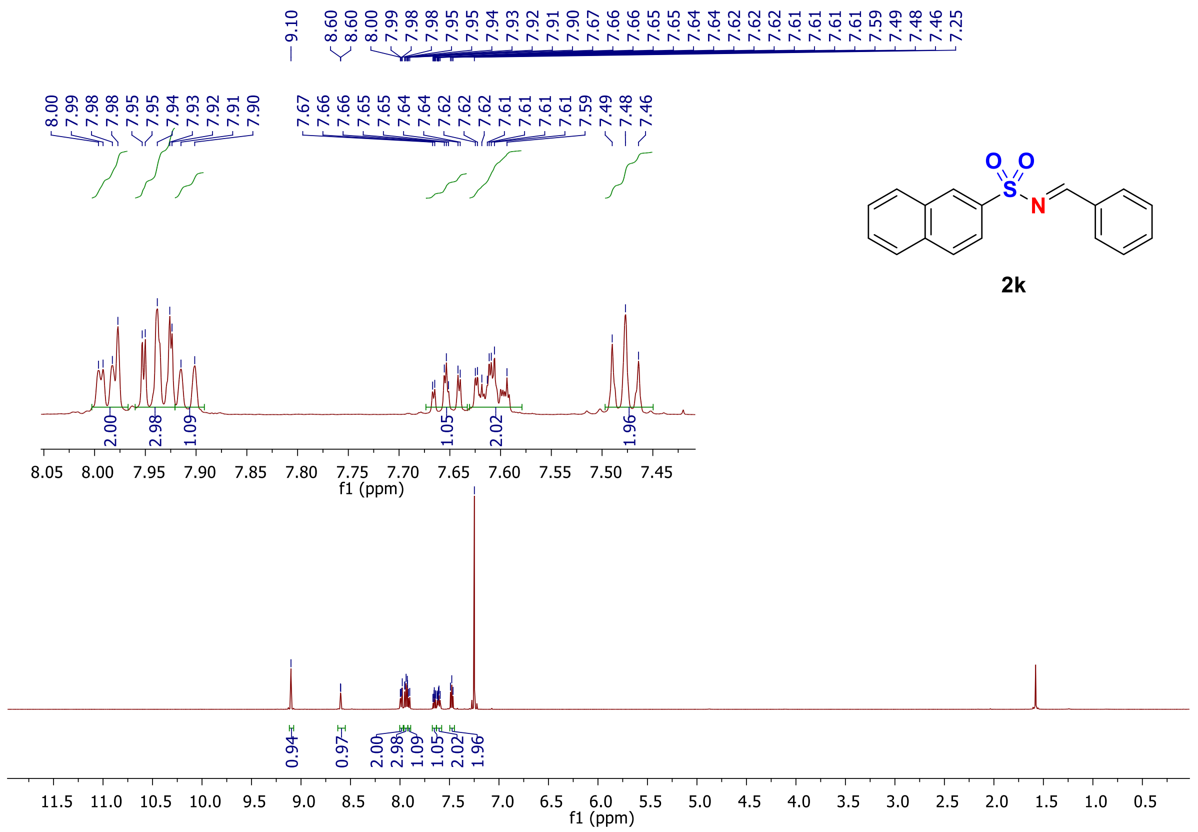

^{13}C{^{1}H} NMR (151 MHz, $CDCl_3$)

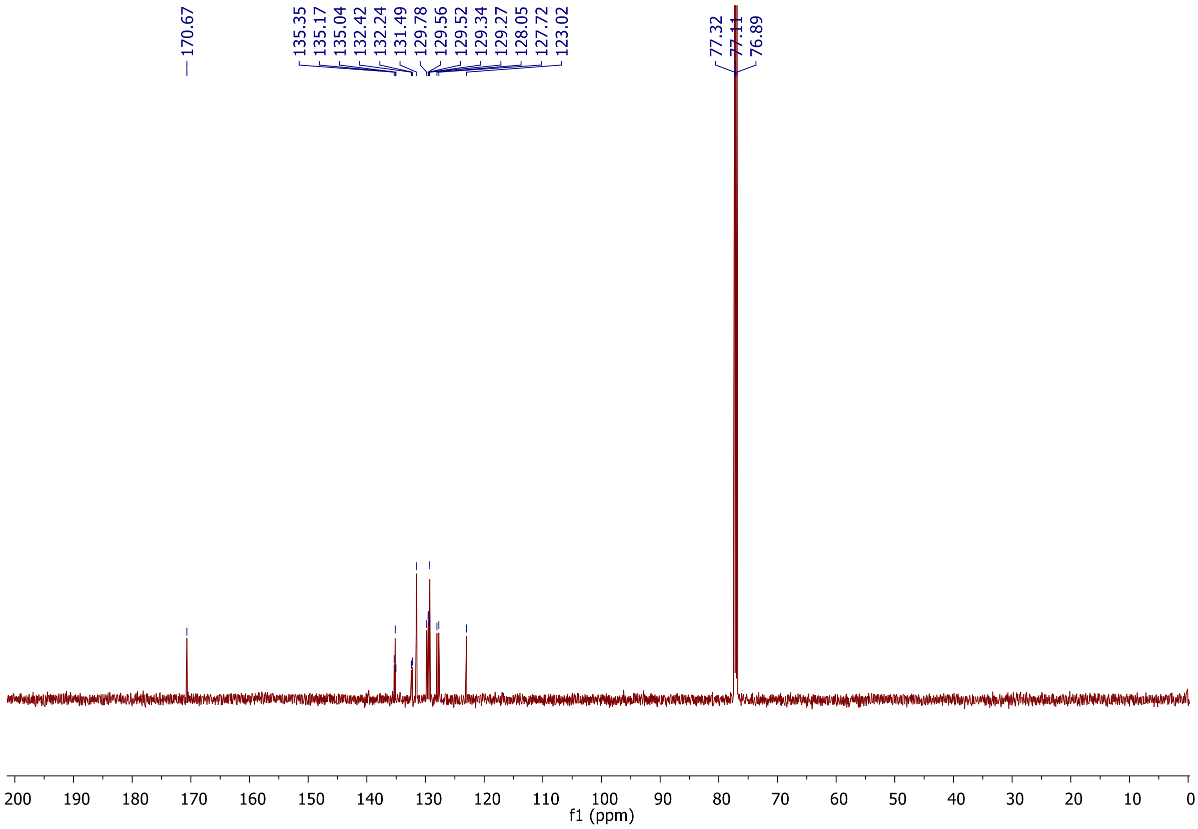

2l ^{1}H NMR (600 MHz, $CDCl_3$)

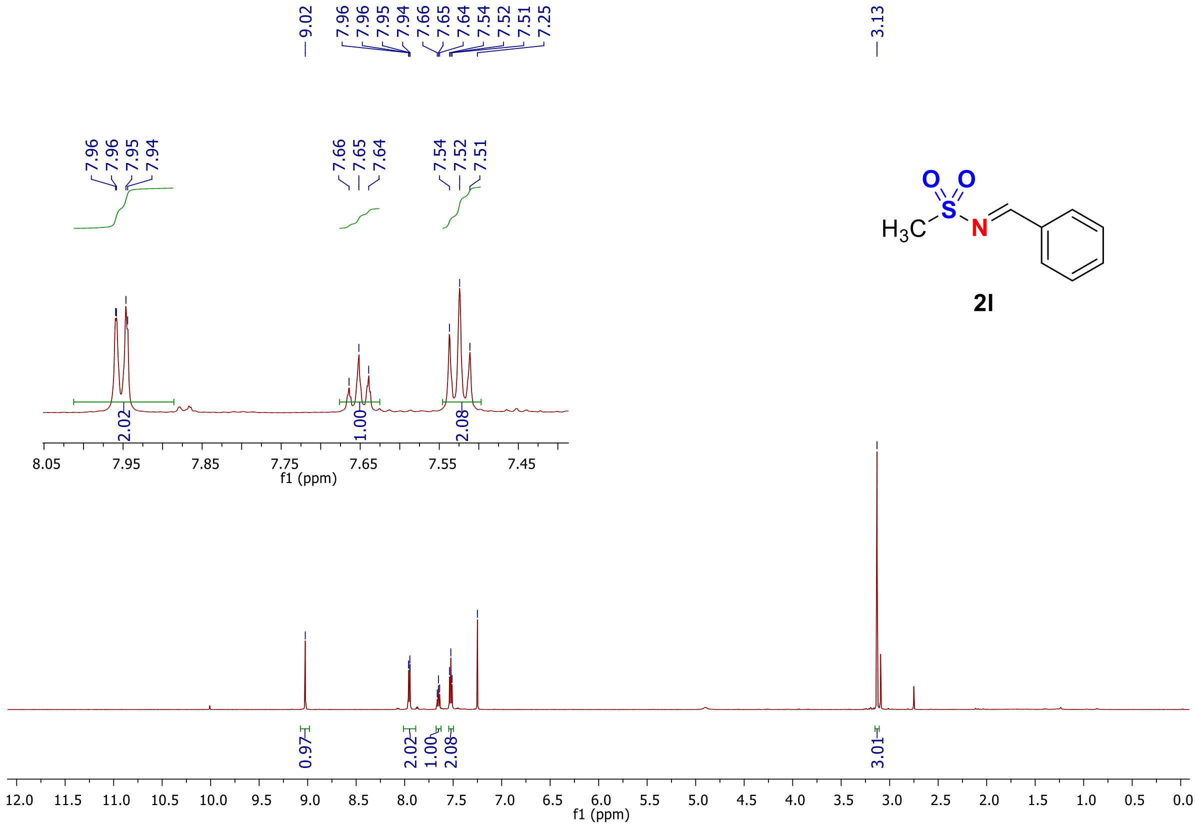

2l

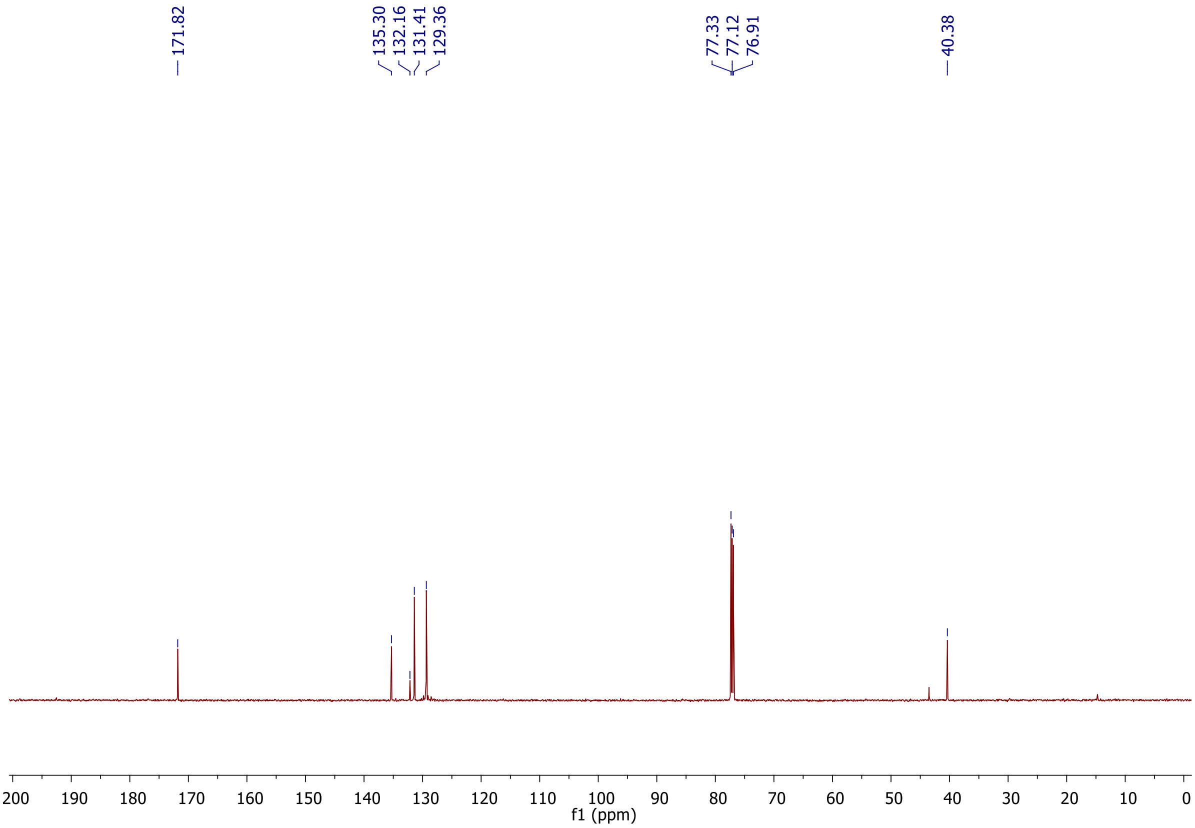

4a ^{1}H NMR (600 MHz, $CDCl_3$)

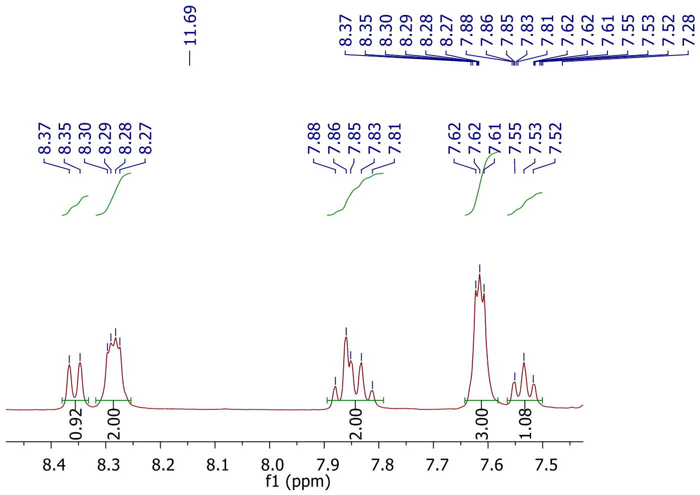

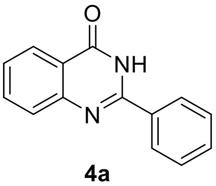

4a

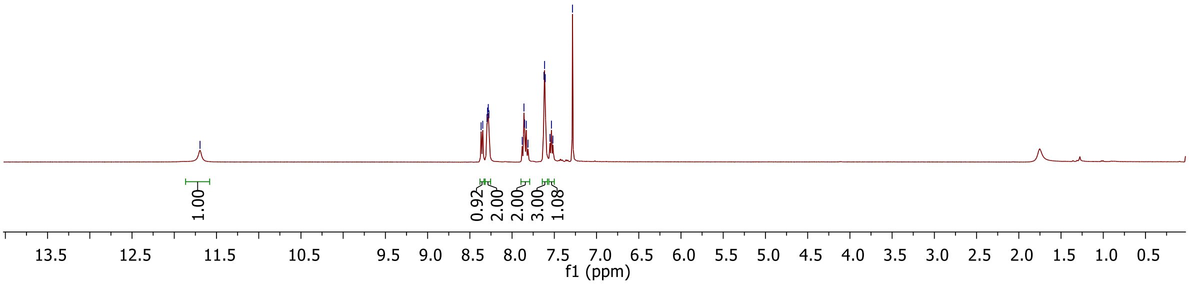

^{13}C{^{1}H} NMR (151 MHz, $CDCl_3$)

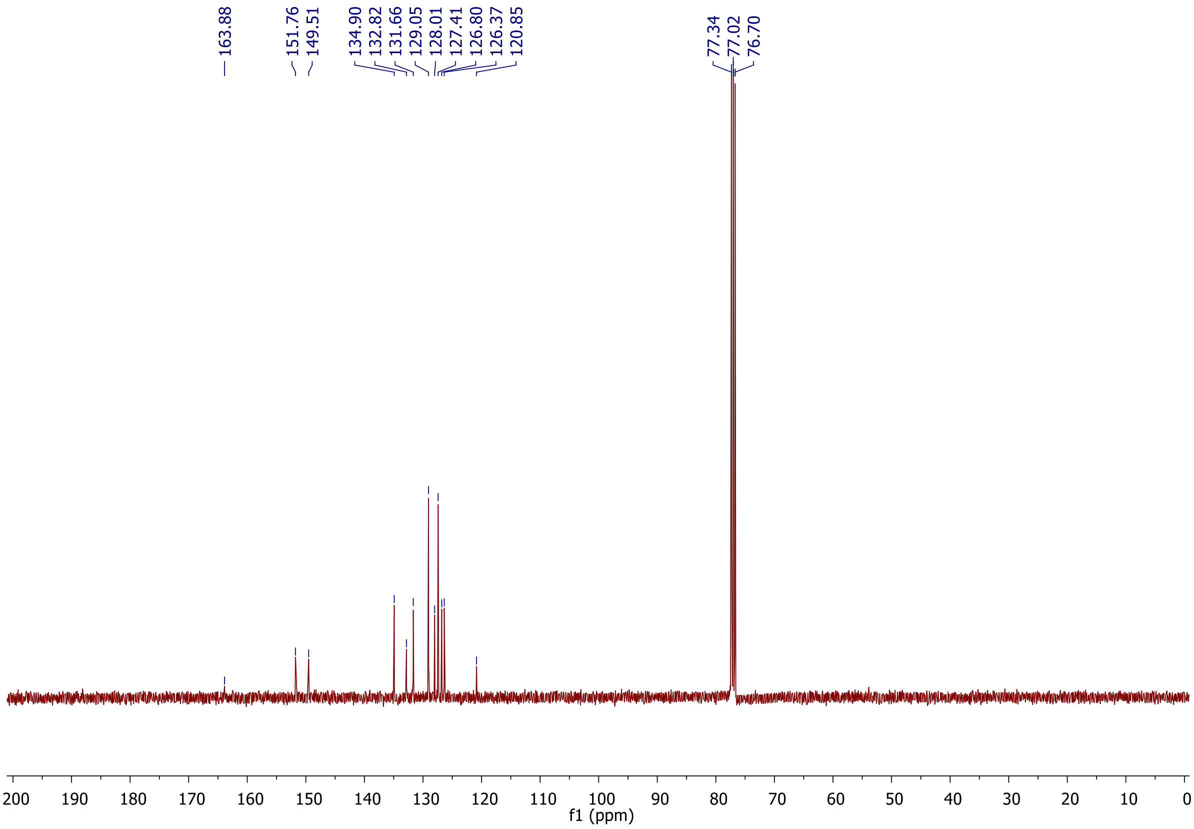

4b ^{1}H NMR (600 MHz, $CDCl_3$)

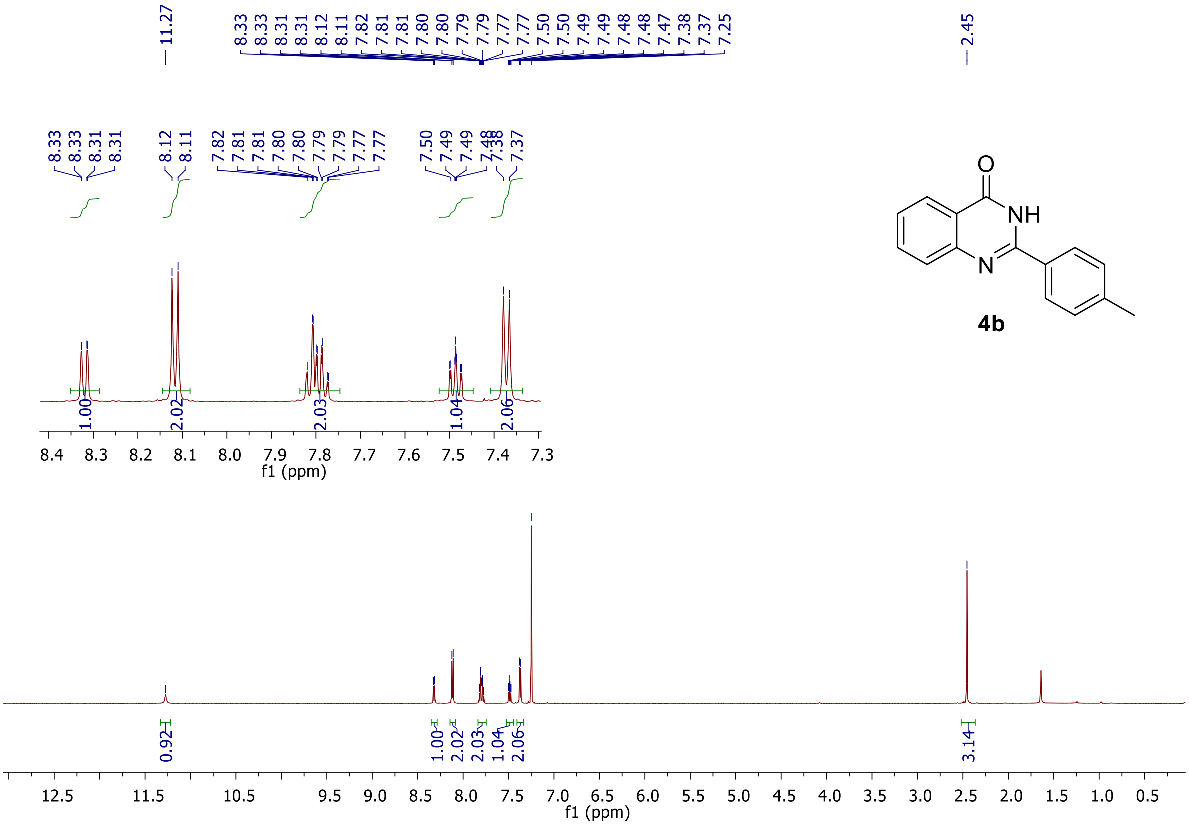

4b

^{13}C{^{1}H} NMR (151 MHz, $CDCl_3$)

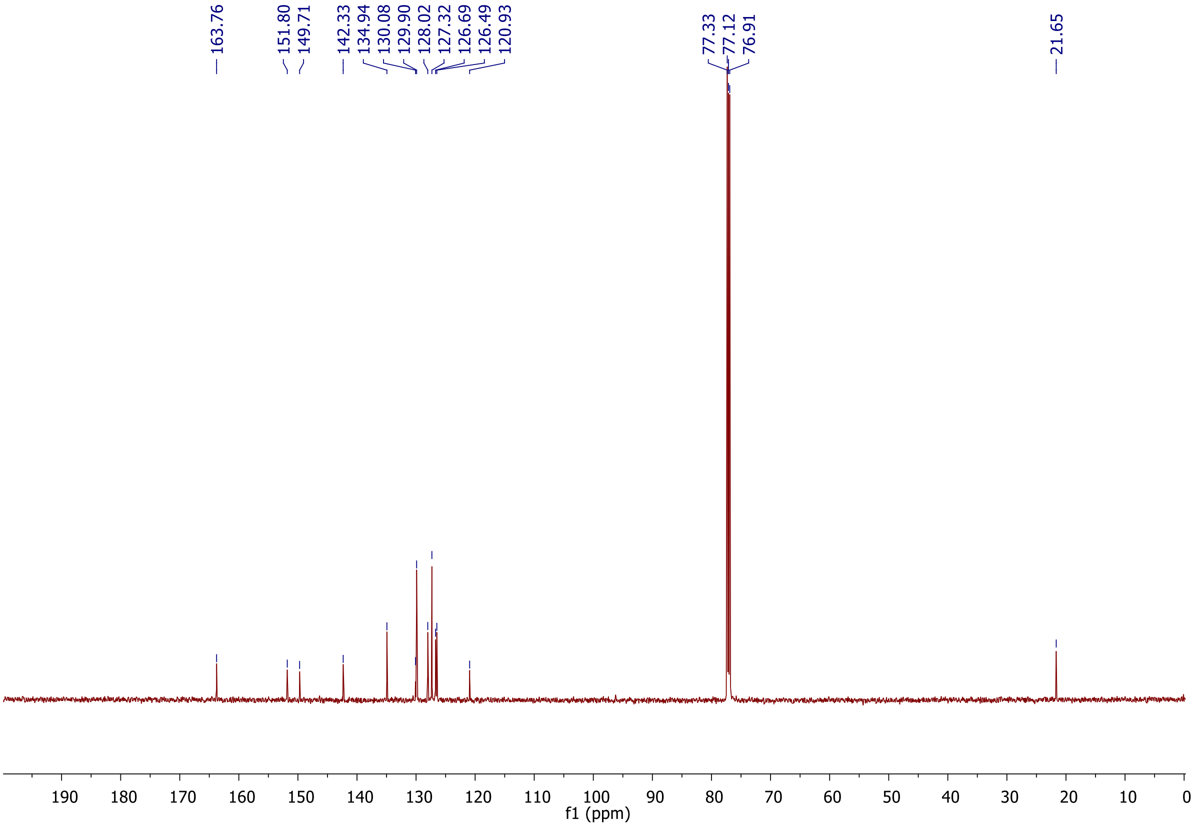

4c ^{1}H NMR (600 MHz, $CDCl_3$)

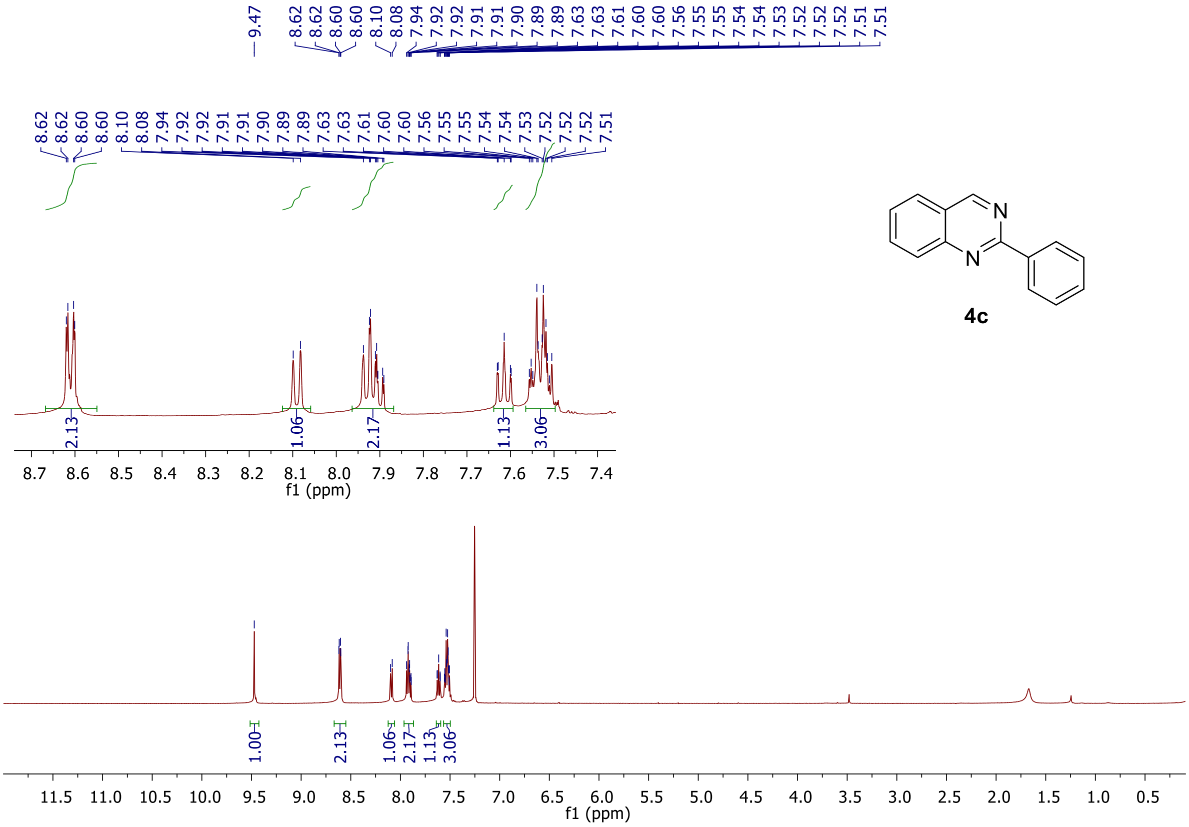

4c

^{13}C{^{1}H} NMR (151 MHz, $CDCl_3$)

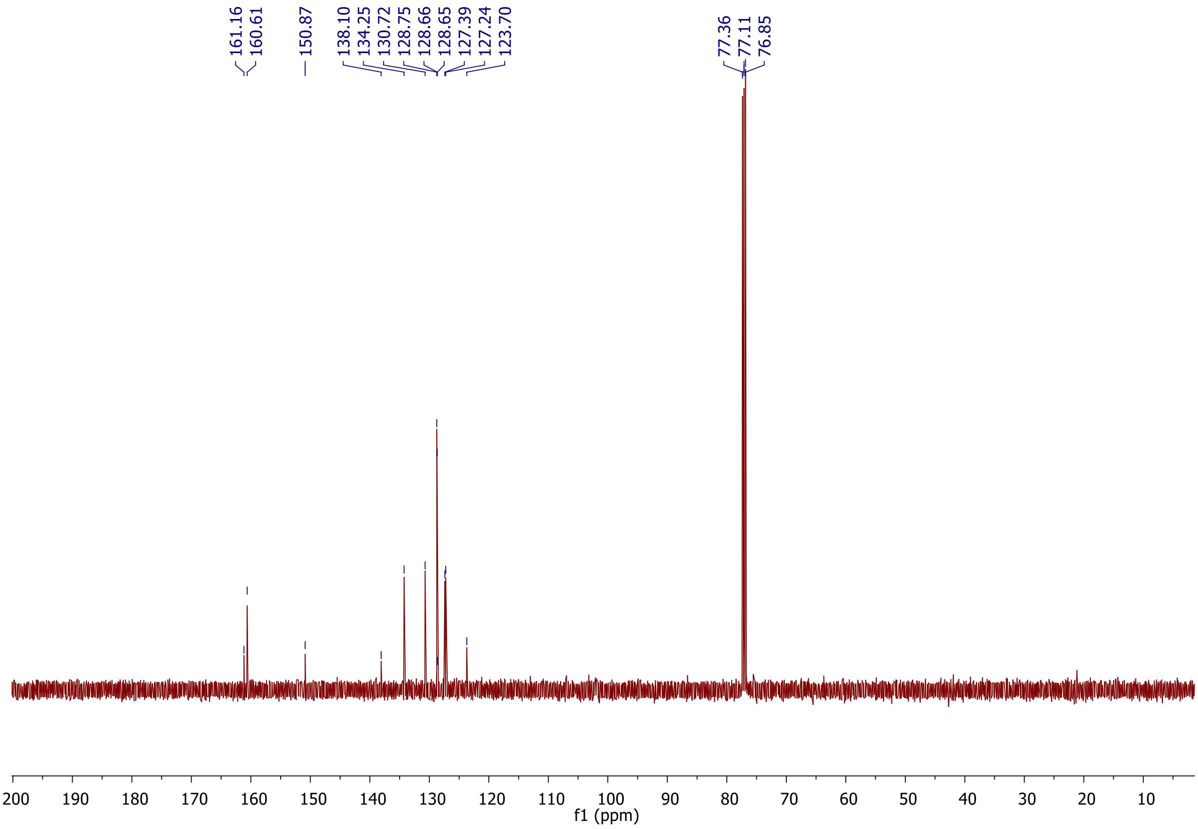

4d ^{1}H NMR (600 MHz, $CDCl_3$)

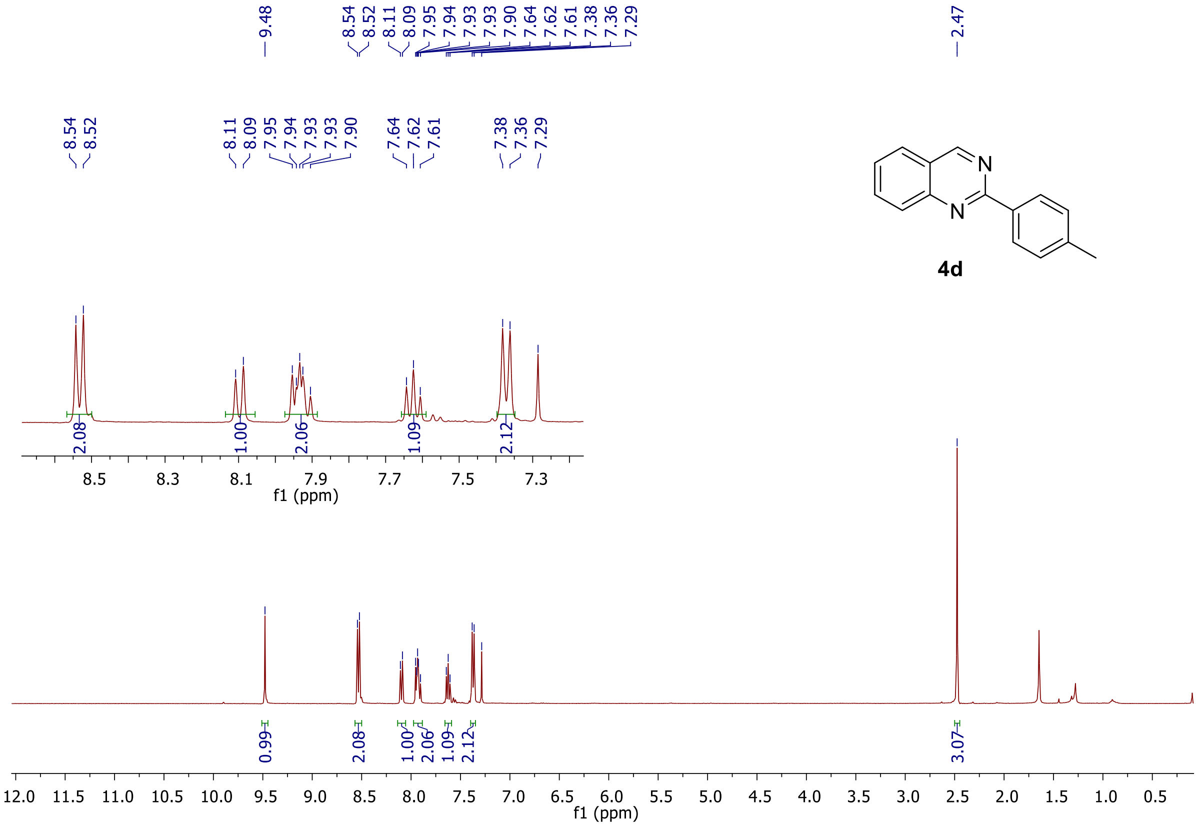

4d

$^{13}C\{^{1}H\}$ NMR (151 MHz, $CDCl_3$)

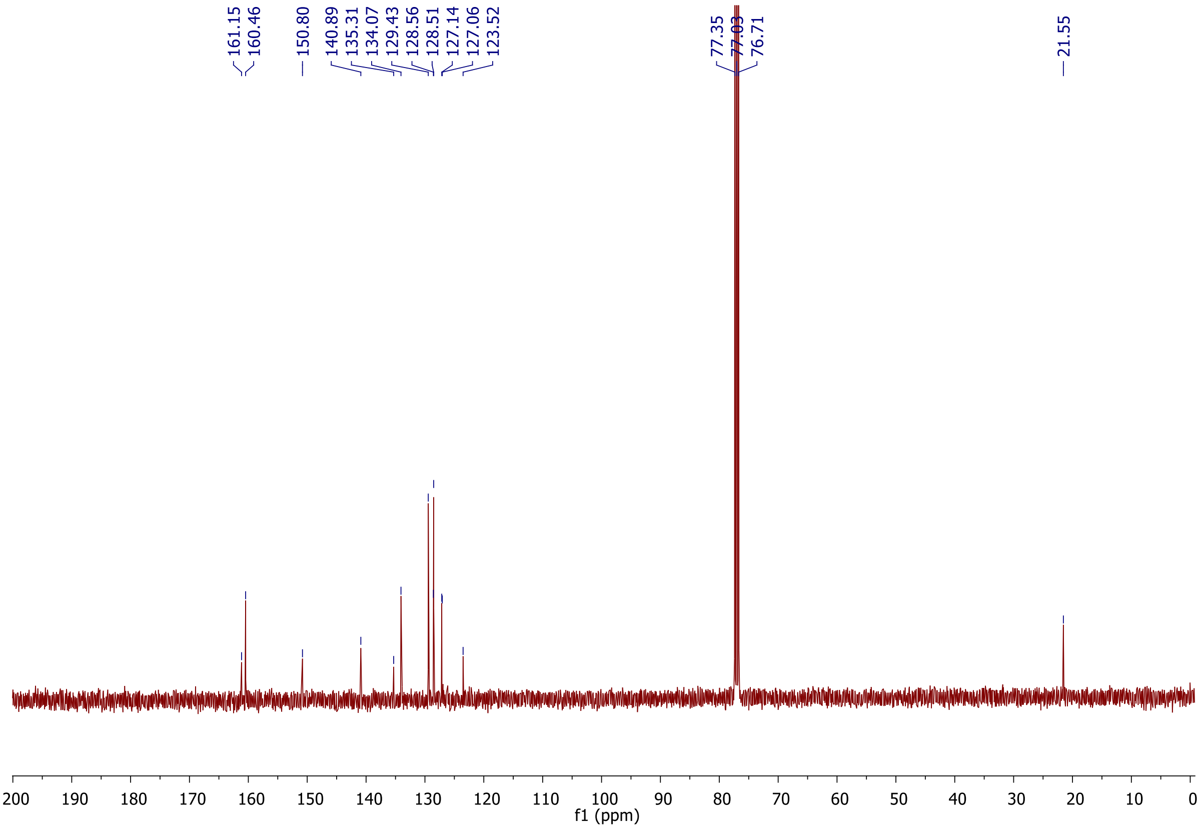

4e ^{1}H NMR (600 MHz, $CDCl_3$)

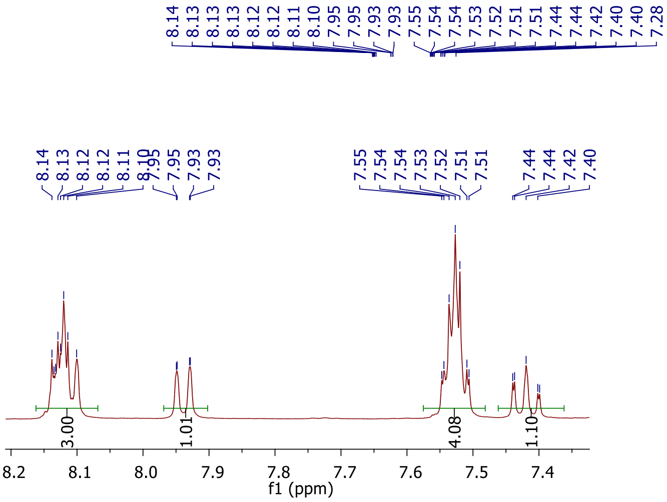

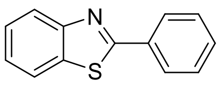

4e

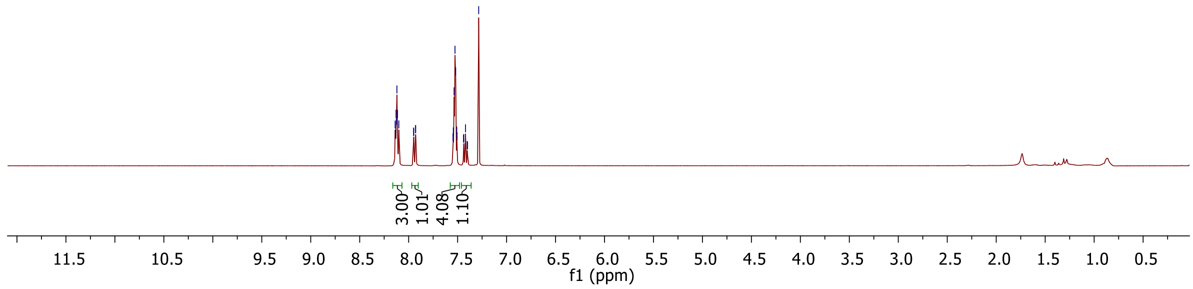

^{13}C{^{1}H} NMR (151 MHz, $CDCl_3$)

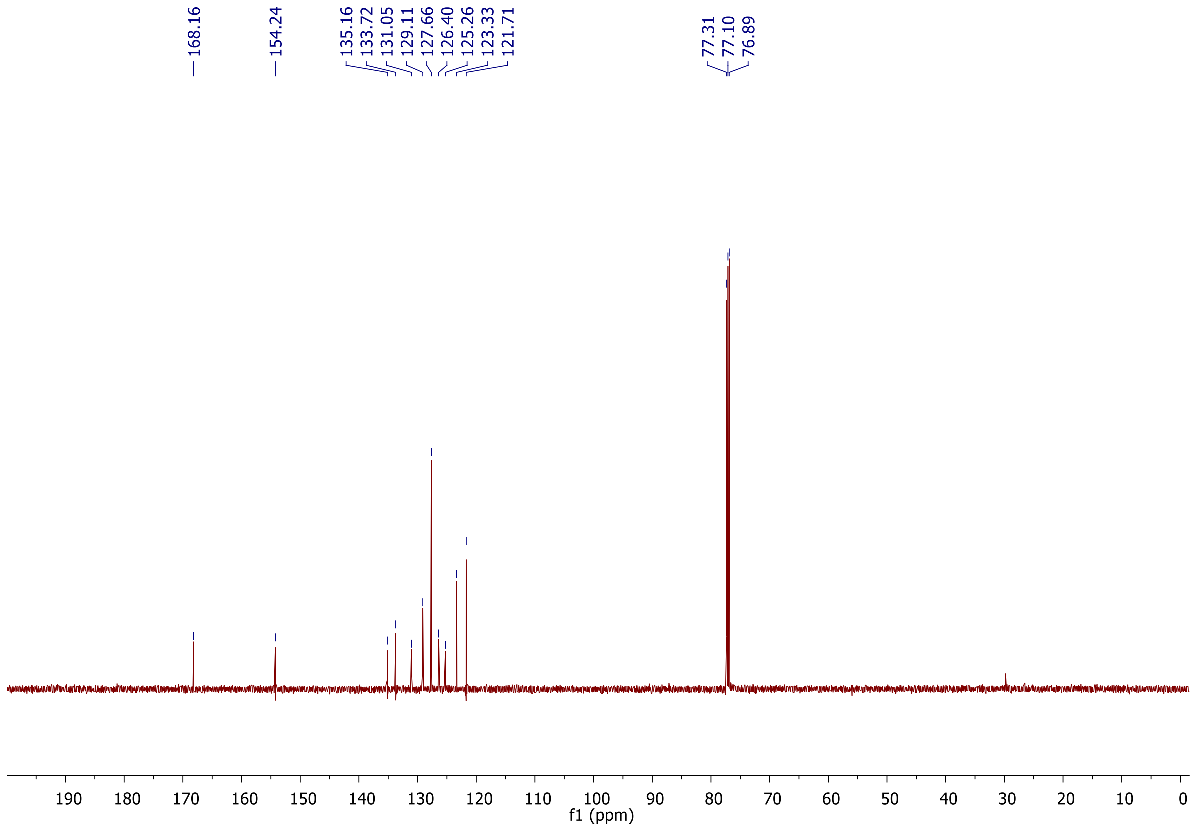

4f ^{1}H NMR (600 MHz, DMSO-d_6)

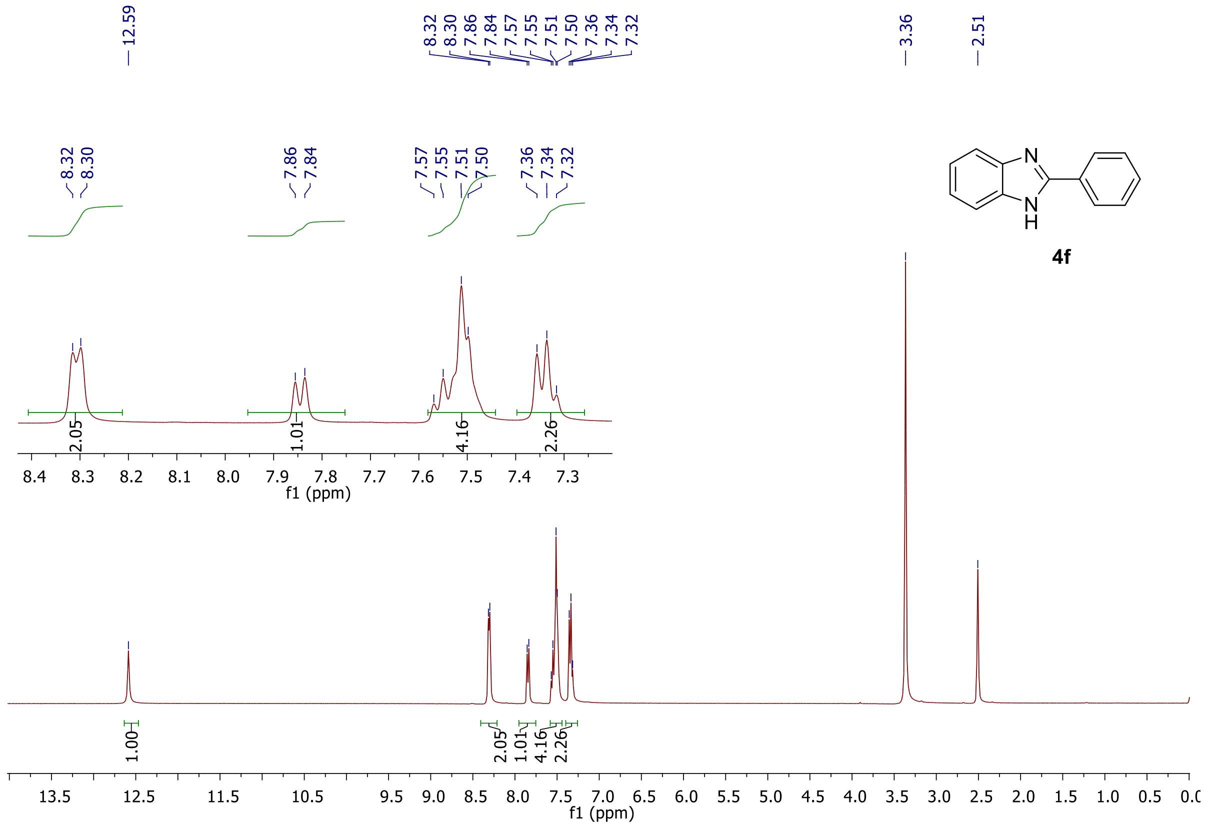

^{13}C{^{1}H} NMR (151 MHz, DMSO-d_6)

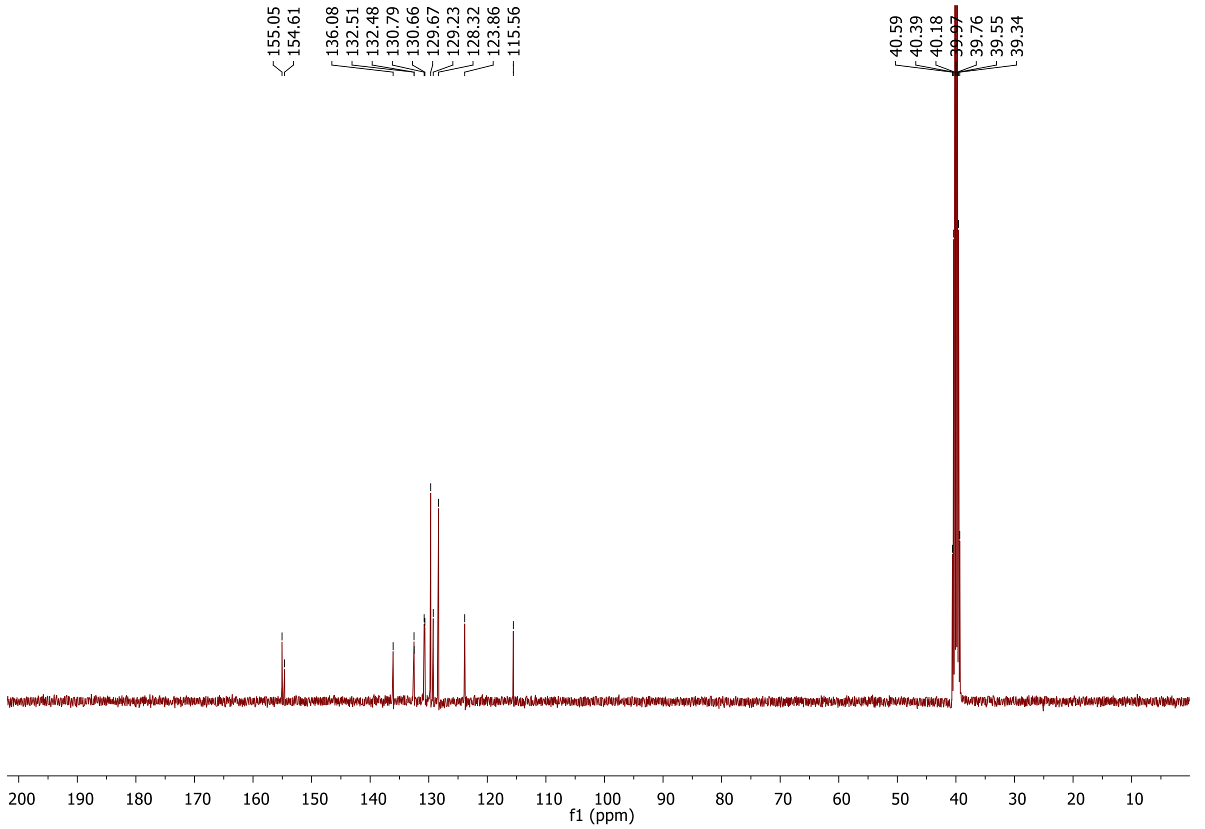

Supplement: File 1 — General procedures, product characterization, and copies of 1H NMR and 13C NMR spectra of all compounds. [file Beilstein_J_Org_Chem-19-771-s001.pdf]
